# Extracellular vesicles from monocyte/platelet aggregates modulate human atherosclerotic plaque reactivity

**DOI:** 10.1002/jev2.12084

**Published:** 2021-04-27

**Authors:** Silvia Oggero, Monica de Gaetano, Simone Marcone, Stephen Fitzsimons, Andreia L. Pinto, Dinara Ikramova, Mary Barry, David Burke, Trinidad Montero‐Melendez, Dianne Cooper, Thomas Burgoyne, Orina Belton, Lucy V. Norling, Eoin P. Brennan, Catherine Godson, Mauro Perretti

**Affiliations:** ^1^ William Harvey Research Institute Bart's and the London School of Medicine Queen Mary University of London London UK; ^2^ Diabetes Complications Research Centre Conway Institute, & School of Medicine University College Dublin Dublin Ireland; ^3^ Trinity Translational Medicine Institute Trinity College Dublin Dublin Ireland; ^4^ Royal Brompton & Harefield NHS Foundation Trust London UK; ^5^ School of Engineering and Materials Science Queen Mary University of London London UK; ^6^ Department of Vascular Surgery St. Vincent's University Hospital Dublin Ireland; ^7^ Centre for inflammation and Therapeutic Innovation Queen Mary University of London London UK; ^8^ Institute of Ophthalmology, Faculty of Brain Sciences University College London London UK

**Keywords:** extracellular vesicles, monocyte/platelet aggregates, proteomics, vascular inflammation

## Abstract

Extracellular vesicles (EVs) are emerging as key players in different stages of atherosclerosis. Here we provide evidence that EVs released by mixed aggregates of monocytes and platelets in response to TNF‐α display pro‐inflammatory actions on endothelial cells and atherosclerotic plaques. Tempering platelet activation with Iloprost, Aspirin or a P2Y_12_ inhibitor impacted quantity and phenotype of EV produced. Proteomics of EVs from cells activated with TNF‐α alone or in the presence of Iloprost revealed a distinct composition, with interesting hits like annexin‐A1 and gelsolin. When added to human atherosclerotic plaque explants, EVs from TNF‐α stimulated monocytes augmented release of cytokines. In contrast, EVs generated by TNF‐α together with Iloprost produced minimal plaque activation. Notably, patients with coronary artery disease that required percutaneous coronary intervention had elevated plasma numbers of monocyte, platelet as well as double positive EV subsets. In conclusion, EVs released following monocyte/platelet activation may play a potential role in the development and progression of atherosclerosis. Whereas attenuating platelet activation modifies EV composition released from monocyte/platelet aggregates, curbing their pro‐inflammatory actions may offer therapeutic avenues for the treatment of atherosclerosis.

**Abbreviations**
ELISA: enzyme linked immunoassay
EV: extracellular vesicle
FBS: foetal bovine serum
FMO: fluorescence minus one
ImageStream™: IS^x^
LC‐MS: liquid chromatography‐mass spectrometry
PA: pathways analysis
PBMCs: peripheral blood mononuclear cells
PBS: phosphate buffer saline
PGI_2_: prostaglandin I_2_
PRP: platelet rich plasma
TNF‐α: Tumour Necrosis Factor‐α.
T‐PBS: PBS with 0.1% Triton

## 1 INTRODUCTION

Extracellular vesicles (EVs) are cell‐borne particles that contain a complex biological cargo composed of nucleic acids, proteins and lipids. First described by Wolf in 1967, EVs were reported to have prothrombotic functions (Hargett & Bauer, 2013). Since then, EVs have been described to have several properties with functional consequences in physio‐pathology processes, including modulation of the adaptive immune response (Raposo et al., 1996), tumorigenesis (Pucci et al., 2016), and coagulation (Wei et al., 2018). The majority of work conducted so far with EVs has focused on identifying markers of the cell of origin; however we have proposed that their composition and hence properties would vary to reflect the environment surrounding the cell source (Dalli et al., 2013).

Atherosclerosis is the most prominent and common cause of cardiovascular diseases responsible for ∼50% of all deaths in Europe (Nichols et al., 2012). A phenomenon identified in atherosclerosis and other vascular diseases is formation of leukocyte and platelet aggregates within the circulation. In particular, increased numbers of monocyte/platelet aggregates are reported in patients with atherosclerosis (Furman et al., 1998, 2001) or atherothrombosis (Neumann et al., 1999), and animal models have unravelled some of the molecular mechanisms underlying this inter‐cellular interaction (Del Conde et al., 2005; Patko et al., 2012). While presence of these aggregates has been used as a possible biomarker, there is lack of investigation about their possible function especially in relation to the EVs which they can release. This could be relevant, since EVs can cause endothelial dysfunction, vascular calcification, unstable plaque progression, rupture and thrombus formation (Jansen et al., 2017).

In the context of plaque formation and destabilization, studies have focused on vesicles released from the plaques. For example, atherosclerotic plaque EVs expressed surface antigens of leukocyte origin (including major histocompatibility complex classes I and II), and promoted T‐cell proliferation (Mayr et al., 2009). In terms of EVs effects once added to the plaque, there is in vivo evidence for monocyte EVs to promote leucocyte adhesion to post‐capillary venules and T‐cell infiltration within the plaque (Hoyer et al., 2012). The majority of these studies have been conducted with murine models and in vitro cellular assays. However a better assessment of the inflammatory processes in human atherosclerosis can be attained through organ culture approaches, rather than using less complex experimental settings.

On these bases, we hypothesized that monocyte/platelet aggregates could produce EVs with specific pro‐inflammatory effects and that the composition of these vesicles could vary, and their properties tempered, by regulating platelet activation. Thus, we defined the composition of these EVs and their biological functions once added to endothelial cells and human atherosclerotic plaques. Finally, we detected specific subsets of EVs in patients affected by coronary artery disease. Altogether these data suggest that EVs could represent long‐term effectors of monocyte/platelet aggregates that exacerbate plaque activation and that anti‐platelet therapies could reduce their generation and downstream properties.

## 2 MATERIAL AND METHODS

### 2.1 Monocyte purification and flow cytometry characterization

All healthy volunteers gave written, informed consent to blood collection and the procedure was approved by the Queen Mary Ethics of Research Committee (QMERC2014.61). Blood (30 ml) was drawn using a 19G butterfly needle with tourniquet applied and anticoagulated with 0.32% w/v sodium citrate. To inhibit platelet activation, Iloprost (2 μM; stable prostacyclin analogue; Sigma‐Aldrich, Gillingham, UK) was added to whole blood prior to cell separation. In separate experiments, aspirin (30 μM; Sigma‐Aldrich) or clopidogrel (3 μM; Tocris, Bristol, UK) were used. Blood was centrifuged at 150 × *g* for 20 min and platelet rich plasma (PRP) removed and replaced with PBS+1 mM EDTA. Unless otherwise indicated, all experiments have been performed with PBS without calcium and magnesium. Following another centrifugation step, RosetteSep cocktail (15028, StemCell Technology, Vancouver, Canada) was added (50 μl/ml of blood) and samples rested at room temperature for 20 min. Blood was then diluted 1:1 with PBS+1μM EDTA and layered over 15 ml Histopaque 1077 (Sigma‐Aldrich, Gillingham, UK), centrifuged for 20 min at 1200x*g* room temperature to separate monocytes from other cells. The monocyte layer was harvested and washed at 300x*g* for 10 min. Following another washing step, the monocyte pellet was re‐suspended in phenol red‐free RPMI (Gibco, Waltham, US) and the concentration adjusted as needed.

For peripheral blood mononuclear cell (PBMC) isolation, whole blood was centrifuged at 130 × *g* for 20 min and plasma removed. For every 30 ml of whole blood, erythrocytes were depleted by sequentially layering 10 ml PBS followed by 8 ml of 6% w/v dextran (high molecular weight, Sigma‐Aldrich, in PBS) and gently inverting. After 15 min, the leukocyte‐rich fraction was collected and layered over Histopaque 1077 and centrifuged for 30 min 450 × *g* at room temperature to separate granulocytes from PBMC. PBMCs were washed once by centrifuging at 300 × *g* and re‐suspended in RPMI for further use. Blood aliquots (100 μl) were stimulated with TNF‐α (50 ng/ml; T0157‐10UG, Sigma‐Aldrich) or vehicle (PBS) for 1 h at 37 °C. After stimulation, erythrocytes were lysed with lysing reagent kit (6602764, Beckman Coulter) and samples prepared for IS^x^ analysis.

After isolation, cells were treated with Fc receptor blocking solution and stained with anti‐CD14‐APC (2 μg/ml, 61D3; Thermo Fisher Scientific, San Diego, USA; Cat#17‐0149‐73, RRID: AB_469354), anti‐CD41‐PE (2 μg/ml, HIP8; Biolegend, San Diego, USA; Cat# 303706, RRID:AB_314376), anti P‐selectin‐FITC (2.5 μg/ml, AC1.2; Becton Dikinson, Biolegend, USA; Cat# 328806, RRID:AB_2185243). Cells were acquired on an LSR Fortessa cytometer.

For platelet isolation, PRP was processed into washed platelets (WP) by addition of 2 μg/ml Iloprost and 0.02 U/ml apyrase (M0398S, NEB), prior to centrifugation at 1000*xg* for 10 min. After one wash, platelets where counted, and concentration was adjusted to 3 × 10^8^/ml before stimulating.

### 2.2 Fluorescent microscopy analysis of monocytes and platelets

Isolated monocytes containing platelets were spotted on Alcian blue‐coated glass slides and fixed in cold 4% paraformaldehyde (4°C, 30 min). After fixation, cells were washed with PBS and then blocked in PBS with 0.2% BSA (for surface staining) or PBS with 0.1% Triton and 0.2% BSA (T‐PBS; for intracellular staining) for 30 min at room temperature shaking. Following blocking, monocytes and platelets were incubated with primary specific antibodies against Annexin A1 (AnxA1; 5 μg/ml; clone 1B, in house generated) and Gelsolin (GSN; 1.54 μg/ml clone EPR1942; Abcam, Cambridge, UK; Cat#ab109014, RRID:AB_10863643) in either PBS+0.2% BSA or T‐PBS+0.2% BSA overnight at 4°C. Cells were washed and incubated with secondary antibody Alexa Fluor 488 anti‐rabbit (5 μg/ml, Molecular Probes Invitrogen, Eugene, USA; Cat#A‐11008, RRID:AB_143165) or Alexa Fluor 594 anti‐mouse (5 μg/ml, Molecular Probes Invitrogen; Cat# A‐11032, RRID:AB_2534091) in T‐PBS+0.2% BSA for 1 h at 20°C shaking. Cells were then mounted with a glass coverslip using Fluoroshield Histology Mounting Medium with DAPI (Sigma‐Aldrich) and visualized under the microscope Zeiss LSM800 Imaging System.

### 2.3 Western blot analysis of monocytes and platelets

Presence of GSN and AnxA1 was confirmed through standard SDS‐PAGE (Millipore, Watford, UK), loading 1–30 μg extracts from monocyte or washed platelet lysates. Western blot was conducted with specific antibodies against Annexin A1 (AnxA1; 5 ng/ml; clone 1B), GSN (1.54 ng/ml clone EPR1942), Galectin‐9 (Gal‐9; 0.5 μg/ml; clone AF2045; R&D, Minneapolis, USA; Cat# AF2045, RRID:AB_2137232) or anti‐β‐actin (ACTB; 5 ng/ml; clone AC‐74, Sigma‐Aldrich; Cat# A5316, RRID:AB_476743) overnight at 4°C followed by 1 h incubation with either an HRP‐conjugated goat anti‐mouse IgG or goat anti‐rabbit IgG (Dako, Cambridge, UK; Cat# E0433, RRID:AB_2687905 and Cat# K4003, RRID:AB_2630375 respectively). Proteins were detected using Luminata Forte Western HRP Substrate (Millipore, Watford, UK) visualized on Hyperfilm (GE Healthcare, Buckinghamshire, UK).

### 2.4 Generation and isolation of monocyte/platelet and plasma EVs

Monocyte or platelet preparations, freshly prepared and with or without TNF‐α stimulation, were centrifuged at 4400 × *g* at 4°C for 15 min, followed by a second centrifugation at 13,000 × *g* at 4°C for 2 min to remove remaining contaminants (e.g. apoptotic bodies). EVs were enriched by centrifuging at 20,000 × *g* at 4°C for 30 min, supernatants removed, and finally pellets re‐suspended in filtered sterile PBS. An identical procedure was used for plasma samples. Full ethical approval was obtained from the Research Ethics Committee at The Beacon Hospital, Dublin, Ireland for the collection of blood samples from patients prior to cardiac angiogram (Study Reference Number: BEA0121). The study was carried out in accordance with the World Medical Association's Declaration of Helsinki. All patients gave informed written consent.

### 2.5 Characterization of monocyte‐derived EVs

#### 2.5.1 Nanoparticle tracking analysis for sizing EVs

Approximately 0.5 ml of EV preparations (between 10^6^ to 10^8^ vesicles) were loaded onto the Nanosight NS300 with 488 nm scatter laser and high sensitivity camera (Malvern Instruments Ltd., Malvern, UK); five videos of 90 s each were acquired for each sample. When analyses required fluorescence detection, samples were preincubated with anti‐CD14‐AlexaFluor 488 (1 μg/ml, M5E2; Biolegend), anti‐CD41‐AlexaFluor 488 (2 μg/ml, HIP8; Biolegend) and data were acquired applying the 488 nm band pass filter to detect fluorescent events. Data analysis was performed with NTA2.1 software (Nanosight, Malvern, UK). Software settings for analysis were the following, Detection Threshold: 5–10; Blur: auto; Minimum expected particle size: 20 nm.

*ImageStream (IS^X^) analysis for quantification and characterisation of EVs*. EVs were analysed and counted using fluorescence triggering on an IS^X^ MKII imaging cytometer as described previously (Headland et al., 2015). Briefly, vesicles were labelled with 50 μM BODIPY maleimide fluorescein or BODIPY Texas‐Red (Life Technologies, Carlsbad, USA; Cat# A‐5770, RRID:AB_2536193), and acquired as such or after labelling with either 2 μg/ml anti‐CD14‐APC (61D3; Biolegend), 2 μg/ml anti‐CD41‐PE (HIP8; Biolegend) or one of the following Pacific Blue or Alexa Fluor 488 conjugated antibodies: anti‐Annexin A1 (AnxA1; 1 μg/ml; clone 1B), anti‐Gelsolin (GSN; 0.1 μg/ml clone EPR1942; Abcam). Fluorescence minus one (FMO) controls were used for gating all protein antigen‐positive events. Approximately 20,000 events were acquired per sample.

*Scanning Electron Microscopy (SEM) of monocyte/platelet aggregates and EVs*. Protocol was performed as previously published (Annaz et al., 2004). Briefly, monocyte/platelet aggregates (2 × 10^5^) were plated on glass coverslips stimulated with 50 ng/ml of TNF‐α for 30 min, fixed in cold 4% paraformaldehyde (4°C, overnight) to stop the EV release. Later samples underwent secondary fixation in 1% osmium tetroxide buffered in 0.1 M sodium cacodylate for 1 h at room temperature, followed by three 5 min washes in 0.1 M sodium cacodylate. Samples were then stained in 1% tannic acid buffered in 0.05 M sodium cacodylate, followed by two 5‐min washes in 0.1 M sodium cacodylate and then dehydrated in graded series of ethyl alcohol (20‐100%). The samples were air‐dried, mounted on aluminium stubs using conductive carbon tape, gold sputter coated for 30 s and imaged with FEI Inspect F Scanning Electron Microscope.

*Immunogold labelling and Transmission Electron Microscopy (TEM) of EVs*. Transmission electron microscopy (TEM) copper grids (400 mesh, Agar Scientific, Essex, UK) were pre‐coated with 1% formvar (Agar Scientific, Essex, UK) solution prepared in chloroform. EV suspension (1 × 10^8^/ml in 10 μL) was pipetted directly onto TEM grids; after 10 min, the TEM grids were washed, and fixed in 4% paraformaldehyde at 4°C for 20 min. Subsequently, grids were quenched with 20 mM glycine and incubated with specific antibodies against AnxA1 (5 ng/ml; clone 1B), GSN (1.54 ng/ml clone EPR1942; Abcam), or CD41 (5 ng/ml; clone EPR5788; Abcam, Cambridge, UK; Cat# ab133557, RRID:AB_2833020) for 1 h at room temperature. When using a primary antibody raised in mice, grids were further incubated with bridging antibody (rabbit‐anti‐mouse, Dako, Glostrup, DK) for 45 min. All grids were incubated with Protein Gold A (PGA; 10 nm or 5 nm sized gold, UMC Utrecht, NL), before washing in distilled water and fixing in 1% glutaraldehyde in PBS. Grids were finally stained in 2% uranyl acetate for 2 min. A JEOL 1400+ TEM (Tokyo, JPN) equipped with an AMT XR16 CCD camera (AMT, Massachusetts, USA) was used to acquire images taken between x 8000 and × 20,000 magnification.

#### 2.5.2 Proteomic analysis of EVs

EVs derived from monocytes treated with TNFα in presence or absence of Iloprost were pelleted at 20,000*xg* for 30 min, resuspended in 20 μl ice cold RIPA buffer containing protease inhibitor (Sigma Aldrich). Protein content from 5 distinct EV preparations was measured by spectrophotometry (Nanodrop 2000, ThermoFisher Scientific, Waltham, USA) selecting Protein A280 program and 50 μg of proteins were used for trypsin digestion. Mass spectrometry analysis of the proteins obtained from two technical replicates of EVs was performed on tryptic digests obtained using the Filter Aided Sample Preparation protocol as previously described (Wiśniewski et al., 2009). EVs proteome profile was determined by LC‐MS/MS analysis as previously described in the methods section. All data and materials have been made publicly at the PRIDE (Perez‐Riverol et al., 2019) Archive (EMBL‐EBI) with the dataset identifier PXD014325.

### 2.6 Western blotting analyses

Presence of a select group of proteins identified by proteomic analysis was confirmed through standard SDS‐PAGE, loading extracts from ∼30 × 10^6^ EVs per lane (Millipore, Watford, UK). Western blot was conducted with specific antibodies against AnxA1 (5 μg/ml), GSN (1.54 μg/ml; clone EPR1942; Abcam), CD9 (1 μg/m; System Biosciences; Cat# CD9A‐1), Heat shock protein β‐1 (HSPB1; 5 μg/ml; clone G3.1; Abcam; Cat# ab2790), Gal‐9 (0.5 μg/ml; clone AF2045; R&D), TSG101 (2 μg/ml, clone C2, Sigma‐Aldrich; Cat# T5701), Calnexin (1 μg/ml; Abcam; Cat# ab22595), or anti‐β‐actin (ACTB; 5 μg/ml; clone AC‐74, Sigma‐Aldrich) overnight at 4°C followed by a 1 h incubation with either an HRP‐conjugated goat anti‐mouse IgG or goat anti‐rabbit IgG (Dako). Proteins were detected using Luminata Forte Western HRP Substrate (Millipore) visualized on Hyperfilm (GE Healthcare).

### 2.7 Experiments with human umbilical vein endothelial cells (HUVEC) and human aortic endothelial cells (HAoEC)

Umbilical cords that were kindly donated by the midwifery staff of the Maternity Unit, Royal London Hospital (London, UK) with an approved protocol (East London & The City Local Research Ethics Committee reference 05/Q0603/34 ELCHA). HUVEC were isolated by collagenase digestion and used up to passage 4. HAoEC were donated by Dr Claudio Raimondi (William Harvey Research Institute) and used up to passage 6.

#### 2.7.1 Assessment of adhesion molecule expression by flow cytometry

HUVEC or HAoEC were grown to confluence in 6 well plate coated with 0.5% gelatin and stimulated 24 h with TNF‐α (10 ng/ml), or 10 × 10^6^ EVs isolated from monocyte‐platelet aggregates subsequent to stimulation with vehicle, TNF‐α, or iloprost+TNF‐α, in 0.5% exosome‐depleted human serum complete media. Following treatment with an Fc receptor blocking solution, cells were stained with anti‐ICAM‐1‐PE (1 μg/ml, HA58; Biolegend, San Diego, USA; Cat# 353106, RRID:AB_10897647), anti‐VCAM‐1‐BV711 (0.5 μg/ml, 5110C9; BD Optibuild, USA; Cat# 744312, RRID:AB_2742141). Cells were analyzed on an LSR Fortessa cytometer with > 10,000 events being acquired.

#### 2.7.2 Confocal imaging of EV uptake

HUVEC (1 × 10^5^) were seeded on 0.5% Gelatin coated μ‐Slide 8 Well Glass Bottom (80826, Ibidi) and then incubated with 1 × 10^6^ BODIPY‐FITC stained EV. Subsequently, cells were fixed with 4% PFA for 15 min and permeabilised with 0.1% Triton‐X for 1 h. Cells were finally stained with 1.5 nM Phalloidin AF647 (A22287, ThermoFisher Scientific; Cat# A22287, RRID: AB_2620155) for 45 min. A Nanoimager‐S microscope (ONI, UK) was used for microscopy of the HUVEC using ONI software. The following excitation/emission conditions were used in conjunction with x100 magnification oil immersion objectives: BODIPY 488/561 and AF647 640/658. The images acquired were analyzed using supplied ONI and ImageJ software packages.

### 2.8 Experiments with the human atherosclerotic plaque

*Isolation and ex‐vivo culture*. All five patients presented clinical and angiographic evidence of atherosclerosis prior to undergoing carotid or femoral endarterectomy; they gave written informed consent. The study was approved by the Ethics Committee of St. Vincent's University Hospital in Dublin, and in accordance with the International guidelines and Helsinki Declaration principles. Surgical atherosclerotic plaque samples were harvested in physiological saline. After dissection, they were stimulated in 24‐well plates for 24 h at 37°C, 5% CO_2_ in RPMI with 0.1% exosome depleted Foetal Bovine Serum with the different EV subsets (10 × 10^6^ per well), isolated from monocyte‐platelet aggregates subsequent to stimulation with vehicle, TNF‐α, or iloprost+TNF‐α as described above. After 24 h incubation, tissues samples and supernatants were collected and snap frozen in liquid nitrogen for subsequent analysis by mass spectrometry analysis and multiplex ELISA assay.

#### 2.8.1 Multiplex ELISA analysis of plaque and endothelial cell supernatants

GM‐CSF, IL‐6 and IL‐8 concentrations from HUVEC or HAoEC conditioned media or GM‐CSF, IFN‐γ, IL‐1β, IL‐4, IL‐6, IL‐10, IL‐13, MIP‐1α and TNF‐α concentrations from the centrifuged conditioned media of the human plaques were measured by enzyme immunoassay using commercially available human 96 well‐plate multiplex kit for tissue culture samples (MSD, Gaithersburg, USA) according to the manufacturers’ guidelines. Cytokine release from plaque was normalized by the total content of proteins measured in the supernatant by Nanodrop at 280 nm. The same cytokines plus MCP‐1 instead of TNFα were quantified in the monocyte conditioned media following removal of EVs by centrifugation.

## 3 STATISTICAL ANALYSIS

All statistical analysis and graphing were performed in GraphPad Prism 6 Software, IDEAS 6.2 for Image Stream Plots and FlowJo V6 for LSRFortessas Plots. Data are expressed as mean ± standard error (SEM) unless stated differently. Analyses applied to the different experimental data are indicated in each figure legend. A *P* value of < 0.05 was considered significant to reject the null hypothesis.

### 3.1 Data deposition and materials sharing

Mass spectrometry proteomics analysis of monocyte‐derived EVs: PRIDE PXD014324 (http://www.ebi.ac.uk/pride/archive/projects/ PXD014324).

## 4 RESULTS

### 4.1 Monocyte‐derived EVs are regulated by aggregation with platelets

**FIGURE 1:**
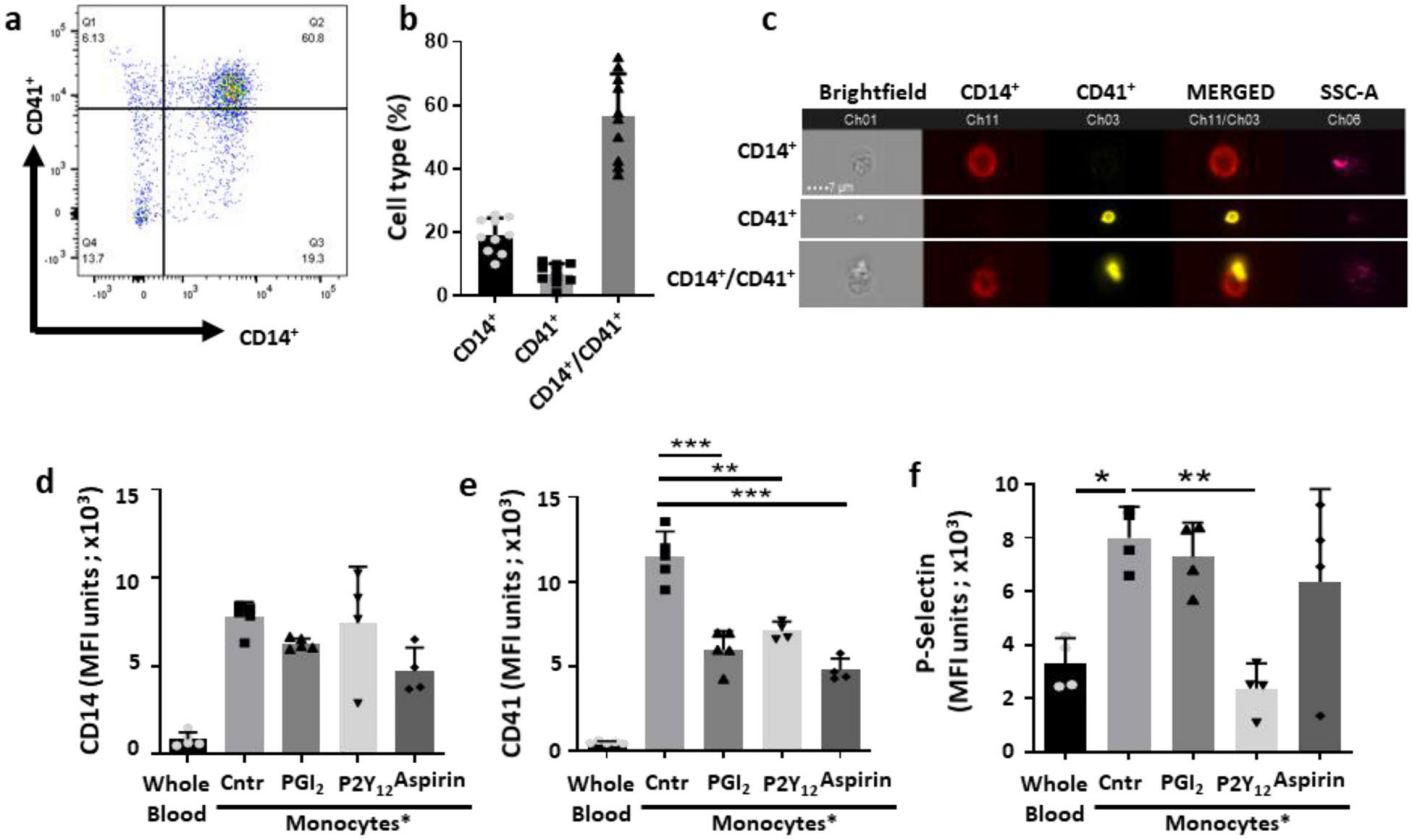
**Iloprost controls platelet, but not monocyte, activation. [**Whole blood aliquots or monocytes isolated using the RosetteSep purification protocol were incubated with or without 2 μM Iloprost (PGI_2_), 30 μM Aspirin or 3 μM P2Y_12_ inhibitor. CD14 and CD41 were used as markers for monocytes and platelets, respectively, for flow cytometry and Imagestream^x^ analyses. (a) Dot plot showing monocyte (CD14^+^) and platelet (CD41^+^) immunostaining to reveal presence of aggregates after RosetteSep purification (double positive events). (b) Percentages of CD14^+^ (monocytes), CD41^+^ (platelets), CD14^+^/CD41^+^ double positive (aggregates) events. Data are mean ± SEM of n = 10 distinct cell preparations. (c) Visualization of monocyte, platelet and monocyte/platelet aggregates by ImageStream^x^. Representative of n = 5 distinct cell preparations. (d) CD14 MFI units and (e) CD41 MFI units of the mixed monocyte/platelet aggregates as analysed by Imagestream^x^, comparing analysis of cells in the lysed whole blood (WB) or following purification of monocytes by RosetteSep, in the presence or absence of Iloprost, Aspirin and P2Y_12_ inhibitor. (f) P‐selectin expression of monocyte/platelet aggregates isolated with RosetteSep kit, following presence or absence of Iloprost, Aspirin and P2Y_12_ inhibitor compared to whole blood cells. (*P < 0.05, **P < 0.01, ***P < 0.001; one‐way ANOVA post Bonferroni test, mean ± SEM, n = 4 distinct preparations). *Monocyte refers to samples containing monocyte/platelet aggregates]

In order to determine the functional relevance of monocyte derived EVs in the context of atherosclerosis, an enriched population of monocytes was first prepared from human blood using a negative selection procedure. Flow cytometry analysis demonstrated a high degree of monocyte/platelet aggregates whereby 56.5±5.1% of CD14^+^ events were also positive for the platelet marker CD41 (n = 10; Figure 1a and 1b). This phenomenon was also visualized by IS^x^ where representative images indicate presence of single monocytes, single platelets as well as monocyte/platelet aggregates (Figure 1c). In order to determine whether platelet‐monocyte interactions were dependent on platelet activation, we introduced Iloprost; a stable analogue of prostacyclin, Aspirin or the P2Y_12_ inhibitor clopidogrel in our isolation protocol. Addition of all three drugs during the isolation procedure impacted CD41 MFI expression values on the aggregates (Figure 1e), with no effect on CD14 levels (Figure 1d). Notably, aggregation was not detected when monocytes were analyzed after lysing whole blood (Figure 1d,e and Figure S1).

When monocytes were isolated by density gradient isolation, following platelet rich plasma (PRP) removal, the formation of monocyte/platelet aggregates was minimal, yet aggregates could be obtained following stimulus addition, especially with platelet‐activating factor (PAF; Figure S1). Furthermore, increased aggregate formation correlated with a reduction of free platelets in the samples suggesting a degree of sequestration to form the mixed aggregates (Figure S1). Similar results were also observed when whole blood was stimulated with the same concentrations of TNF‐α and PAF (Figure S1). These data together suggest that monocyte/platelet aggregate formation results from the RosetteSep kit isolation procedure.

A degree of monocyte and platelet activation consequent to the purification procedure was confirmed by cell surface expression of P‐selectin compared to cells that were analyzed after whole blood cell lysis (Figure 1f). When cells were incubated with the P2Y_12_ inhibitor, P‐selectin levels remained similar to baseline suggesting an involvement of this receptor in the aggregate formation. In comparison, P‐selectin levels were only marginally decreased when aggregates were treated with Iloprost or Aspirin, suggesting these drugs may have an alternate mechanism for attenuating the interaction between the two cell types. Since monocyte/platelet aggregates are typical of several cardiovascular settings, including atherosclerosis (see Discussion), we decided to exploit this enriched monocyte preparation to study formation and properties of EVs generated in these cell‐to‐cell crosstalk settings.

**FIGURE 2:**
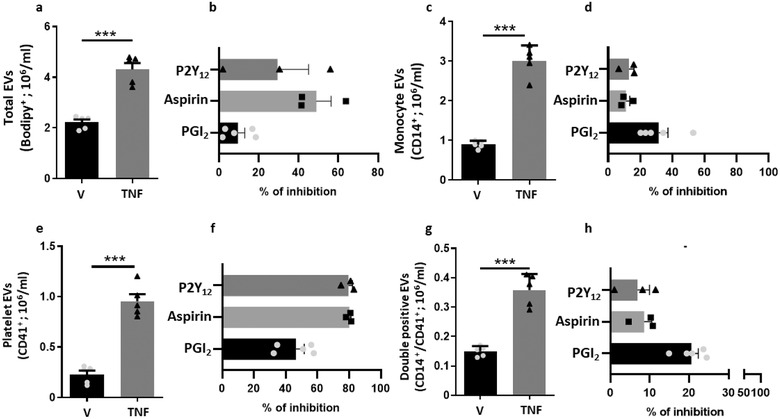
**Monocyte/platelet‐derived EVs are released upon TNF‐stimulation**. [Monocytes were isolated using the RosetteSep purification protocol. Cells (1 × 10^6^/ml) were incubated with vehicle (V) or TNF‐α (50 ng/ml), in presence or absence of Iloprost (2 μM; PGI_2_), Aspirin (30 μM) or P2Y_12_ inhibitor (3 μM) for 60 min. EV generation in cell‐free supernatants was quantified following Bodipy staining for (b) total vesicles. (c) Percentage of inhibition of total EV release upon stimulation with TNF‐ α in presence of PGI_2_, Aspirin or P2Y_12_ inhibitor. (c) Monocyte CD14^+^ EVs concentrations in response to TNF‐α stimulation and % of inhibition of CD14^+^ EVs release upon stimulation with TNF‐α in presence of PGI_2_, Aspirin, P2Y_12_ inhibitor. (e) Platelet CD41^+^ EVs concentrations and % of inhibition of CD41^+^ EVs release upon stimulation with TNF‐α in presence of PGI_2_, Aspirin, P2Y_12_ inhibitor (f). (g) Double positive CD14^+^/CD41^+^ vesicles concentrations and % of inhibition of CD14^+^/CD41^+^ EVs release upon stimulation with TNF‐α in presence of PGI_2_, Aspirin, P2Y_12_ inhibitor. (*P < 0.05, **P < 0.01, ***P < 0.001; one‐way ANOVA post Bonferroni test, mean ± SEM, n = 3–5 distinct preparations)]

For a more detailed analysis of EVs, we implemented a validated protocol where fluorescence triggering of EVs (labelled with BODIPY‐FITC) allows a better identification by IS^x^ (Headland et al., 2015). Using a double gating strategy for staining with CD14^+^ and CD41^+^, EVs from platelets, monocytes and a subset bearing both markers were monitored, both in the presence and absence of TNF‐α and drugs (Figure 2). TNF‐α addition to monocytes almost doubled the number of total EVs compared with unstimulated cells (n = 5, *P* < 0.01) (Figure 2a). Addition of Iloprost reduced TNF‐α induced EV release by approximately 15%, whereas Aspirin and the P2Y_12_ inhibitor reduced the total number of EVs by ∼50% and 30%, respectively (Figure 2b). Similarly, Aspirin and the P2Y_12_ inhibitor had a greater effect on platelet CD41^+^/CD14^–^ EVs (more than 80% inhibition), while Iloprost reduced numbers by around 45% (Figure 1f). Comparatively, Iloprost had a more pronounced effect on both TNF‐α stimulated CD14^+^/CD41^–^ and CD14^+^/CD41^+^ EV subsets (∼30% and 20%, respectively) than Aspirin and the P2Y_12_ inhibitor (both reducing CD14^+^/CD41^–^ by ∼20% and CD14^+^/CD41^+^ by ∼10%) (Figure 2d,h). Having established its predominant modulation on those particular subsets of EVs, further experiments were carried out only upon addition of Iloprost.

To understand if detection of double positive EVs (Figure 2g) was an artificial result of the swarming effect, a known problem during EV analysis by flow cytometry (Libregts et al., 2018), serial dilutions of EV samples isolated from monocyte/platelet aggregates treated with TNF‐α were analyzed. Whilst total numbers of Bodipy positive EVs were reduced due to the dilution of the samples, percentages of the monocyte, platelet and more importantly double positive EVs were not affected (Figure S2), indicating swarming did not occur in our experiments.

**FIGURE 3:**
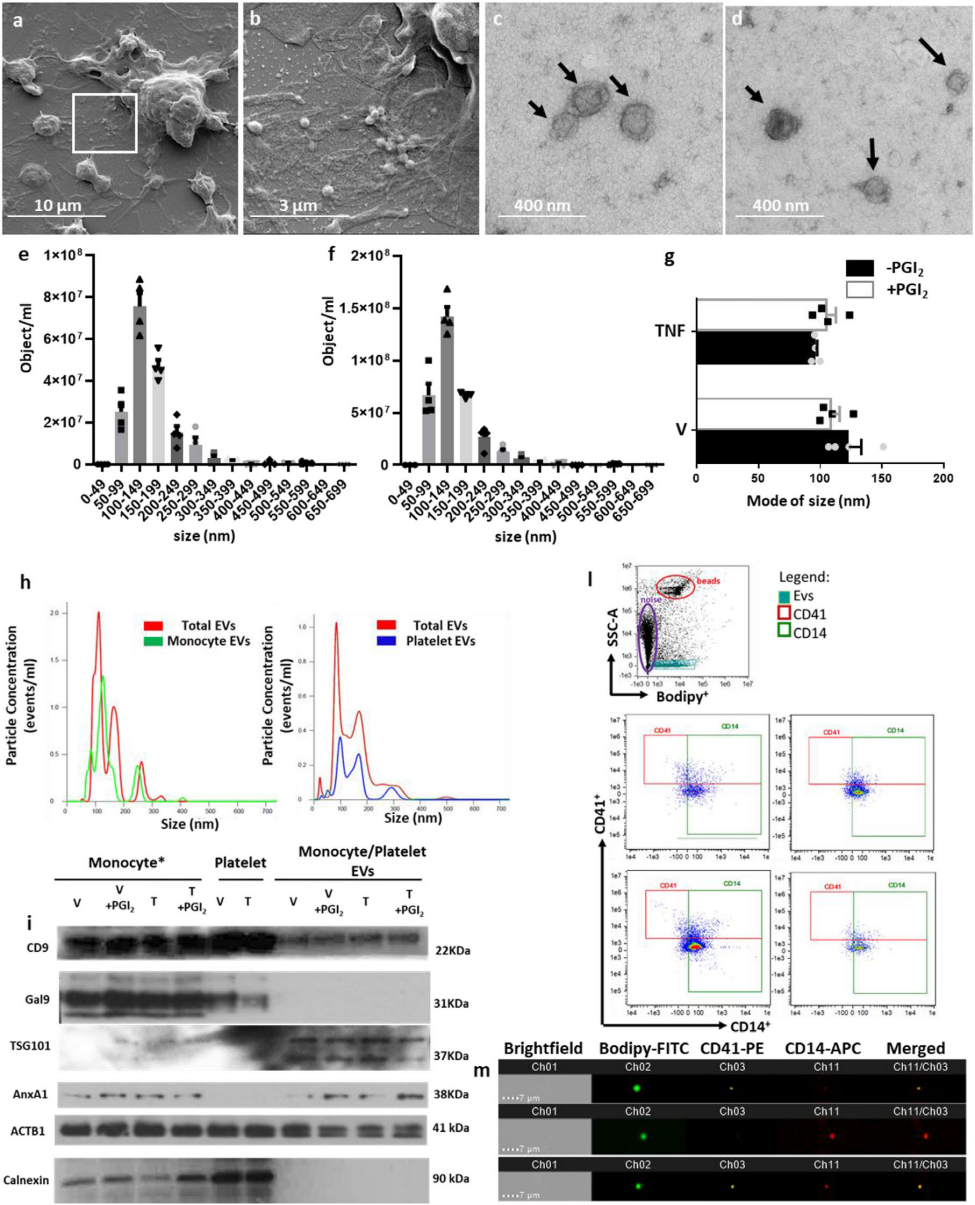
**Full characterisation of monocyte/platelet EVs**. [EVs were recovered from monocytes prepared as in Figure 1 and incubated with vehicle (Veh) or TNF‐α (50 ng/ml), in the presence or absence of Iloprost (2 μM; PGI_2_) for 60 min. (a) Representative SEM images of an activated monocyte/platelet aggregate. EVs released from a monocyte/platelet aggregate highlighted in the white box and visualized by SEM. (b) Magnification of EV release from monocyte/platelet aggregates. (c) Representative images of EVs released monocyte/platelet aggregates stimulated with PBS, (d) monocyte/platelet aggregates stimulated with TNF‐α.(e‐f) Nanoparticle Tracker Analysis (Nanosight). (e) EVs from vehicle‐incubated cells. (f) EVs from TNF‐α‐stimulated cells. (g) Mode of diameter of the different EV subsets isolated from TNF‐α‐stimulated cells and unstimulated cells in presence or absence of PGI_2_. Data are mean ± SEM of four distinct EV preparations from different donor cells. (h) Comparison of CD14‐AF‐488 and CD41‐AF‐488 stained EVs and unstained EVs of the same sample by Nanoparticle tracking analysis. (i) Western blot analysis for Galectin‐9 (Gal9) and Calnexin used as negative control for EVs, TSG101 and CD9 used as positive control for EVs, whereas Annexin A1 (AnxA1) was tested as positive control in both monocyte and monocyte EVs. Actinomyosin light chain B (ACTB1) was used as loading control. (l) ImageStream analysis of the vesicle showing quadrant selections, and representative images following staining for anti‐CD14 or anti‐CD41 of EVs isolated from monocyte preparation incubated with TNF‐α. (m) Visualization of CD41^+^ (top panel), CD14^+^ (middle panel) and CD14^+^/CD41^+^ double positive (bottom panel) EVs by ImageStream^x^. *Monocyte refers to samples containing monocyte/platelet aggregates; V: EVs from monocyte/platelet aggregates stimulated with Vehicle, V + PGI_2_: EVs from monocyte/platelet aggregates stimulated with Vehicle and PGI_2_, T: EVs from monocyte/platelet aggregates stimulated with TNF‐α, T + PGI_2_: EVs from monocyte/platelet aggregates stimulated with TNF‐α and PGI_2_]

Prior to testing the functional activity of these EVs, their physical characteristics were studied by nanoparticle tracking analysis, Western blot analysis as well as scanning and transmission electron microscopy (SEM, TEM). SEM analysis confirmed aggregation of monocytes and platelets (Figure 3a) and the release of EVs upon stimulation with TNF‐α (Figure 3b). TEM revealed a similar distribution of size for the EVs isolated by monocyte and platelet aggregates (Figure 3c). No particular difference in size could be observed in EV released by monocyte/platelet aggregates upon application of different stimuli (Figure 3c‐d and Figure S3).

Experiments performed by nanoparticle tracking analysis confirmed that vesicles produced in these settings ranged between 50  and 500 nm in diameter (Figure 3e‐f) with a similar mode (around 108 nm) (Figure 3g). Furthermore, Nanoparticle tracking analysis performed on labelled EVs confirmed expression of CD14 on the majority of them, while a smaller proportion were found to express CD41 (Figure 3h). These data are in line with the IS^x^ analysis (Figure 3l and 3m).

To check the quality and the purity of the EV preparations, Western blot analysis of the different subsets of isolated EVs were performed side‐by‐side with both platelets and monocyte/platelet aggregate extracts (Figure 3i). Here, the ISEV approved Calnexin as well as Galectin‐9 (Gal‐9) were used as a negative control. The latter was chosen since, as described later, it was not identified in our proteomics results. TSG101 was used as a known EV marker and AnxA1, a recognized marker for both monocytes and membrane‐spawning EVs. As expected, results showed EVs do not contain any Gal‐9 or Calnexin, but were positive for CD9, AnxA1 and TSG101 (Figure 3i). On the other hand, CD9, Gal‐9 and AnxA1 were detected in monocyte/platelet aggregates, while platelets seem to express only Gal‐9 and CD9 (Figure 3i). Moreover, none of the cell types were enriched in TSG101, the EV marker (Figure 3i). These data suggest no contamination in the EV preparation due to the isolation method.

### 4.2 EVs differentially activate HUVEC and HAoEC

**FIGURE 4:**
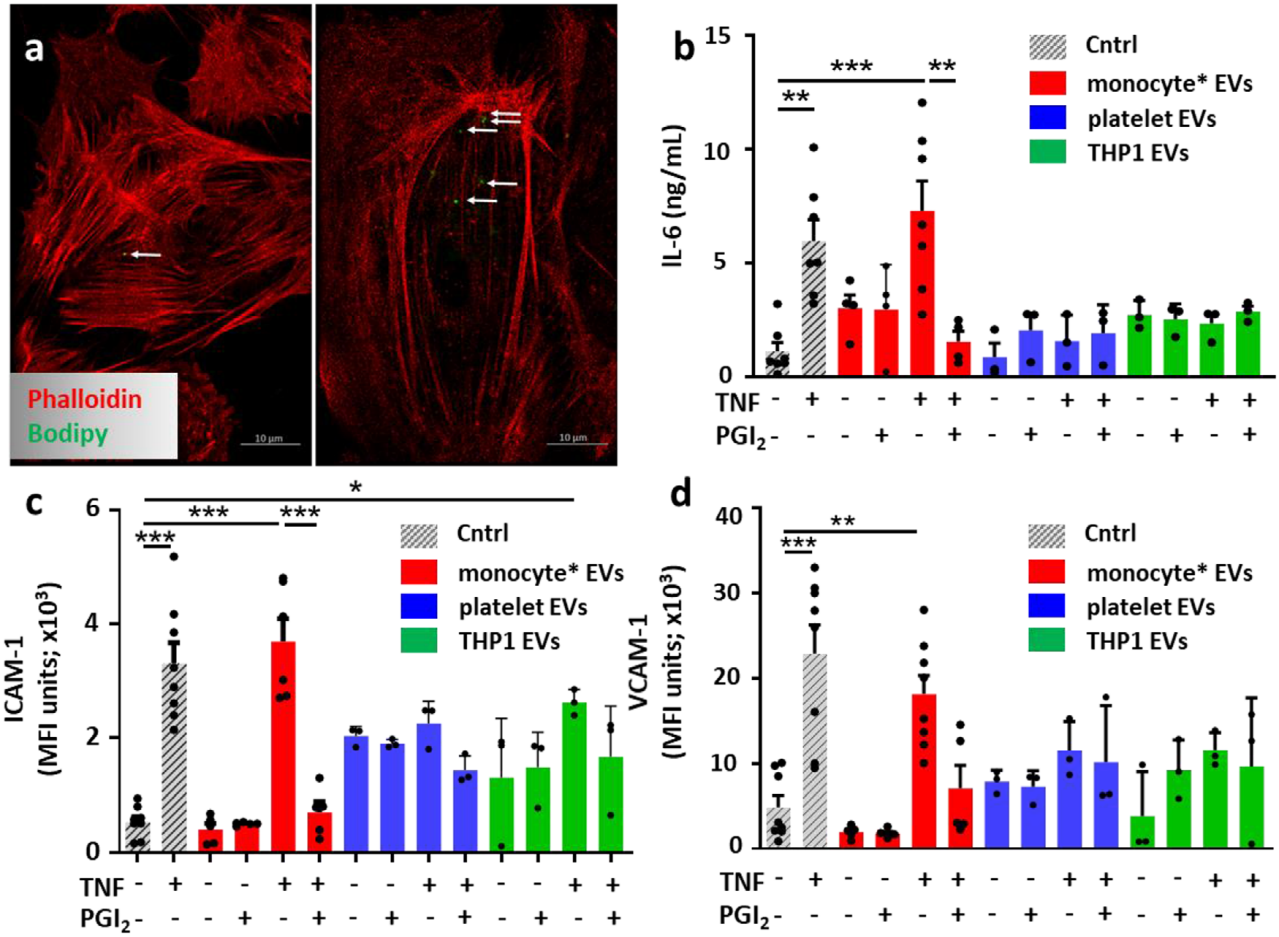
**Monocyte/platelet EVs activate HUVEC in vitro**. [EVs were collected from isolated monocyte/platelet aggregates, isolated platelets or THP1 cells incubated with vehicle (V) or TNF‐α (50 ng/ml) for 60 min, in presence or absence of Iloprost (2 μM; PGI_2_) for 60 min. HUVEC were incubated with the different EV sets (10 × 10^6^/ml) overnight. Cells were stained for flow cytomentry analysis and supernatants collected and analysed for cytokine release. (a) Confocal images of the uptake by HUVEC stained with Phalloidin (red) after 24 h of BODIPY labelled EV isolated from TNF‐α stimulated cells (white arrows, left panel) or from unstimulated cells (white arrow, right panel). (b) IL‐6 levels by ELISA. (c‐d) Quantification of ICAM‐1 and VCAM‐1 expression (MFI units) in HUVEC treated with different subsets of monocyte/platelet derived EVs, platelet‐derived EVs, THP1‐derived EVs. (*P < 0.05, **P < 0.01, ***P < 0.001; one‐way ANOVA post Bonferroni test, mean ± SEM of n = 3–6 cell preparation incubated with distinct EV preparations from different donor cells)]

Since EVs from different cellular sources can activate endothelial cells (Kuravi et al., 2019; Wang et al., 2011), a major cellular player in blood vessel angiogenesis and plaque formation (Aharon et al., 2008; Dalvi et al., 2017), we queried whether EVs derived from monocyte/platelet aggregates could impact HUVEC reactivity. An overnight protocol was applied, testing initially a concentration‐range of 1 to 20 EVs per endothelial cell (data not shown). These experiments, combined with published data (Tang et al., 2016; Wang et al., 2011), indicated that a ratio of 10 EVs/Cell was optimal for our experimental approach. Indeed, microscopy imaging showed a significant uptake of these EVs by cells after 24 h incubation with TNF‐α (Figure 4a).

We next measured IL‐6 release, a pivotal cytokine associated with atherosclerosis (Libby & Rocha, 2018). Cell incubation with EVs released by TNF‐α stimulated monocyte/platelet aggregates augmented significant IL‐6 levels to a similar extent as the positive control recombinant TNF‐α (10 ng/ml) (Figure 4b). When EVs were produced in the presence of Iloprost, a significantly lower amount was quantified (Figure 4b). Furthermore, no significant IL‐6 release was observed when HUVEC were stimulated with EV subsets isolated from either platelets or the monocytic cell line (THP‐1; utilised as a surrogate for pure monocytes with no platelets present). These data suggest a distinct pro‐inflammatory effect of EVs generated from enriched monocyte/platelet preparations in response to conditions chosen to mimic vascular inflammation.

Next, we quantified markers of endothelial cell activation; ICAM‐1 and VCAM‐1, which were significantly upregulated in response to recombinant TNF‐α (10 ng/ml) used as a positive control (Figure 4c‐d). Similar increases were recorded when HUVEC were stimulated with EVs isolated from TNF‐α activated monocyte/platelet preparations (Figure 4c‐d). Of note, only negligible amounts of residual TNF‐α were detected in any of the vesicle preparations used (110.65±10.52 fg/m; n = ‐4; data not shown). When EVs were generated in the presence of TNF‐α+Iloprost, more modest responses were observed, with minimal changes in ICAM‐1 and VCAM‐1 expression (Figure 4c‐d). When HUVEC were treated with the same concentrations (10 × 10^6^) of washed‐platelet EVs or THP‐1 derived EVs, levels of ICAM‐1 and VCAM‐1 were modulated to a much lower extent; only EVs from TNF‐α‐activated THP‐1 cells were able to significantly increase ICAM‐1 expression (Figure 4c‐d). These data suggest a synergistic role of monocyte/platelet aggregates in releasing functional EVs upon TNF‐α stimulation (Figure 4c‐d).

Comparable results were obtained when monocyte/platelet derived EVs were added to HAoEC. In this set of experiments, a significant increase in both ICAM‐1 and VCAM‐1 cell‐surface expression was quantified only when cells were treated either with 10 ng/ml of TNF‐α or with EVs isolated from monocyte and platelet aggregates stimulated with TNF‐ α, in absence of Iloprost (Figure S4). The same held true for the release of IL‐6 (Figure S4).

### 4.3 EVs trigger differential activation of human atherosclerotic plaque

**FIGURE 5:**
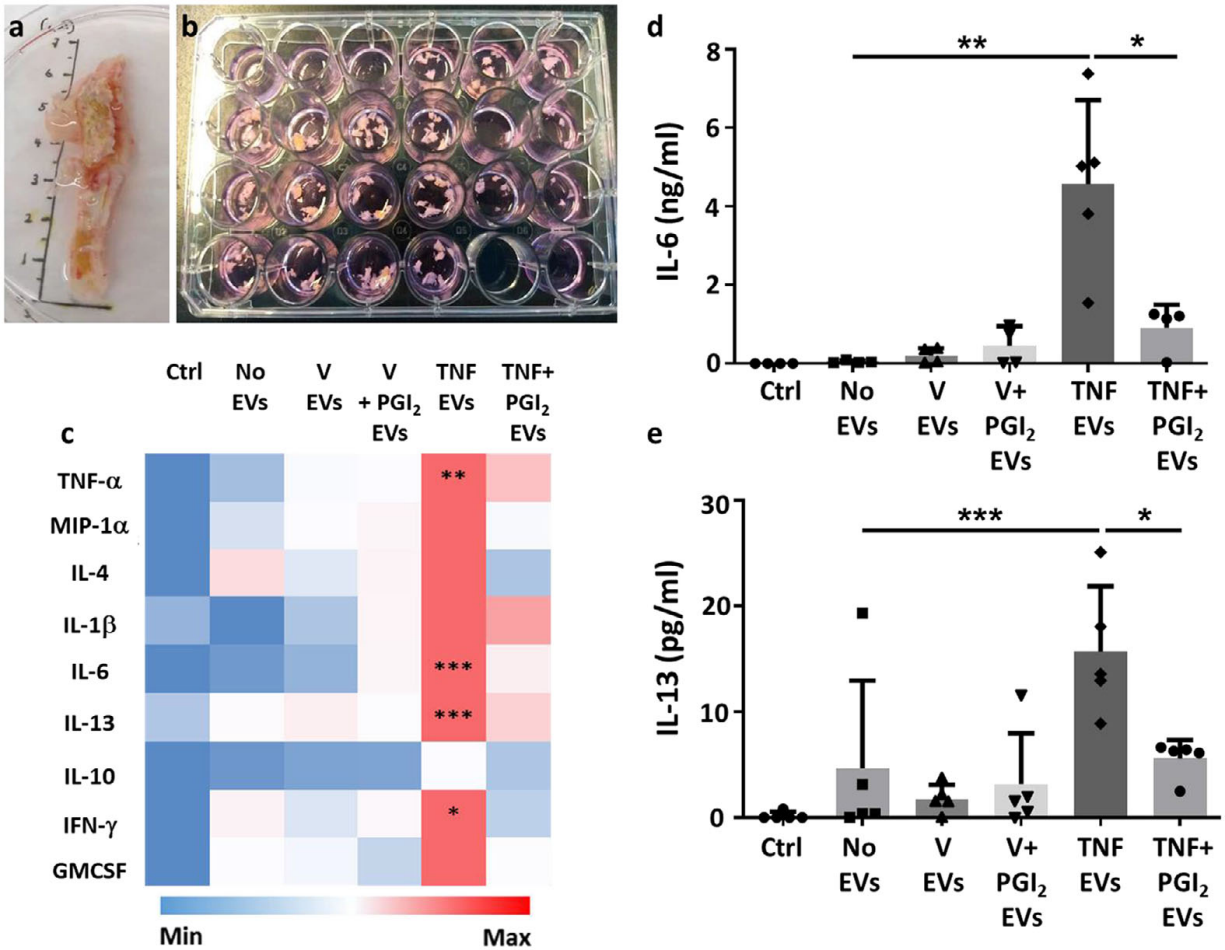
**Monocyte/platelet EVs activate human atherosclerotic plaque ex‐vivo. [**Monocyte were obtained as in Figure 2 and incubated with vehicle (V) or TNF‐α (50 ng/ml), in presence or absence of Iloprost (2 μM; PGI_2_) for 60 min. Human atherosclerotic plaque fragments were incubated with the reported EVs (10 × 10^6^/ml) overnight. Supernatants were collected and used for ELISA analysis. (a, b) Representative images of human femoral plaque and fragment incubation. (c) Heat map analysis showing qualitative modulation of cytokine release (linear scale bar). (d, e) Quantification of IL‐6 and IL‐13 levels by ELISA. (*P < 0.05, **P < 0.01, ***P < 0.001; one‐way ANOVA post Bonferroni test, mean ± SEM of n = 5 plaques incubated with distinct EV preparations from different donor cells)]

Having confirmed that EVs derived from monocyte/platelet aggregates can activate endothelial cells, we next tested if they might be a functional determinant in atherosclerosis. Thus, we assessed their function on an atherosclerotic plaque using an *ex‐vivo* organ culture protocol (Figure 5a and b; Table S1). Herein we compared an overnight incubation with EVs generated from different cellular activation protocols, using the same concentration of EVs, as described in the previous section, to mimic settings of vascular inflammation. Then, we quantified cytokines and proteins released in the supernatants from the plaque fragments.

Cytokine multiplex analyses revealed that treatment of the plaques with EVs released by monocyte/platelets stimulated with TNF‐α, augmented concentrations of TNF‐α, IL‐6, IL‐13, IFN‐γ and GM‐CSF in the culture media (Figure 5c and Supplementary Table S2). As stated above, negligible amounts of residual TNF‐α were detected in any of the vesicle preparations used. When EVs were generated in the presence of Iloprost, a much milder regulation of the general cytokine response was noted (Figure 5c). These findings confirm the acquisition of a pro‐inflammatory phenotype of EVs not only in vitro but also ex vivo when monocyte/platelet preparations were stimulated with TNF‐α. Such an effect was markedly attenuated when EVs were generated by Iloprost+TNF‐α treatment, a finding corroborated by further quantification of IL‐6 and IL‐13 in the supernatants (Figure 5d and e). Of importance, the use of 0.1% FBS in the culture media to enable the viability of plaque fragments did not affect the experimental outcome, as showed by the cytokines release, where basal levels were significant lower than when plaques where treated with the subsets of EVs.

**FIGURE 6:**
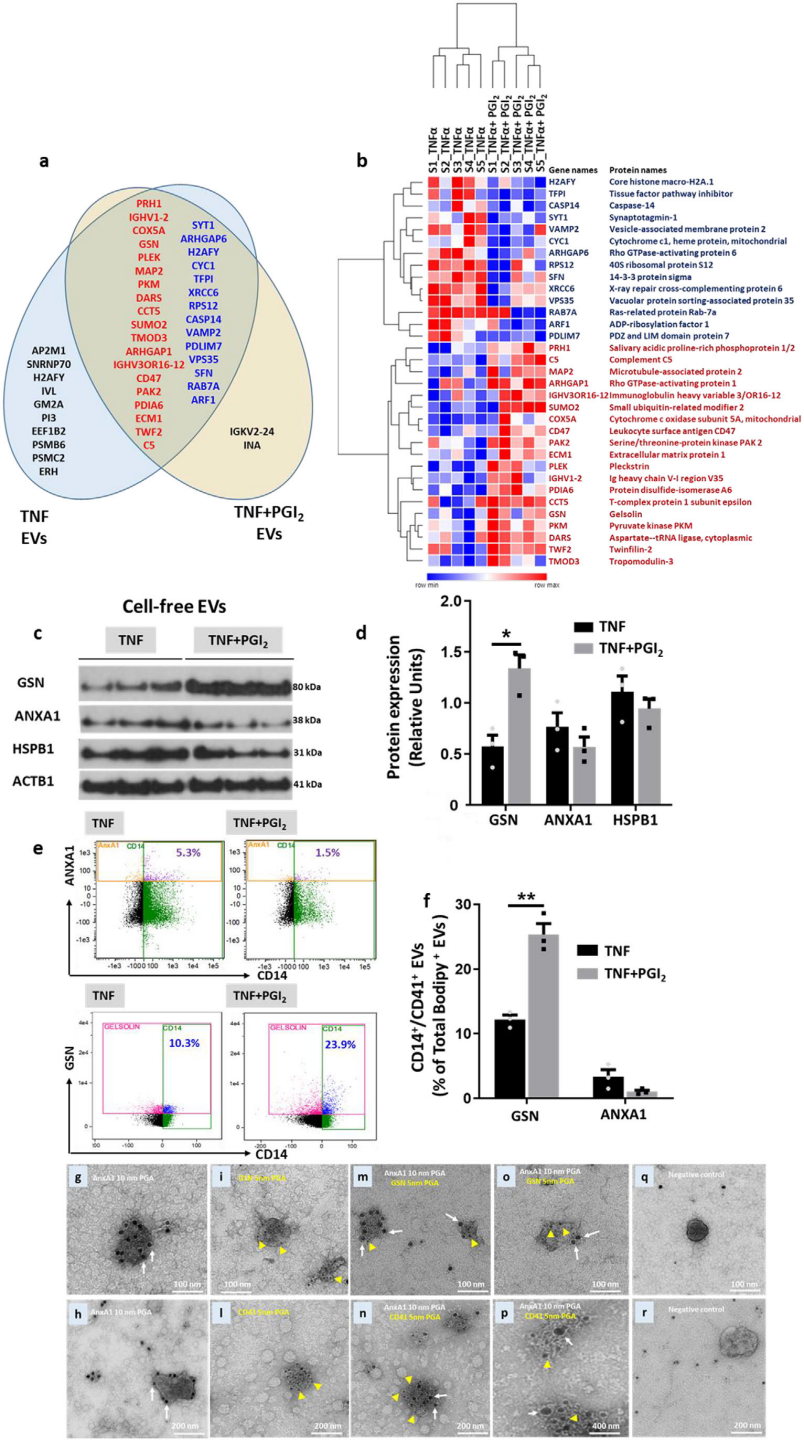
**Proteomic analysis of monocyte/platelet EVs and validation**. [Monocyte were obtained as in Figure 2 and incubated with TNF‐α (50 ng/ml), in the presence or absence of Iloprost (2 μM; PGI_2_) for 60 min, prior to EV purification. Targeted analysis highlighting differences between TNF‐α and TNF‐α+PGI_2_ EVs identified 33 proteins that were significantly altered (Table S3). (a) Venn diagram showing the proteins that are differentially expressed between TNF‐α versus PGI2+TNF‐α EVs. In the intersection of the diagram are reported the proteins there are significantly altered between the two EVs populations (*P* < 0.05, red represents up‐regulated proteins while blue depicts down‐regulated proteins in TNF‐α+ PGI2 EVs). In black, we identify proteins that are uniquely expressed in either EVs population. (b) Hierarchical clustering heatmap of differentially expressed proteins (centre of Venn diagram in panel a) between TNF‐α versus PGI2+TNFα EVs (*P* < 0.05). (c) Western blot analyses of distinct EV preparations used to detect immunoreactivity for Gelsolin (GSN), Annexin A1 (AnxA1), heat shock protein β1 (HSPB1) and β‐actin (ACTB1). Three distinct EV preparations were tested. (d) Densitometry analysis, ACTB1 was used as loading control. (e) ISx analysis of a select group of proteins identified in the proteomic screen (see Methods for details). (f) Expression levels of GSN and ANXA1 in CD14^+^/CD41^–^ EVs. *P < 0.05, **P < 0.01, ***P < 0.001; Mann Whitney test, mean ± SEM, n = 3 distinct EV preparations. Monocyte EVs isolated from monocytes/platelet aggregates purified by RosetteSep kit and stimulated with TNF‐α (50 ng/ml). Transmission electron micrographs of double labelled EVs. (g, h) Annexin A1 (AnxA1)‐gold labelled EVs with 10 nm Protein Gold A (PGA; white arrow). (i) Gelsolin (GSN)‐gold labelled EVs with 5 nm PGA (yellow arrowhead). (l) CD41‐gold labelled EVs with 5 nm PGA (yellow arrowhead). (m, n) AnxA1‐gold labelled EVs with 10 nm PGA (white arrow), GSN‐gold labelled EVs with 5 nm PGE (yellow arrowhead). (o, p) AnxA1‐gold labelled EVs with 10 nm PGA (white arrow). Electron micrograph of EVs treated with mixed PGA only (q, r)]

Characterization of monocyte EV subsets revealed differential protein expression associated with regulation of vascular inflammation and plaque formation. The experimental data presented so far are indicative of different pharmacodynamic properties of EVs obtained with TNF‐α‐treated monocyte/platelet preparations as compared to vesicles generated following treatment with Iloprost+TNF‐α. In order to verify if these effects were mediated by differences in EV composition, we performed a proteomic characterization of TNF‐α and Iloprost+TNF‐α EVs. This set of experiments identified 681 proteins in EVs by LS‐MS/MS (Table S3), of which 32 proteins were significantly altered (*P*  <  0.05) when comparing TNF‐α EVs to Iloprost+TNF‐α EVs (Figure 6a). Of these, 19 proteins were upregulated and 13 downregulated following cell incubation with Iloprost (Figure 6b). Moreover, proteins uniquely expressed were also identified: 10 proteins for TNF‐α EVs and only two for Iloprost+TNF‐α EVs (Figure 6a). Of interest, we detected AnxA1, which is a faithful marker for membrane‐spawn vesicles (Jeppesen et al., 2019). Gelsolin (GSN), which was augmented in Iloprost+TNF‐α EVs, was an interesting hit as it is involved in actin filament assembly and organization (Sun et al., 1999), hence described to maintain the cytoskeleton structure in arteries (see Discussion).

Next, and to further validate these data, we confirmed the relative abundance of a selected group of proteins by Western blotting and IS^x^. To this end, equal numbers of monocyte/platelet EVs of each subset were loaded and immunostained for GSN, AnxA1 and HSPB1 employing ATCB as a loading control (Figure 6c). The blots confirmed that GSN was enriched in Iloprost+TNF‐α EVs (Figure 6c‐d), whereas HSPB1 and AnxA1 were mildly regulated across the two EV subsets (Figure 6c‐d). IS^x^ analyses revealed that GSN and AnxA1 were also detected of the surface of the EVs (Figure 6e), visualizing a selective enrichment of GSN in EVs isolated from monocytes stimulated with Iloprost and TNF‐α (Figure 6f), with no major changes for AnxA1 surface detection.

When immunogold labelling experiments with anti‐AnxA1, GSN or CD41 antibodies were analyzed by TEM, EVs expressing both markers could be identified (Figure 6g‐r). These EVs were heterogeneous in their size, with both larger as well as smaller particles positive for the markers.

**FIGURE 7:**
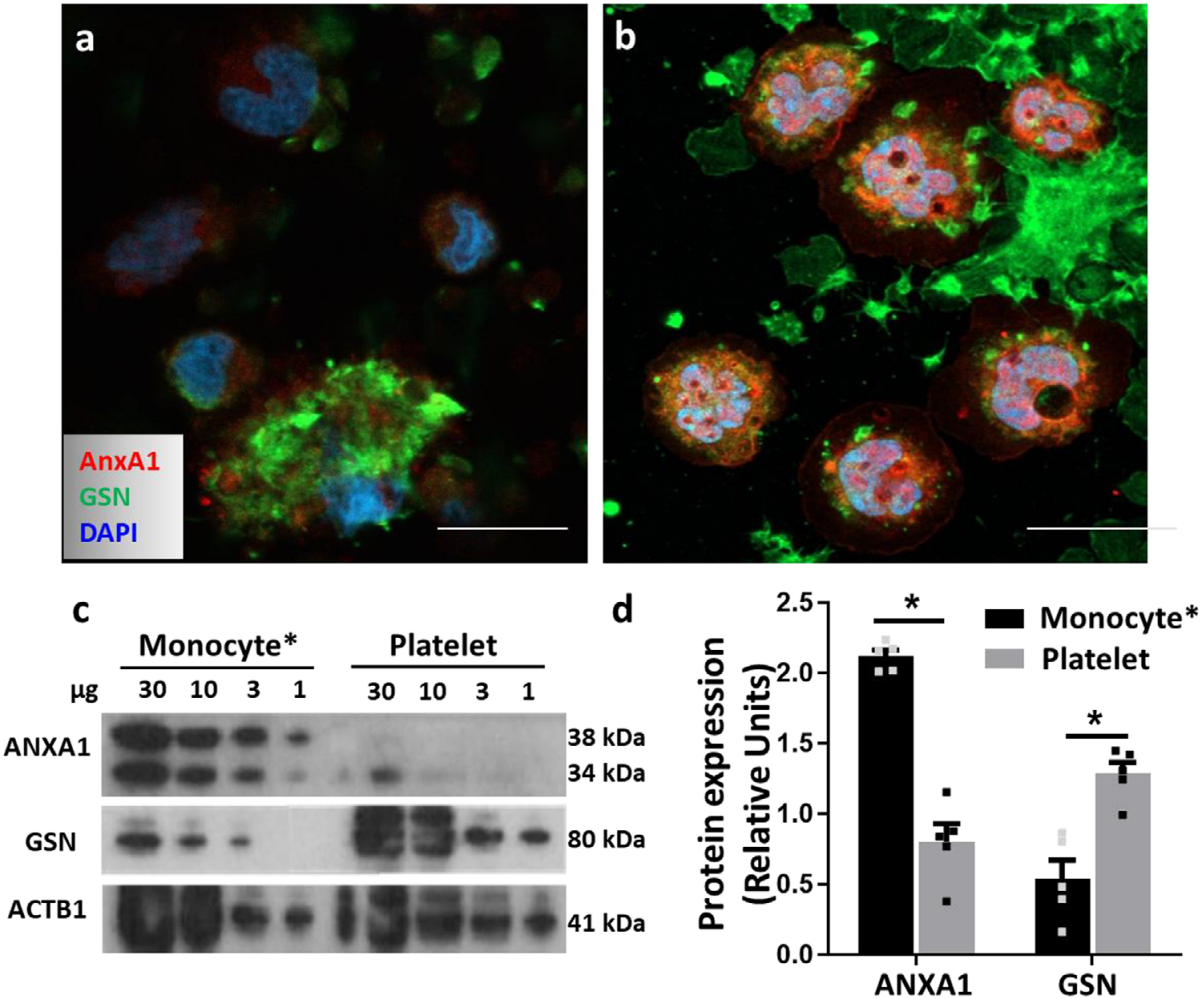
**Selective expression of Annexin A1 and Gelsolin in human monocytes and platelets**. [The monocyte/platelet preparation was obtained as in Figure 2. Cells were permeabilised or left intact prior to staining for Annexin A1 (ANXA1) and Gelsolin (GSN), prior to imaging. (a) Surface staining for ANXA1 (red) and GSN (green) in monocyte/ platelet aggregates. (b) Intracellular staining for ANXA1 (red) and GSN (green) in monocyte/ platelet aggregates. DAPI counterstaining (blue) indicates cell nuclei. Representative of three distinct cell preparations. Scale bar = 10‐μm. (c) Western blot analysis for ANXA1 and GSN in monocyte and platelet lysates. (d) Densitometry analysis. ACTB1 was used as loading control. (*P < 0.05, **P < 0.01, ***P < 0.001, Mann Whitney test, mean ± SEM of n = 5 with different donor cells). *Monocyte refers to samples containing monocyte/platelet aggregates isolated with RosetteSep kit]

To determine the cellular source of these exemplar proteins, surface and intracellular staining of human monocyte and platelet aggregates was performed and analyzed by microscopy. While AnxA1 was selectively expressed, and to a high abundance, by monocytes (Figure 7a and b), the majority of GSN seemed expressed by platelets both intracellularly and on their surface (Figure 7b); only a small amount was associated to monocytes likely because of the adherent platelets. Similar results were also obtained by Western blotting when the same proteins were investigated in platelet or monocyte (the latter containing residual platelets) lysates. Loading of decreasing concentrations of monocyte and platelet whole lysates revealed GSN to be highly expressed in platelets; in line with the immunofluorescence results, only a small amount was detected in monocyte lysates, possibly because of platelet contamination (Figure 7c). Conversely, monocyte lysates contained a consistently higher amount of AnxA1 with the characteristic 38 kDa and 34 kDa bands (Figure 7c). Platelet extracts displayed only minimal amounts of the cleaved form of AnxA1 (Figure 7d). These data, together with the immuno‐gold labelling TEM results, confirmed the ability of monocyte/platelet aggregates to release EVs bearing both markers of monocyte and platelet origin.

**FIGURE 8:**
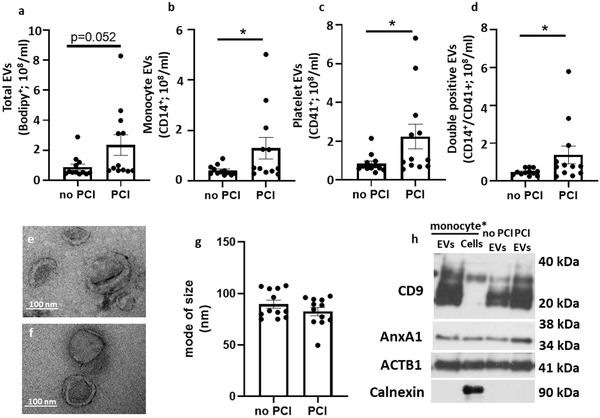
**Elevated pro‐inflammatory monocyte/platelet EVs in patients with coronary artery disease**. [EV were isolated with differential centrifugation from plasma samples, re‐suspended in PBS and analysed on ImageStream, Nanosight, TEM and Western blot. (a) Concentrations of CD14^+^ EVs (b), CD41^+^ EVs (c) and CD14^+^/CD41^+^ EVs were analysed in samples by case status. Representative TEM images of EVs analysed by electron microscopy from patient not in need of percutaneous coronary intervention (e) and that needed the surgery (f). (g) mode of size of EVs. Western blot analyses from monocyte/platelet aggregates and of distinct EV preparations from monocyte/platelet EVs (used as internal control) and from patients, used to detect immunoreactivity for CD9, Annexin A1 (AnxA1), β‐actin (ACTB1) and Calnexin. Three distinct EV preparations were tested. Data are from N = 12 patient samples per group and are reported as median ± SEM (*P < 0.05, **P < 0.01, ***P < 0.001; Unpaired Student's *t*‐test). *Monocyte refers to samples containing monocyte/platelet aggregates]

Having established that EVs from monocyte and platelet aggregates are heterogeneous and could promote inflammation in the context of atherosclerosis, we next assessed if similar EV subsets could be detected in patients with atherosclerotic plaques. Herein we analyzed a cohort of 24 patients with coronary artery disease (CAD), 12 of whom required percutaneous coronary intervention (PCI). Baseline characteristic of the patients are reported in Table S5. A significant increase in overall concentrations of EVs (Bodipy^+^; Figure 8a) as well EVs from monocytes (CD14^+^/CD41^–^; Figure 8b), platelets (CD14^–^/CD41^+^; Figure 8c) and double positive (CD14^+^/CD41^+^ EVs; Figure 8d) was quantified in the plasma of CAD patients who needed stenting. No difference in size was observed between the two groups of patients when EVs were analyzed both by TEM (Figure 8e‐f) and Nanoparticle tracking analysis (Figure 8g). The nature of the EVs was confirmed by Western blot, where EVs from patients expressed EV markers like CD9 and AnxA1 and did not show contamination of Calnexin (Figure 8h). In the same blot, EVs from monocyte/platelet aggregates were loaded as positive control (Figure 8h).

## 5 DISCUSSION

In this study we provide evidence that monocyte/platelet‐derived EVs are pro‐inflammatory and activate endothelial cells and the human atherosclerotic plaque. We identify some subtlety in relation to the mode of activation of the monocyte with particular attention to the presence of an aggregated and/or adherent platelet. Using a pharmacological approach to attenuate platelet reactivity, we could produce EVs with a lower impact on atherosclerotic plaque activation. These different outcomes were not related to physicochemical features of the EVs but rather to their composition as indicated by the proteomic analysis. Similar EVs to the one investigated, bearing markers of both monocytes and platelets, were increased in patients suffering from carotid artery disease needing percutaneous angiography. Since transient aggregates between monocytes and platelets can form in settings of vascular inflammation, these data make us propose that this inter‐cellular cross‐talk can generate EVs which may extend the patho‐physiological relevance of this event. Clinical management with anti‐platelet therapies may have additional beneficial effects through modulation of the quality of EVs released from monocytes. Furthermore, these EV subsets could be exploited as potential biomarkers of worsening of atherosclerosis.

Monocyte/platelet aggregates are a feature of vascular inflammation, being identified in several settings, both in man and experimental animals. For instance, following kidney transplantation, addition of a 4‐week anti‐platelet therapy to immunosuppressive drugs reduced monocyte/platelet aggregates as well as other markers of vascular inflammation (Graff et al., 2005). These aggregates have also been reported in stroke (Franks et al., 2010) and in heart failure (Wrigley et al., 2013). In heart failure, presence of monocyte/platelet aggregates negatively correlated with better prognosis, indicating a role for the aggregates in sustaining damage of the cardiac tissue. Finally, circulating monocyte/platelet aggregates have been detected in hypertension, where an independent predictor for their formation was systemic blood pressure (Gkaliagkousi et al., 2009), and in coronary artery disease. In the latter condition, monocyte/platelet aggregates increase in patients compared to healthy controls (Czepluch et al., 2014; Sarma et al., 2002), an increase quantified to be more than two‐fold (Furman et al., 1998). In all these studies, the pro‐atherogenic properties of the aggregates has been suggested.

It is important to note that monocyte/platelet aggregates are transient in their association and dissociation. As a relevant example, Furman *et al* demonstrated that numbers of circulating monocyte/platelet aggregates in patients with acute myocardial dysfunction were higher within the first 4 h of acute coronary symptoms and gradually returned to basal values in the 4–8 h post‐infarct period (Furman et al., 2001). In view of the transient nature of the cellular aggregates, we reasoned that EVs could represent a viable way to monitor longer‐term effects of aggregate formation, either as a biomarker or as *bona fide* effectors of pathogenesis. To address this hypothesis, we took advantage of the presence of platelets in the preparations of monocytes purified from human whole blood.

A subtle and sophisticated role for the platelet emerged in these experimental conditions, whereby attenuation of platelet activation (virtually with all three therapies tested) only partially affected platelet adhesion to the monocyte, while reducing the generation of EVs, including the CD14^+^/CD41^+^ double positive subset. Here, we had a system where TNF‐α stimulated the monocyte predominantly, but there was a ‘co‐stimulatory’ action attained by the adherent platelets. These data are in agreement with a proposed cross‐talk in plaque formation and progression, whereby the platelet adherent to the monocyte favours migration of the leukocyte into the plaque, which would then develop to macrophages (Da Costa Martins et al., 2004; Huo et al., 2003; Mayr et al., 2009). Platelet‐delivery of cholesterol could feed forward the process of macrophage differentiation into foam cells (Badrnya et al., 2014). Here we reasoned that one downstream result of platelet/monocyte aggregate formation would be production of pro‐inflammatory EVs.

The genuine nature of the EVs was confirmed through multiple experimental approaches, including Nanosight and electron microscopy analyses, which revealed a similar size of these structures, unaltered by cell exposure to TNF‐α or the prostacyclin analogue. In all cases, an average diameter of > 100 nm was quantified suggesting that EVs produced are predominantly formed by membrane‐spawn vesicles and not exosomes (Jeppesen et al., 2019). This evidence was corroborated by the proteomic analysis and further validated by Western blot, which identified AnxA1 in all subsets of EVs: this protein is a genuine marker for membrane‐derived vesicles (Jeppesen et al., 2019). More interesting to us is the emerging evidence that the same cell can generate EVs which are at least in part different in relation to the stimulus applied or the microenvironment, with previous work focusing on neutrophil‐derived EVs (Dalli et al., 2013, 2014). Here, we observed that monocyte‐derived EVs isolated from mixed platelet/monocyte aggregates in inflammatory conditions (TNF‐α) bind to and are internalized by HUVECs. Before exploring further the differences in composition we further tested the potential different effector functions of TNF‐α and Iloprost+TNF‐α EVs in more complex settings, using an organ culture protocol of the atherosclerotic plaque.

There has been quite some interest in EVs and atherosclerosis, mainly with a focus on vesicles released from the plaque, possibly as a downstream determinant of pathogenic processes operative within the diseased vessel. Several studies showed that EVs are mainly derived from leukocytes and i) are endowed with thrombogenic activities (Leroyer et al., 2007), ii) can increase intra‐plaque neovascularization and plaque vulnerability, iii) enhance proliferation of endothelial cells and angiogenesis (Leroyer et al., 2008) through the presence of tissue factor activity (Morel et al., 2006). The leukocyte origin was further confirmed by Mayr *et al*., who identified the myeloid EV fraction, as well as EVs from smooth muscle cells and erythrocytes. Metabolomics analyses showed an increase in taurine, expression of a monocyte‐produced oxidative microenvironment within the atherosclerotic plaque (Mayr et al., 2009). Here we revealed marked modulatory functions of monocyte EVs applied to the plaque with selective changes in specific cytokines. Indeed, the increase in IL‐6 is remarkable and agrees with the importance of this cytokine in atherosclerosis (Libby & Rocha, 2018). Exogenously administered IL‐6 enhances development of fatty lesions in mice (Huber et al., 1999), while in man this cytokine is enhances endothelial dysfunction and aortic stiffness: rheumatoid arthritis patients treated with anti‐IL‐6 therapy displayed reduced articular inflammation and decreased endothelial dysfunction (Protogerou et al., 2011). A recent Mendelian randomized study, focusing on the single nucleotide polymorphisms in the IL‐6 receptor gene, highlighted loss of function as a viable approach for the prevention of coronary heart disease (Swerdlow et al., 2012). It was of great interest to us that the vesicles generated by the monocyte preparation stimulated with Iloprost+TNF‐α displayed a different impact on the plaque, with a blunted cytokine response. We obtained evidence for a broader alteration of the proteome released from the plaque (list of hits deposited under the name PRIDE PXD014325 (http://www.ebi.ac.uk/pride/archive/projects/ PXD014325).

Collectively, these experiments justified an in‐depth analysis of the potential differences between TNF‐α EVs and Iloprost+TNF‐α EVs. While we recognize that structural lipids, lipid mediator precursors (Norling et al., 2011), microRNA and other nucleic acids (Guduric‐Fuchs et al., 2012) could vary between the two vesicle type, we analyzed their protein contents. In general a lower number of significantly enriched proteins were detected in Iloprost+TNF‐α EVs compared to TNF‐α EVs suggesting that attenuation of platelet activation not only reduced the number of CD14^+^/CD41^–^ and CD14^+^/CD41^+^ EVs, but also modified their actual composition. Addition of Iloprost reduced the number of proteins exclusively identified in monocyte EVs from 10 to 2. Comparison with published proteomic lists revealed interesting overlaps (Table S4). As an example, THP‐1 monocytic cells stimulated with lipopolysaccharide yield EVs that contain EEF1B2 (an elongation factor) and PSMC2 (proteasome subunit) (Bernimoulin et al., 2009), two of the proteins uniquely identified here in TNFα EVs. Out of six published studies on platelet EVs, we focused on the two where similar preparation protocols were used (Aatonen et al., 2014; Pienimaeki‐Roemer et al., 2015): INA (cytoskeleton component) uniquely identified in Iloprost+TNF‐α EVs was identified by Pienimaeki‐Roemer *et al*. in senescent platelet EVs (Pienimaeki‐Roemer et al., 2015). In our hands, Iloprost+TNF‐α EVs also express PSMC2 as well as AP2M1 (vesicle transporter) and PSMB6 (another proteasome subunit) (Pienimaeki‐Roemer et al., 2015). An interesting hit was gelsolin (GSN), more abundant in Iloprost+TNF‐α EVs: this protein is anti‐inflammatory and detected in resolving inflammatory exudates (Kaneva et al., 2017). Published proteomic analyses have reported gelsolin downregulation in atherosclerotic compared to pre‐atherosclerotic coronary arteries (De La Cuesta et al., 2012). A reduction in gelsolin levels can deregulate the cytoskeleton within the human atherosclerotic coronary media layer and switch medial vascular smooth muscle cells from a contractile to a pro‐inflammatory synthetic phenotype (De La Cuesta et al., 2013). Furthermore, circulating levels of gelsolin are reduced in patients with a diagnosis of asymptomatic carotid artery plaque (Bhosale et al., 2018). The translational relevance of these new findings derives from the detection of monocyte and platelet EVs, as well as EVs bearing both markers, in CAD patients. Importantly, their numbers were elevated when patients had confirmed atherosclerotic plaque and required surgical intervention.

The role of EVs in atherosclerosis, especially platelet EVs, has been already investigated and described in several reviews (Charla et al., 2020; Oggero et al., 2019; Van Der Vorst et al., 2018). However, as a relevant example, a recent publication by Zaldivia et al. reports changes in platelet‐derived EVs in patients with hypertension and renal denervation (Zaldivia et al., 2020). As such, it is relevant to mention that cardiovascular diseases others than atherosclerosis may be characterized by altered EV generation including the vesicles that derive from monocyte/platelet aggregates (Jansen et al., 2017; Zaldivia et al., 2017).

In conclusion, the activating effect of monocyte‐derived vesicles on the reactivity of the atherosclerotic plaque reflects the contribution of platelet adhesion. Monocyte/platelet aggregates, accepted as a predictive marker of several cardiovascular pathologies including coronary artery disease, may have longer lasting pathogenic effects through generation of vesicles which may propagate pro‐inflammatory actions. Modulation of platelet reactivity could help attenuate the detrimental properties of these vesicles.

## DECLARATION OF INTERESTS

None.

### AUTHOR CONTRIBUTIONS

Mauro Perretti devised the project, the main conceptual idea, planned and analyzed data and proof outline. Silvia Oggero planned the project, designed and performed experiments, analyzed the data. Catherine Godson helped supervise the project, designed experiments, analyzed the data; Dianne Cooper and Lucy V. Norling designed experiments, analyzed the data. Monica de Gaetano and Eoin P. Brennan assisted with ex vivo atherosclerotic plaque experiments and Simone Marcone helped carry out the proteomic determinations and analyses. Andreia L. Pinto and Thomas Burgoyne assisted with the transmission electron microscopy experiments and Dinara Ikramova helped running scanning electron microscopy experiments. Trinidad Montero‐Melendez helped with the interpretation of the proteomic data. Stephen Fitzsimons, David Burke, Orina Belton and Mary Barry helped providing the human samples used in the study. Silvia Oggero and Mauro Perretti wrote the manuscript, all other authors commented on it.

### FUNDING SOURCES

EVOluTION has received funding from the European Union's Horizon 2020 research and innovation programme under the Marie Sklodowska‐Curie grant agreement No. 675111 (Silvia Oggero, Mauro Perretti). The ImageStream™ used was funded by the Wellcome Trust (infrastructure grant 101604/Z/13/Z). Monica de Gaetano is supported by an IRC Government of Ireland postdoctoral fellowship (IRC GOIPD/2017/1060), Eoin P. Brennan and Catherine Godson are supported by Science Foundation Ireland grants 15/US/B3130 and 15/IA/3152 and a strategic research award from JDRF NY, USA. Lucy V. Norling is supported by a Versus Arthritis Senior Research Fellowship grant 22235. This work has been facilitated by the National Institute for Health Research Biomedical Research Centre at Barts Hospital NHS Trust.

## Supporting information

Supporting Information

## References

[jev212084-bib-0001] Aatonen, M. T. , Öhman, T. , Nyman, T. A. , Laitinen, S. , Grönholm, M. , & Siljander, P. R.‐M. (2014). Isolation and characterization of platelet‐derived extracellular vesicles. J Extracell Vesicles, 3(1), 24692. 10.3402/jev.v3.24692 PMC4125723 25147646

[jev212084-bib-0002] Aharon, A. , Tamari, T. , & Brenner, B. (2008). Monocyte‐derived microparticles and exosomes induce procoagulant and apoptotic effects on endothelial cells. Thrombosis and Haemostasis, 100(5), 878–885. 18989533 10.1160/th07-11-0691

[jev212084-bib-0003] Annaz, B. , Hing, K. A. , Kayser, M. , Buckland, T. , & Silvio, L. Di (2004). Porosity variation in hydroxyapatite and osteoblast morphology: A scanning electron microscopy study. Journal of Microscopy, 215(1), :100–110. 15230881 10.1111/j.0022-2720.2004.01354.x

[jev212084-bib-0004] Badrnya, S. , Schrottmaier, W. C. , Kral, J. B. , Yaiw, K. ‐. C. , Volf, I. , Schabbauer, G. , Söderberg‐Nauclér, C. , & Assinger, A. (2014). Platelets mediate oxidized low‐density lipoprotein–induced monocyte extravasation and foam cell formation. Arteriosclerosis, Thrombosis, and Vascular Biology, 34(3), 571–580. 10.1161/ATVBAHA.113.302919 24371083

[jev212084-bib-0005] Bernimoulin, M. , Waters, E. K. , Foy, M. , Steele, B. M. , Sullivan, M. , Falet, H. , Walsh, M. T. , Barteneva, N. , Geng, J. ‐ G. , Hartwig, J. H. , Maguire, P. B. , & Wagner, D. D. (2009). Differential stimulation of monocytic cells results in distinct populations of microparticles. Journal of Thrombosis and Haemostasis, 7(6), 1019–1028. 19548909 10.1111/j.1538-7836.2009.03434.x PMC3242443

[jev212084-bib-0006] Bhosale, S. D. , Moulder, R. , Venäläinen, M. S. , Koskinen, J. S. , Pitkänen, N. , Juonala, M. T. , Kähönen, M. A. P. , Lehtimäki, T. J. , Viikari, J. S. A. , Elo, L. L. , Goodlett, D. R. , Lahesmaa, R. , & Raitakari, O. T. (2018). Serum proteomic profiling to identify biomarkers of premature carotid atherosclerosis. Scientific Reports, 8(1), 9209. 29907817 10.1038/s41598-018-27265-9 PMC6003912

[jev212084-bib-0007] Charla, E. , Mercer, J. , Maffia, P. , Nicklin, S. A. . Extracellular vesicle signalling in atherosclerosis. Cellular Signalling. 2020;75, 109751. 32860954 10.1016/j.cellsig.2020.109751 PMC7534042

[jev212084-bib-0008] Czepluch, F. S. , Kuschicke, H. , Dellas, C. , Riggert, J. , Hasenfuss, G. , & Schäfer, K. (2014). Increased proatherogenic monocyte‐platelet cross‐talk in monocyte subpopulations of patients with stable coronary artery disease. Journal of Internal Medicine, 275(2), 144–154. 24118494 10.1111/joim.12145

[jev212084-bib-0009] Da Costa Martins, P. , Van Den Berk, N. , Ulfman, L. H. , Koenderman, L. , Hordijk, P. L. , & Zwaginga, J. J. (2004). Platelet‐Monocyte Complexes Support Monocyte Adhesion to Endothelium by Enhancing Secondary Tethering and Cluster Formation. Arteriosclerosis, Thrombosis, and Vascular Biology, 24(1), 193–199. 10.1161/01.ATV.0000106320.40933.E5 14615387

[jev212084-bib-0010] Dalli, J. , Montero‐Melendez, T. , Norling, L. V. , Yin, X. , Hinds, C. , Haskard, D. , Mayr, M. , & Perretti, M. (2013). Heterogeneity in neutrophil microparticles reveals distinct proteome and functional properties. Molecular and Cellular Proteomics, 12(8), 2205–2219. 23660474 10.1074/mcp.M113.028589 PMC3734580

[jev212084-bib-0011] Dalli, J. , Norling, L. V. , Montero‐Melendez, T. , Canova, D. F. , Lashin, H. , Pavlov, A. M. , Sukhorukov, G. B. , Hinds, C. J. , & Perretti, M. (2014). Microparticle alpha‐2‐macroglobulin enhances pro‐resolving responses and promotes survival in sepsis. EMBO Molecular Medicine, 6(1), 27–42. 24357647 10.1002/emmm.201303503 PMC3936490

[jev212084-bib-0012] Dalvi, P. , Sun, B. , Tang, N. , & Pulliam, L. (2017). Immune activated monocyte exosomes alter microRNAs in brain endothelial cells and initiate an inflammatory response through the TLR4/MyD88 pathway. Scientific Reports, 7(1), 9954. 28855621 10.1038/s41598-017-10449-0 PMC5577170

[jev212084-bib-0013] De La Cuesta, F. , Barderas, M. G. , Calvo, E. , Zubiri, I. , Maroto, A. S. , Darde, V. M. , Martin‐Rojas, T. , Gil‐Dones, F. , Posada‐Ayala, M. , Tejerina, T. , Lopez, J. A. , Vivanco, F. , & Alvarez‐Llamas, G. (2012). Secretome analysis of atherosclerotic and non‐atherosclerotic arteries reveals dynamic extracellular remodeling during pathogenesis. Journal of Proteomics, 75(10), 2960–2971. 22197968 10.1016/j.jprot.2011.12.005

[jev212084-bib-0014] De La Cuesta, F. , Zubiri, I. , Maroto, A. S. , Posada, M. , Padial, L. R. , Vivanco, F. , Alvarez‐Llamas, G. , & Barderas, M. G. (2013). Deregulation of smooth muscle cell cytoskeleton within the human atherosclerotic coronary media layer. Journal of Proteomics, 82, 155–165. 23429260 10.1016/j.jprot.2013.01.032

[jev212084-bib-0015] Del Conde, I. , Nabi, F. , Tonda, Raúl , Thiagarajan, P. , López, José A. , & Kleiman, N. S. (2005). Effect of P‐selectin on phosphatidylserine exposure and surface‐dependent thrombin generation on monocytes. Arteriosclerosis, Thrombosis, and Vascular Biology, 25(5), 1065–1070. 10.1161/01.ATV.0000159094.17235.9b 15705928

[jev212084-bib-0016] Franks, Z. G. , Campbell, R. A. , Weyrich, A. S. , & Rondina, M. T. (2010). Platelet‐leukocyte interactions link inflammatory and thromboembolic events in ischemic stroke. Annals of the New York Academy of Sciences, 1207(1), 11–17. 20955420 10.1111/j.1749-6632.2010.05733.x PMC3245960

[jev212084-bib-0017] Furman, M. I. , Barnard, M. R. , Krueger, L. A. , Fox, M. L. , Shilale, E. A. , Lessard, D. M. , Marchese, P. , Frelinger, A. L. , Goldberg, R. J. , & Michelson, A. D. (2001). Circulating monocyte‐platelet aggregates are an early marker of acute myocardial infarction. Journal of the American College of Cardiology, 38(4), 1002–1006. 11583872 10.1016/s0735-1097(01)01485-1

[jev212084-bib-0018] Furman, M. I. , Benoit, S. E. , Barnard, M. R. , Valeri, C. R. , Borbone, M. L. , Becker, R. C. , Hechtman, H. B. , & Michelson, A. D. (1998). Increased platelet reactivity and circulating monocyte‐platelet aggregates in patients with stable coronary artery disease. Journal of the American College of Cardiology, 31(2), 352–358. 9462579 10.1016/s0735-1097(97)00510-x

[jev212084-bib-0019] Gkaliagkousi, E. , Corrigall, V. , Becker, S. , De Winter, P. , Shah, A. , Zamboulis, C. , Ritter, J. , & Ferro, A. (2009). Decreased platelet nitric oxide contributes to increased circulating monocyte‐platelet aggregates in hypertension. European Heart Journal, 30(24), 3048–3054. 19687162 10.1093/eurheartj/ehp330

[jev212084-bib-0020] Graff, J. , Harder, S. , Wahl, O. , Scheuermann, E. , & Gossmann, J. (2005). Anti‐inflammatory effects of clopidogrel intake in renal transplant patients: Effects on platelet‐leukocyte interactions, platelet CD40 ligand expression, and proinflammatory biomarkers. Clinical Pharmacology and Therapeutics, 78(5), 468–476. 16321613 10.1016/j.clpt.2005.08.002

[jev212084-bib-0021] Guduric‐Fuchs, J. , O'connor, A. , Camp, B. , O'neill, C. L. , Medina, R. J. , & Simpson, D. A. (2012). Selective extracellular vesicle‐mediated export of an overlapping set of microRNAs from multiple cell types. Bmc Genomics [Electronic Resource], 13(1), 357. 10.1186/1471-2164-13-357 PMC3532190 22849433

[jev212084-bib-0022] Hargett, L. A. , & Bauer, N. N. (2013). On the origin of microparticles: From “platelet dust” to mediators of intercellular communication. Pulmonary Circulation, 3, 329–340. 24015332 10.4103/2045-8932.114760 PMC3757826

[jev212084-bib-0023] Headland, S. E. , Jones, H. R. , D'sa, A. S. V. , Perretti, M. , & Norling, L. V. (2015). Analysis of Extracellular Microparticles using ImageStreamX Imaging Flow Cytometry. Scientific Reports, 4(1), 5237. 10.1038/srep05237 PMC4050385 24913598

[jev212084-bib-0024] Hoyer, F. F. , Giesen, M. K. , Nunes França, C. , Lütjohann, D. , Nickenig, G. , & Werner, N. (2012). Monocytic microparticles promote atherogenesis by modulating inflammatory cells in mice. Journal of Cellular and Molecular Medicine, 16(11), 2777–2788. 22697268 10.1111/j.1582-4934.2012.01595.x PMC4118246

[jev212084-bib-0025] Huber, S. A. , Sakkinen, P. , Conze, D. , Hardin, N. , & Tracy, R. (1999). Interleukin‐6 Exacerbates Early Atherosclerosis in Mice. Arteriosclerosis, Thrombosis, and Vascular Biology, 19(10), 2364–2367. 10.1161/01.atv.19.10.2364 10521365

[jev212084-bib-0026] Huo, Y. , Schober, A. , Forlow, S. B. , Smith, D. F. , Hyman, M. C. , Jung, S. , Littman, D. R. , Weber, C. , & Ley, K. (2003). Circulating activated platelets exacerbate atherosclerosis in mice deficient in apolipoprotein E. Nature Medicine, 9(1), 61–67. 10.1038/nm810 12483207

[jev212084-bib-0027] Jansen, F. , Li, Q. , Pfeifer, A. , & Werner, N. (2017). Endothelial‐ and immune cell‐derived extracellular vesicles in the regulation of cardiovascular health and disease. JACC. Basic to Translational Science, 2(6), 790–807. 30062186 10.1016/j.jacbts.2017.08.004 PMC6059011

[jev212084-bib-0028] Jansen, F. , Nickenig, G. , & Werner, N. (2017). Extracellular vesicles in cardiovascular disease: Potential applications in diagnosis, prognosis, and epidemiology. Circulation Research, 120(10), 1649–1657. 28495995 10.1161/CIRCRESAHA.117.310752

[jev212084-bib-0029] Jeppesen, D. K. , Fenix, A. M. , Franklin, J. L. , Higginbotham, J. N. , Zhang, Q. , Zimmerman, L. J. , Liebler, D. C. , Ping, J. , Liu, Qi , Evans, R. , Fissell, W. H. , Patton, J. G. , Rome, L. H. , Burnette, D. T. , & Coffey, R. J. (2019). Reassessment of exosome composition. Cell, 177(2), 428–445.e18.e18. 30951670 10.1016/j.cell.2019.02.029 PMC6664447

[jev212084-bib-0030] Kaneva, M K. , Greco, K V. , Headland, S E. , Montero‐Melendez, T. , Mori, P. , Greenslade, K. , Pitzalis, C. , Moore, A. , Perretti, M. (2017). Identification of novel chondroprotective mediators in resolving inflammatory exudates. Journal of Immunology, 198(7), 2876–2885. 10.4049/jimmunol.1601111 28242648

[jev212084-bib-0031] Kuravi, S. J. , Harrison, P. , Rainger, G. E.d , & Nash, G. B. (2019). Ability of platelet‐derived extracellular vesicles to promote neutrophil‐endothelial cell interactions. Inflammation., 42(1), 290–305. 30218321 10.1007/s10753-018-0893-5 PMC6394582

[jev212084-bib-0032] Leroyer, A. S. , Isobe, H. , Lesèche, G. , Castier, Y. , Wassef, M. , Mallat, Z. , Binder, B. R. , Tedgui, A. , & Boulanger, C. M. (2007). Cellular origins and thrombogenic activity of microparticles isolated from human atherosclerotic plaques. Journal of the American College of Cardiology, 49(7), 772–777. 17306706 10.1016/j.jacc.2006.10.053

[jev212084-bib-0033] Leroyer, A. S. , Rautou, P. ‐. E. , Silvestre, J. ‐. S. , Castier, Y. , Lesèche, G. , Devue, C. , Duriez, M. , Brandes, R. P. , Lutgens, E. , Tedgui, A. , & Boulanger, C. M. (2008). CD40 Ligand+ microparticles from human atherosclerotic plaques stimulate endothelial proliferation and angiogenesis. Journal of the American College of Cardiology, 52(16), 1302–1311. 18929241 10.1016/j.jacc.2008.07.032

[jev212084-bib-0034] Libby, P. , & Rocha, V. Z. (2018). All roads lead to IL‐6: A central hub of cardiometabolic signaling. International Journal of Cardiology, 259, 213–215. 29579604 10.1016/j.ijcard.2018.02.062

[jev212084-bib-0035] Libregts, S. F. W. M. , Arkesteijn, G. J. A. , Németh, A. , Nolte‐’T Hoen, E. N. M. , & Wauben, M. H. M. (2018). Flow cytometric analysis of extracellular vesicle subsets in plasma: Impact of swarm by particles of non‐interest. Journal of Thrombosis and Haemostasis, 16(7), 1423–1436. 29781099 10.1111/jth.14154

[jev212084-bib-0036] Mayr, M. , Grainger, D. , Mayr, U. , Leroyer, A. S. , Leseche, G. , Sidibe, A. , Herbin, O. , Yin, X. , Gomes, A. , Madhu, B. , Griffiths, J. R. , Xu, Q. , Tedgui, A. , & Boulanger, C. M. (2009). Proteomics, metabolomics, and immunomics on microparticles derived from human atherosclerotic plaques. Circulation. Cardiovascular Genetics, 2(4), 379–388. 20031610 10.1161/CIRCGENETICS.108.842849

[jev212084-bib-0037] Morel, O. , Toti, F. , Bakouboula, B. , Grunebaum, L. , & Freyssinet, J. ‐ M. (2006). Procoagulant microparticles: ‘Criminal partners’ in atherothrombosis and deleterious cellular exchanges. Pathophysiology of Haemostasis and Thrombosis, 35(1–2), 15–22. 16855341 10.1159/000093538

[jev212084-bib-0038] Neumann, F. J. , Zohlnhöfer, D. , Fakhoury, L. , Ott, I. , Gawaz, M. , & Schömig, A. (1999). Effect of glycoprotein IIb/IIIa receptor blockade on platelet‐leukocyte interaction and surface expression of the leukocyte integrin Mac‐1 in acute myocardial infarction. Journal of the American College of Cardiology, 34(5), 1420–1426. 10551687 10.1016/s0735-1097(99)00350-2

[jev212084-bib-0039] Nichols, M. , Townsend, N. , , Scarborough, P. , & Rayner, M. (2013). European cardiovascular disease statistics. European Heart Network and European Society of Cardiology, 34(39), 3028–3034. 10.1093/eurheartj/eht356 24014390

[jev212084-bib-0040] Norling, L. V. , Spite, M. , Yang, R. , Flower, R. J. , Perretti, M. , & Serhan, C. N. (2011). Cutting edge: Humanized nano‐proresolving medicines mimic inflammation‐resolution and enhance wound healing. Journal of Immunology, 186(10), 5543–5547. 10.4049/jimmunol.1003865 PMC3145138 21460209

[jev212084-bib-0041] Oggero, S. , Austin‐Williams, S. , & Norling, L. V. (2019). The contrasting role of extracellular vesicles in vascular inflammation and tissue repair. Frontiers in Pharmacology, 10. 10.3389/fphar.2019.01479 PMC6928593 31920664

[jev212084-bib-0042] Passacquale, G. , Vamadevan, P. , Pereira, L. , Hamid, C. , Corrigall, V. , & Ferro, A. (2011). Monocyte‐platelet interaction induces a pro‐inflammatory phenotype in circulating monocytes. In Q. Xu , (Ed.), Plos One, 6(10), e25595. 22022418 10.1371/journal.pone.0025595 PMC3192052

[jev212084-bib-0043] Patko, Z. , Csaszar, A. , Acsady, G. , Peter, K. , & Schwarz, M. (2012). Roles of Mac‐1 and glycoprotein IIb/IIIa integrins in leukocyte–platelet aggregate formation: Stabilization by Mac‐1 and inhibition by GpIIb/IIIa blockers. Platelets, 23(5), 368–375. 22671289 10.3109/09537104.2011.625098

[jev212084-bib-0044] Perez‐Riverol, Y. , Csordas, A. , Bai, J. , Bernal‐Llinares, M. , Hewapathirana, S. , Kundu, D. J. , Inuganti, A. , Griss, J. , Mayer, G. , Eisenacher, M. , Pérez, E. , Uszkoreit, J. , Pfeuffer, J. , Sachsenberg, T. , Yılmaz, Ş. , Tiwary, S. , Cox, J. , Audain, E. , Walzer, M. … Vizcaíno, J. A. (2019). The PRIDE database and related tools and resources in 2019: Improving support for quantification data. Nucleic Acids Research, 47(D1), D442–D450. 30395289 10.1093/nar/gky1106 PMC6323896

[jev212084-bib-0045] Pienimaeki‐Roemer, A. , Kuhlmann, K. , Böttcher, A. , Konovalova, T. , Black, A. , Orsó, E. , Liebisch, G. , Ahrens, M. , Eisenacher, M. , Meyer, H. E. , & Schmitz, G. (2015). Lipidomic and proteomic characterization of platelet extracellular vesicle subfractions from senescent platelets. Transfusion, 55(3), 507–521. 25332113 10.1111/trf.12874

[jev212084-bib-0046] Protogerou, A. D. , Zampeli, E. , Fragiadaki, K. , Stamatelopoulos, K. , Papamichael, C. , & Sfikakis, P. P. (2011). A pilot study of endothelial dysfunction and aortic stiffness after interleukin‐6 receptor inhibition in rheumatoid arthritis. Atherosclerosis, 219(2), 734–736. 21968316 10.1016/j.atherosclerosis.2011.09.015

[jev212084-bib-0047] Pucci, F. , Garris, C. , Lai, C. P. , Newton, A. , Pfirschke, C. , Engblom, C. , Alvarez, D. , Sprachman, M. , Evavold, C. , Magnuson, A. , Von Andrian, U. H. , Glatz, K. , Breakefield, X. O. , Mempel, T. R. , Weissleder, R. , & Pittet, M. J. (2016). SCS macrophages suppress melanoma by restricting tumor‐derived vesicle‐B cell interactions. Science (80‐), 352(6282), 242–246. 10.1126/science.aaf1328 PMC4960636 26989197

[jev212084-bib-0048] Raposo, G. , Nijman, H. W. , Stoorvogel, W. , Liejendekker, R. , Harding, C. V. , Melief, C. J. , & Geuze, H. J. (1996). B lymphocytes secrete antigen‐presenting vesicles. Journal of Experimental Medicine, 183(3), 1161–1172. 10.1084/jem.183.3.1161 PMC2192324 8642258

[jev212084-bib-0049] Sarma, J. , Laan, C. A. , Alam, S. , Jha, A. , Fox, K. A. A. , & Dransfield, I. (2002). Increased platelet binding to circulating monocytes in acute coronary syndromes. Circulation, 105(18), 2166–2171. 11994250 10.1161/01.cir.0000015700.27754.6f

[jev212084-bib-0050] Sun, H. Q. , Yamamoto, M. , Mejillano, M. , & Yin, H. L. (1999). Gelsolin, a Multifunctional Actin Regulatory Protein. The Journal of Biological Chemistry, 274(47), 33179–33182. 10559185 10.1074/jbc.274.47.33179

[jev212084-bib-0051] Swerdlow, D. I. , Holmes, M. V. , Kuchenbaecker, K. B. , Engmann, J. E. L. , Shah, T. , Sofat, R. , Guo, Y. , Chung, C. , Peasey, A. , Pfister, R. , Mooijaart, S. P. , Ireland, H. A. , Leusink, M. , Langenberg, C. , Li, K. W. , Palmen, J. , Howard, P. , Cooper, J. A. , Drenos, F. , … Casas, J. P. (2012). The interleukin‐6 receptor as a target for prevention of coronary heart disease: A Mendelian randomisation analysis. Lancet, 379(9822), 1214–1224. 22421340 10.1016/S0140-6736(12)60110-X PMC3316968

[jev212084-bib-0052] Tang, N. , Sun, B. , Gupta, A. , Rempel, H. , & Pulliam, L. (2016). Monocyte exosomes induce adhesion molecules and cytokines via activation of NF‐κB in endothelial cells. Faseb Journal, 30(9), 3097–3106. 27226520 10.1096/fj.201600368RR PMC5001509

[jev212084-bib-0053] Van Der Vorst, E. P. C. , De Jong, R. J. , & Donners, M. M. P. C. (2018). Message in a Microbottle: Modulation of Vascular Inflammation and Atherosclerosis by Extracellular Vesicles. Frontiers in Cardiovascular Medicine, 5. 10.3389/fcvm.2018.00002 PMC5786527 29404342

[jev212084-bib-0054] Wang, J. G. , Williams, J. C. , Davis, B. K. , Jacobson, K. , Doerschuk, C. M. , Ting, J. P.‐Y. , & Mackman, N. (2011). Monocytic microparticles activate endothelial cells in an IL‐1β‐dependent manner. Blood, 118(8), 2366–2374. 21700772 10.1182/blood-2011-01-330878 PMC3162361

[jev212084-bib-0055] Wei, H. , Malcor, J‐D. M. , & Harper, M. T. (2018). Lipid rafts are essential for release of phosphatidylserine‐exposing extracellular vesicles from platelets. Scientific Reports, 8(1), 9987. 29968812 10.1038/s41598-018-28363-4 PMC6030044

[jev212084-bib-0056] Wiśniewski, J. R. , Zougman, A. , Nagaraj, N. , & Mann, M. (2009). Universal sample preparation method for proteome analysis. Nature Methods, 6(5), 359–362. 19377485 10.1038/nmeth.1322

[jev212084-bib-0057] Wrigley, B. J. , Shantsila, E. , Tapp, L. D. , & Lip, G. Y. H. (2013). Increased formation of monocyte‐platelet aggregates in ischemic heart failure. Circulation Heart Failure. 6(1), 127–135. 23152489 10.1161/CIRCHEARTFAILURE.112.968073

[jev212084-bib-0058] Zaldivia, M. T. K. , Mcfadyen, J. D. , Lim, B. , Wang, X. , & Peter, K. (2017). Platelet‐Derived Microvesicles in Cardiovascular Diseases. Frontiers in Cardiovascular Medicine, 4, 74. 29209618 10.3389/fcvm.2017.00074 PMC5702324

[jev212084-bib-0059] Zaldivia, M. T. K. , Hering, D. , Marusic, P. , Sata, Y. , Lee, R. , Esler, M. D. , Htun, N. M. , Duval, J. , Hammond, L. , Flierl, U. , Wang, X. , Drummond, G. R. , Walton, A. , Gardiner, E. E. , Andrews, R. K. , Schlaich, M. P. , & Peter, K. (2020). Successful renal denervation decreases the platelet activation status in hypertensive patients. Cardiovascular Research, 116(1), 202–210. 30715163 10.1093/cvr/cvz033

