# Supplementary material for: Extracellular vesicles from monocyte/platelet aggregates modulate human atherosclerotic plaque reactivity
Source: J Extracell Vesicles. 2021 Apr 27;10(6):12084. doi: 10.1002/jev2.12084 (PMC8077084; doi:10.1002/jev2.12084)
Supplement: Supplementary file 1 — Supporting Information [file JEV2-10-12084-s001.docx]

**List of Supplementary Figures**

**Supplementary Figure S1: Monocyte and platelet aggregation does not occur spontaneously.** Monocytes (1x10^6^/ml) isolated using the density gradient purification protocol or whole blood were incubated with vehicle (V), TNF-α (50 ng/ml) or PAF (1 µM) for 60 min. Aggregate formation was monitored by Flow Cytometry or IS^x^ and non-treated samples were used as further control (CNTL). (a) Gating strategy to monitor CD14 and CD41 aggregation in PBMCs preparation in response to stimulation with TNF-α, PAF or PBS (V). (b) Frequency of monocytes (CD14^+^CD41^-^) in PBMC preparations unstimulated or stimulated for an hour with Vehicle, TNF-α or PAF. (c) Frequency of monocyte/platelet aggregates (CD14^+^CD41^+^) in PBMC preparations unstimulated or stimulated for an hour with Vehicle, TNF-α or PAF. (d) Frequency of platelets (CD14^-^CD41^+^) in PBMC preparations unstimulated or stimulated for an hour with Vehicle, TNF-α or PAF. (*p<0.05, **p<0.01, ***p< 0.001, one-way ANOVA post Bonferroni test, mean ± SEM of n=3 with different donor cells). (e) Figures from IS^x^ of monocyte and platelet aggregates formed upon stimulation of blood drawn from healthy volunteers with either 3.6 %w/v sodium citrate or 3.6%w/v sodium citrate added with 2μM Iloprost (PGI_2_). Ch01 represents brightfield, Ch03 is CD41 fluorescence, Ch11 shows CD14 fluorescence, Ch12 the SSC and Ch03/Ch11 is a mask where fluorescence of the two channels were merged. (f) Changes in percentages of aggregate development when blood is stimulated with veh (blue bars), TNF-α (red bars) or PAF (green bars). Data show a comparison among samples not treated with PGI_2_ or previously treated with it. A drop in the percentages was appreciated while PGI_2_ was added to PAF stimulated blood. (n=3 ± S.D.; Two-way Anova, *** p<0.0001). Veh: PBS stimulation, TNF-α: TNF-α sti*mulation, PAF: PAF stimulation.)*


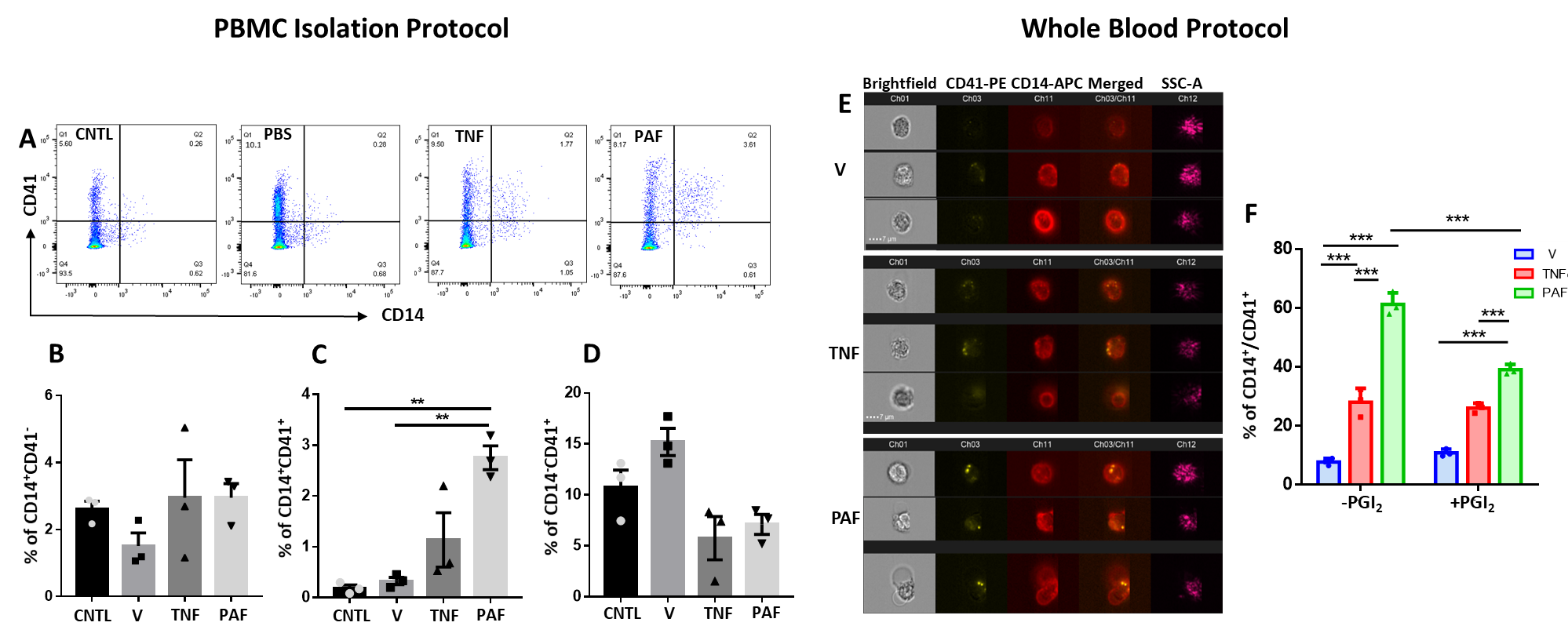


**Supplementary Figure S2. Lack of swarming effect in the analysis of double positive EVs.** Serial dilutions of EVs isolated from monocyte/platelet aggregates obtained by RosetteSep™ kit purification were run on ImageStream^x^ and frequency of double positive events was quantified. (a) Gatings showing frequency undiluted EV sample. (b) 1: 10 EV sample dilution; (c) 1:100 EV sample dilution; (d) 1:1000 EV sample dilution (e) Concentration of Bodipy^+^ EVs; (f) frequency of CD14^+^ EVs; (g) frequency of CD41^+^ EVs; (h) frequency of CD14^+^/CD41^+^ EVs. One-way ANOVA post Bonferroni test, * p<0.05, ** p<0.01, *** p< 0.001 n=4 individual experiments. Data expressed as mean ± SEM.


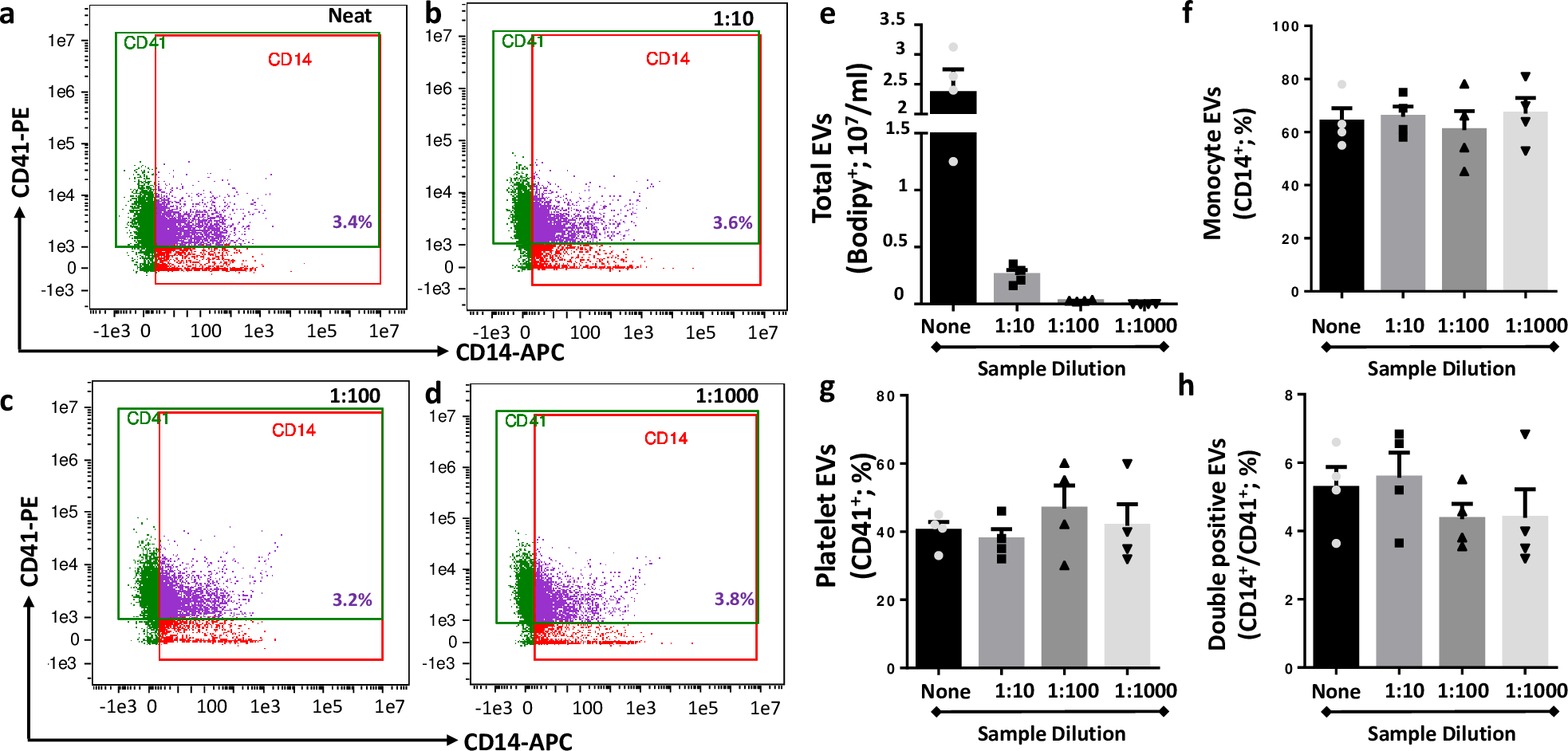


**Supplementary Figure S3. TEM images of EVs released by monocyte/platelet aggregates stimulated with TNF-α in presence and absence of TNF-α.** Monocytes were obtained and incubated with vehicle (V) or TNF-α (50 ng/ml), in presence or absence of Iloprost (2 µM; PGI_2_) for 60 min, as in Figure 2, to produce EVs. EVs from monocyte/platelet aggregates unstimulated in absence (a) or in presence of PGI_2_ (b) or stimulated with TNF-α (c) in absence (d) or in presence of PGI_2_ (e) were visualised by TEM.


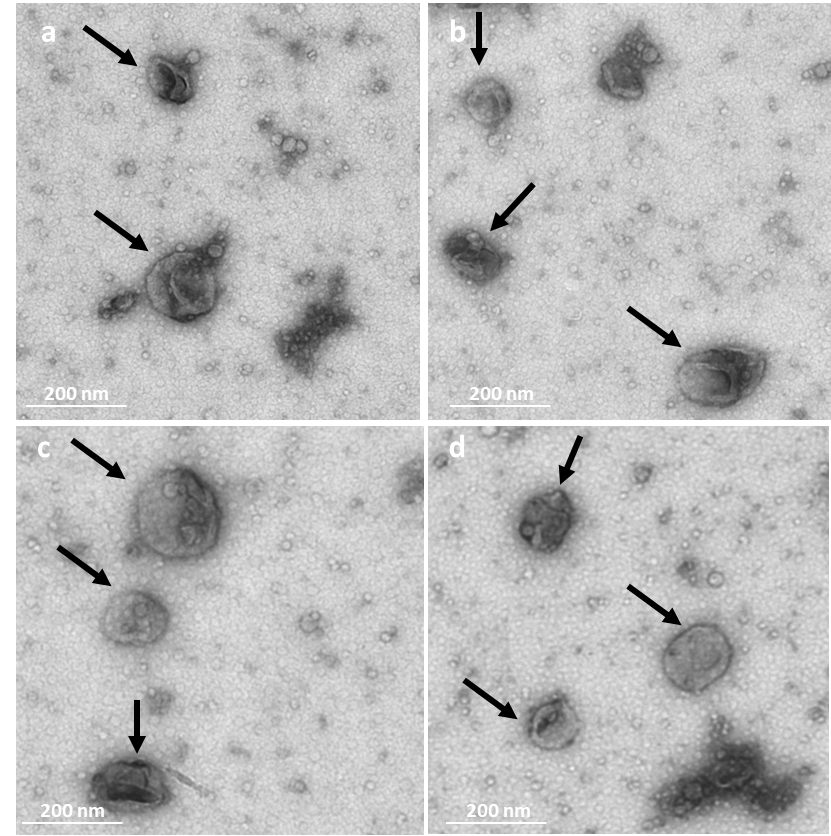


**Supplementary Figure S4. Monocyte/platelet EVs activate HAoEC in vitro.** EVs were collected from isolated monocyte/platelet aggregates, incubated with vehicle (V) or TNF-α (50 ng/ml) for 60 min, in presence or absence of Iloprost (2 µM; PGI_2_). HAoEC were incubated with the different EV sets (10x10^6^/ml) overnight. Cells were stained for flow cytomentry analysis and supernatants collected and analysed for cytokine release. (a) IL-6 levels by ELISA. (b-c) Quantification of ICAM-1 and VCAM-1 expression (MFI units) in HAoEC treated with different subsets of monocyte/platelet derived EVs. (*p<0.05, **p<0.01, ***p< 0.001; one-way ANOVA post Bonferroni test, mean ± SEM of n=3 cell preparations incubated with distinct EV preparations from different donor cells).


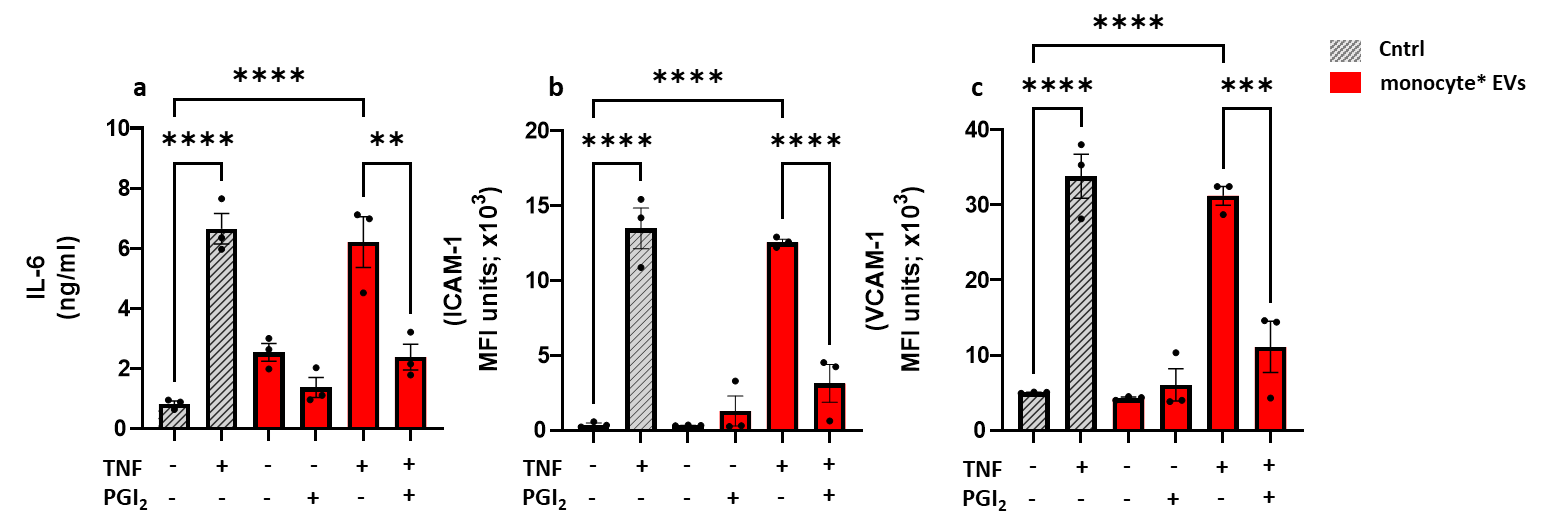


**List of Supplementary Tables**

**Supplementary Table S1. Demographic characteristics of patients undergoing endarterectomy.**

| Age (years ± SEM) | 71.8 ± 1.86 |
| --- | --- |
| Total Number | 5 |
| Male (%) | 83.3 |
| CEA frequency relative to gender, N (%) | 80% |
| FEA frequency relative to gender, N (%) | 10% |
| Length of plaque (cm ± SEM) | 3.6 ± 0.7 |

CEA: carotid endarterectomy

FEA: femoral endarterectomy

**Supplementary Table S2. Quantification of cytokines released from plaque supernatants after treatment with different EV subsets.** Monocyte were obtained as in Figure 2 and incubated with vehicle (V) or TNF-α (50 ng/ml), in presence or absence of Iloprost (2 µM; PGI_2_) for 60 min. Human atherosclerotic plaque fragments were incubated with the reported EVs (10x10^6^/ml) overnight. Supernatants were collected and used for ELISA analysis. Data are pg/ml, mean ± SD of n=5 plaques incubated with distinct EV preparations with different donor cells.

|  | Media alone | Without EVs | Veh EVs | Veh+ PGI_2_ EVs | TNF-α EVs | TNF-α+ PGI_2_ Evs |
| --- | --- | --- | --- | --- | --- | --- |
| TNF-α | 1.14 ± 0.66 | 3.788 ± 3.43 | 6.783 ± 1.60 | 6.9582 ± 3.07 | 609.4662 ± 213.2 (**) | 251.976 ± 27.46 |
| MIP-1α | 22.26 ± 23.99 | 229.29 ± 278.3 | 300.05 ± 304.8 | 381.86 ± 673.1 | 1908.74 ± 2206 | 289.25 ± 128.4 |
| IL-4 | 0.14 ± 0.06 | 0.39 ± 0.52 | 0.28 ± 0.18 | 0.33 ± 0.44 | 0.71 ± 0.47 | 0.22 ± 0.06 |
| IL-1β | 0.22 ± 0.40 | 0.15 ± 0.23 | 0.24 ± 0.27 | 0.41 ± 0.55 | 1.84 ± 2.19 | 1.28 ± 2.23 |
| IL-6 | 0.37 ± 2.00 | 50.53 ± 31.06 | 132.48 ± 198.7 | 600.99 ± 493.3 | 4762.74 ± 2128 (***) | 833.15 ± 589.5 |
| IL-13 | 0.85 ± 0.37 | 12.28 ± 8.31 | 77.62 ± 1.35 | 8.86 ± 4.80 | 773.02 ± 6.17 (***) | 232.31 ± 1.74 |
| IL-10 | 0.13 ± 0.22 | 0.2789086 ± 0.33 | 0.42 ± 0.63 | 0.44 ± 0.37 | 2.10 ± 1.90 | 0.83 ± 0.87 |
| INF-ϒ | 3.12 ± 5.45 | 22.54 ± 46.95 | 14.45 ±23.62 | 20.03 ± 29.72 | 92.52 ± 83.22 (*) | 11.80 ± 11.46 |
| GMCSF | 0.13 ± 0.18 | 1.08 ± 1.79 | 0.99 ± 1.05 | 0.72 ± 0.88 | 14.23 ± 14.29 | 1.19 ± 1.66 |

**Supplementary Table S3. Mass spectrometry details of the 681 proteins identified in monocyte EVs.**

| **Gene names** | **Protein names** | **Protein IDs** | **Sequence coverage [%]** | **Unique sequence coverage [%]** | **Peptides** | **Unique peptides** | **Mol. weight [kDa]** | **Intensity** | **MS/MS Count** | **Coefficient of variation** |
| --- | --- | --- | --- | --- | --- | --- | --- | --- | --- | --- |
| A1BG | Alpha-1B-glycoprotein | P04217 | 19.8 | 19.8 | 8 | 8 | 54.253 | 1494200000 | 56 | 0.0547 |
| A2M | Alpha-2-macroglobulin | P01023 | 46.9 | 38.4 | 54 | 42 | 163.29 | 29330000000 | 615 | 0.0176 |
| AARS | Alanine--tRNA ligase, cytoplasmic | P49588 | 11.8 | 11.8 | 10 | 10 | 106.81 | 202460000 | 19 | 0.0476 |
| ABI1 | Abl interactor 1 | B6VEX4 | 17.6 | 17.6 | 6 | 6 | 43.294 | 161980000 | 14 | 0.0357 |
| ACAA2 | 3-ketoacyl-CoA thiolase, mitochondrial | A0A0B4J2A4 | 6.9 | 6.9 | 3 | 3 | 41.601 | 500210000 | 25 | 0.0580 |
| ACLY | ATP-citrate synthase | P53396 | 10.8 | 10.8 | 10 | 10 | 120.84 | 13204000000 | 54 | 0.0939 |
| ACO2 | Aconitate hydratase, mitochondrial | Q99798 | 10.1 | 10.1 | 8 | 8 | 85.424 | 1108700000 | 66 | 0.0496 |
| ACTA1 | Actin, alpha skeletal muscle | P68133 | 40.1 | 11.7 | 17 | 3 | 42.051 | 54936000000 | 130 | 0.0216 |
| ACTB | Actin, cytoplasmic 1 | P60709 | 71.5 | 4.5 | 23 | 1 | 41.736 | 9.7215E+11 | 1217 | 0.0072 |
| ACTN1 | Alpha-actinin-1 | P12814 | 60.8 | 43.5 | 52 | 37 | 103.06 | 48481000000 | 923 | 0.0118 |
| ACTN4 | Alpha-actinin-4 | O43707 | 55.5 | 38.6 | 44 | 29 | 104.85 | 1992900000 | 159 | 0.0375 |
| ACTR2 | Actin-related protein 2 | P61160 | 35.8 | 35.8 | 11 | 11 | 44.76 | 3311500000 | 120 | 0.0399 |
| ACTR3 | Actin-related protein 3 | P61158 | 44.7 | 44.7 | 14 | 14 | 47.371 | 4139000000 | 141 | 0.0089 |
| ADH5 | Alcohol dehydrogenase class-3 | P11766 | 31 | 31 | 10 | 10 | 39.724 | 323560000 | 35 | 0.0431 |
| AFM | Afamin | P43652 | 20.4 | 20.4 | 13 | 13 | 69.068 | 590250000 | 42 | 0.0447 |
| AGT | Angiotensinogen | P01019 | 11.5 | 11.5 | 5 | 5 | 53.154 | 724060000 | 38 | 0.0609 |
| AHCY | Adenosylhomocysteinase | P23526 | 30.6 | 30.6 | 14 | 14 | 47.716 | 1152000000 | 67 | 0.0314 |
| AHNAK | Neuroblast differentiation-associated protein AHNAK | Q09666 | 12.5 | 12.5 | 17 | 17 | 629.09 | 891290000 | 51 | 0.0536 |
| AHSG | Alpha-2-HS-glycoprotein | P02765 | 31.6 | 26.2 | 9 | 6 | 39.324 | 4982600000 | 76 | 0.0387 |
| AK1 | Adenylate kinase isoenzyme 1 | P00568 | 18 | 18 | 3 | 3 | 21.635 | 682510000 | 23 | 0.0497 |
| AKR1D1 | 3-oxo-5-beta-steroid 4-dehydrogenase | P51857 | 3.1 | 3.1 | 2 | 2 | 37.376 | 87609000 | 5 | 0.0288 |
| AKR7A2 | Aflatoxin B1 aldehyde reductase member 2 | H3BLU7 | 24.2 | 24.2 | 6 | 6 | 34.684 | 473350000 | 36 | 0.0424 |
| ALDH2 | Aldehyde dehydrogenase, mitochondrial | P05091 | 15.3 | 15.3 | 8 | 8 | 56.381 | 367670000 | 30 | 0.0706 |
| ALDOA | Fructose-bisphosphate aldolase A | P04075 | 76.6 | 63.2 | 26 | 20 | 39.42 | 27716000000 | 377 | 0.0098 |
| ALDOB | Fructose-bisphosphate aldolase B | P05062 | 32.1 | 28.3 | 10 | 8 | 39.473 | 1276200000 | 61 | 0.0635 |
| ALDOC | Fructose-bisphosphate aldolase C | P09972 | 40.9 | 25.5 | 15 | 8 | 39.455 | 735850000 | 44 | 0.0576 |
| ALOX12 | Arachidonate 12-lipoxygenase, 12S-type | P18054 | 21.9 | 21.9 | 12 | 12 | 75.693 | 702180000 | 47 | 0.0372 |
| AMBP | Protein AMBP | P02760 | 17.6 | 15.6 | 6 | 5 | 38.999 | 810510000 | 40 | 0.0520 |
| AMPD2 | AMP deaminase 2 | H0Y360 | 8.1 | 8.1 | 7 | 7 | 98.401 | 193660000 | 19 | 0.0364 |
| ANXA1 | Annexin A1 | P04083 | 50.9 | 50.9 | 16 | 16 | 38.714 | 3606800000 | 134 | 0.0250 |
| ANXA11 | Annexin A11 | P50995 | 27.1 | 25.3 | 13 | 12 | 54.389 | 976360000 | 43 | 0.0632 |
| ANXA2 | Annexin A2 | P07355 | 60.8 | 60.8 | 20 | 20 | 38.604 | 6752700000 | 201 | 0.0217 |
| ANXA4 | Annexin | Q6P452 | 24.4 | 24.4 | 7 | 7 | 33.552 | 523280000 | 19 | 0.0724 |
| ANXA5 | Annexin A5 | P08758 | 49.1 | 49.1 | 15 | 15 | 35.936 | 3446600000 | 110 | 0.0455 |
| ANXA6 | Annexin A6 | P08133 | 42.8 | 42.8 | 29 | 29 | 75.872 | 5613000000 | 176 | 0.0977 |
| ANXA7 | Annexin A7 | P20073 | 27 | 27 | 12 | 12 | 52.739 | 720560000 | 51 | 0.0419 |
| AP1B1 | AP-1 complex subunit beta-1 | Q10567 | 20 | 8.6 | 17 | 7 | 104.64 | 810430000 | 60 | 0.0440 |
| AP1G1 | AP-1 complex subunit gamma-1 | O43747 | 5.6 | 5.6 | 5 | 5 | 91.35 | 288680000 | 31 | 0.0388 |
| AP2M1 | AP-2 complex subunit mu | A0A087WY71 | 10.1 | 10.1 | 5 | 5 | 49.526 | 121900000 | 10 | 0.0402 |
| APCS | Serum amyloid P-component | P02743 | 33.6 | 33.6 | 7 | 7 | 25.387 | 1669000000 | 67 | 0.0349 |
| APOA1 | Apolipoprotein A-I | P02647 | 75.7 | 69.7 | 25 | 24 | 30.777 | 1.4573E+11 | 501 | 0.0127 |
| APOA2 | Apolipoprotein A-II | V9GYE3 | 84.6 | 84.6 | 7 | 7 | 5.8767 | 17249000000 | 114 | 0.0147 |
| APOA4 | Apolipoprotein A-IV | P06727 | 46.5 | 46.5 | 19 | 19 | 45.398 | 2879500000 | 126 | 0.0453 |
| APOB | Apolipoprotein B-100 | P04114 | 29.2 | 29.2 | 125 | 125 | 515.6 | 10646000000 | 655 | 0.0663 |
| APOC1 | Apolipoprotein C-I | K7ERI9 | 26 | 26 | 3 | 3 | 8.647 | 3403900000 | 39 | 0.0863 |
| APOC2 | Apolipoprotein C-II | Q6P163 | 40.3 | 40.3 | 3 | 3 | 8.1463 | 894870000 | 35 | 0.0773 |
| APOC3 | Apolipoprotein C-III | P02656 | 34.3 | 34.3 | 3 | 3 | 10.852 | 5969200000 | 72 | 0.0167 |
| APOD | Apolipoprotein D | P05090 | 34.4 | 34.4 | 6 | 6 | 21.275 | 2258000000 | 66 | 0.0156 |
| APOE | Apolipoprotein E | P02649 | 44.5 | 44.5 | 13 | 13 | 36.154 | 1534000000 | 94 | 0.0490 |
| APOH | Beta-2-glycoprotein 1 | P02749 | 20.3 | 20.3 | 5 | 5 | 38.298 | 785950000 | 42 | 0.0429 |
| APP | Amyloid beta A4 protein | P05067 | 29.5 | 28.4 | 20 | 19 | 86.942 | 3496000000 | 137 | 0.0416 |
| ARCN1 | Coatomer subunit delta | P48444 | 10.4 | 10.4 | 5 | 5 | 57.21 | 127220000 | 12 | 0.0440 |
| ARF1 | ADP-ribosylation factor 1 | P84077 | 45.9 | 24.3 | 7 | 4 | 20.697 | 5323800000 | 102 | 0.0149 |
| ARF4 | ADP-ribosylation factor 4 | P18085 | 35.6 | 19.4 | 6 | 4 | 20.511 | 423460000 | 32 | 0.0449 |
| ARG1 | Arginase-1 | P05089 | 18 | 18 | 6 | 6 | 34.735 | 368370000 | 25 | 0.0625 |
| ARHGAP1 | Rho GTPase-activating protein 1 | Q07960 | 27.8 | 27.8 | 10 | 10 | 50.435 | 748830000 | 49 | 0.0706 |
| ARHGAP18 | Rho GTPase-activating protein 18 | Q8N392 | 14.8 | 14.8 | 10 | 10 | 74.976 | 358860000 | 35 | 0.0437 |
| ARHGAP6 | Rho GTPase-activating protein 6 | O43182 | 3.1 | 3.1 | 3 | 3 | 105.95 | 145930000 | 12 | 0.0480 |
| ARHGDIA | Rho GDP-dissociation inhibitor 1 | J3KTF8 | 25.9 | 25.9 | 5 | 5 | 21.517 | 1222600000 | 71 | 0.0475 |
| ARHGDIB | Rho GDP-dissociation inhibitor 2 | F5H3P3 | 58.9 | 58.9 | 7 | 7 | 17.837 | 1662500000 | 57 | 0.0476 |
| ARPC1B | Actin-related protein 2/3 complex subunit 1B | O15143 | 37.1 | 37.1 | 12 | 12 | 40.949 | 2114100000 | 93 | 0.0112 |
| ARPC2 | Actin-related protein 2/3 complex subunit 2 | O15144 | 46.3 | 46.3 | 15 | 15 | 34.333 | 3096200000 | 120 | 0.0094 |
| ARPC3 | Actin-related protein 2/3 complex subunit 3 | O15145 | 17.4 | 17.4 | 3 | 3 | 20.546 | 785010000 | 32 | 0.0565 |
| ARPC4 | Actin-related protein 2/3 complex subunit 4 | P59998 | 41.1 | 41.1 | 6 | 6 | 19.667 | 2899700000 | 80 | 0.0095 |
| ARPC5 | Actin-related protein 2/3 complex subunit 5 | O15511 | 53.6 | 53.6 | 8 | 8 | 16.32 | 772370000 | 34 | 0.0527 |
| ASS1 | Argininosuccinate synthase | P00966 | 17.5 | 17.5 | 10 | 10 | 46.53 | 1309100000 | 64 | 0.0667 |
| ATP1A1 | Sodium/potassium-transporting ATPase subunit alpha-1 | P05023 | 26 | 8.2 | 25 | 8 | 112.89 | 618970000 | 41 | 0.0562 |
| ATP1A2 | Sodium/potassium-transporting ATPase subunit alpha-2 | B1AKY9 | 26.9 | 10.5 | 25 | 10 | 110.86 | 1065700000 | 61 | 0.0625 |
| ATP1A3 | Sodium/potassium-transporting ATPase subunit alpha-3 | P13637 | 30.5 | 13.1 | 28 | 12 | 111.75 | 9946800000 | 296 | 0.0755 |
| ATP1B1 | Sodium/potassium-transporting ATPase subunit beta-1 | P05026 | 13.2 | 13.2 | 4 | 4 | 35.061 | 2005000000 | 63 | 0.0507 |
| ATP2A2 | Sarcoplasmic/endoplasmic reticulum calcium ATPase 2 | P16615 | 9.5 | 5.3 | 9 | 5 | 114.76 | 388460000 | 25 | 0.0492 |
| ATP5A1 | ATP synthase subunit alpha, mitochondrial | P25705 | 46.1 | 46.1 | 26 | 26 | 59.75 | 8080400000 | 218 | 0.0651 |
| ATP5B | ATP synthase subunit beta, mitochondrial | P06576 | 47.1 | 47.1 | 19 | 19 | 56.559 | 7996100000 | 224 | 0.0631 |
| ATP5C1 | ATP synthase subunit gamma, mitochondrial | P36542 | 15.4 | 15.4 | 5 | 5 | 32.996 | 139580000 | 15 | 0.0366 |
| ATP6V1A | V-type proton ATPase catalytic subunit A | P38606 | 32.1 | 32.1 | 17 | 17 | 68.303 | 1205000000 | 96 | 0.0328 |
| ATP6V1B2 | V-type proton ATPase subunit B, brain isoform | P21281 | 25.4 | 25.4 | 10 | 10 | 56.5 | 920180000 | 57 | 0.0335 |
| AZGP1 | Zinc-alpha-2-glycoprotein | P25311 | 34.2 | 34.2 | 11 | 11 | 34.258 | 1246600000 | 68 | 0.0380 |
| B2M | Beta-2-microglobulin | H0YLF3 | 28.2 | 28.2 | 2 | 2 | 8.4984 | 3881200000 | 43 | 0.0862 |
| BASP1 | Brain acid soluble protein 1 | P80723 | 31.3 | 31.3 | 7 | 7 | 22.693 | 295640000 | 42 | 0.0425 |
| BHMT | Betaine--homocysteine S-methyltransferase 1 | E5RJH0 | 20.2 | 20.2 | 5 | 5 | 28.206 | 550020000 | 27 | 0.0763 |
| BIN2 | Bridging integrator 2 | Q9UBW5 | 31.5 | 31.5 | 15 | 15 | 61.874 | 2946000000 | 121 | 0.0362 |
| C1QB | Complement C1q subcomponent subunit B | A0A0A0MSV6 | 15.8 | 15.8 | 3 | 3 | 24.03 | 325510000 | 23 | 0.0582 |
| C3 | Complement C3 | P01024 | 55.6 | 50.3 | 87 | 76 | 187.15 | 52757000000 | 1071 | 0.0150 |
| C4B | Complement C4-B | P0C0L5 | 31.2 | 27.5 | 49 | 41 | 192.75 | 9988900000 | 398 | 0.0251 |
| C4BPA | C4b-binding protein alpha chain | P04003 | 30.3 | 30.3 | 15 | 15 | 67.033 | 2562800000 | 133 | 0.0445 |
| C5 | Complement C5 | P01031 | 7.5 | 7.5 | 12 | 12 | 188.3 | 265600000 | 27 | 0.0391 |
| C9 | Complement component C9 | P02748 | 16.3 | 12.5 | 8 | 6 | 63.173 | 573690000 | 34 | 0.0501 |
| CA2 | Carbonic anhydrase 2 | P00918 | 37.7 | 37.7 | 8 | 8 | 29.246 | 1160600000 | 48 | 0.0875 |
| CALD1 | Caldesmon | E9PGZ1 | 38.1 | 2.4 | 24 | 3 | 61.705 | 4729000000 | 231 | 0.0365 |
| CALM2 | Calmodulin-2 | P0DP25 | 60.4 | 55.7 | 12 | 10 | 16.837 | 10567000000 | 186 | 0.0595 |
| CALML5 | Calmodulin-like protein 5 | Q9NZT1 | 74 | 74 | 9 | 9 | 15.892 | 1323100000 | 49 | 0.0805 |
| CALR | Calreticulin | P27797 | 27.3 | 27.3 | 11 | 11 | 48.141 | 3003500000 | 120 | 0.0167 |
| CALU | Calumenin | O43852 | 38.4 | 38.4 | 12 | 12 | 37.106 | 1576800000 | 65 | 0.0743 |
| CAMK2A | Calcium/calmodulin-dependent protein kinase type II subunit alpha | Q9UQM7 | 23.8 | 17.6 | 10 | 7 | 54.087 | 1372300000 | 61 | 0.0528 |
| CANX | Calnexin | P27824 | 12.2 | 12.2 | 7 | 7 | 67.567 | 382400000 | 29 | 0.0528 |
| CAP1 | Adenylyl cyclase-associated protein 1 | Q01518 | 47.4 | 47.4 | 21 | 21 | 51.901 | 22046000000 | 367 | 0.0088 |
| CAPG | Macrophage-capping protein | P40121 | 15.2 | 15.2 | 5 | 5 | 38.498 | 463900000 | 44 | 0.0597 |
| CAPN1 | Calpain-1 catalytic subunit | P07384 | 31 | 31 | 21 | 21 | 81.889 | 3680700000 | 143 | 0.0392 |
| CAPNS1 | Calpain small subunit 1 | P04632 | 22.8 | 22.8 | 6 | 6 | 28.315 | 1173900000 | 35 | 0.0685 |
| CAPZA1 | F-actin-capping protein subunit alpha-1 | P52907 | 55.6 | 46.2 | 11 | 9 | 32.922 | 3719700000 | 99 | 0.0411 |
| CAPZA2 | F-actin-capping protein subunit alpha-2 | P47755 | 37.4 | 28 | 7 | 5 | 32.949 | 382910000 | 24 | 0.0686 |
| CAPZB | F-actin-capping protein subunit beta | B1AK87 | 44.2 | 6.2 | 12 | 2 | 29.295 | 5063500000 | 156 | 0.0089 |
| CASP14 | Caspase-14 | P31944 | 42.1 | 42.1 | 12 | 12 | 27.679 | 2041900000 | 77 | 0.0921 |
| CAT | Catalase | P04040 | 31.9 | 31.9 | 15 | 15 | 59.755 | 1499400000 | 89 | 0.0472 |
| CCDC6 | Coiled-coil domain-containing protein 6 | Q16204 | 3.4 | 3.4 | 2 | 2 | 53.29 | 503650000 | 4 | 0.0553 |
| CCL5 | C-C motif chemokine 5 | P13501 | 39.6 | 39.6 | 4 | 4 | 9.9896 | 3750000000 | 47 | 0.0491 |
| CCT2 | T-complex protein 1 subunit beta | P78371 | 34.4 | 34.4 | 15 | 15 | 57.488 | 1292700000 | 89 | 0.0382 |
| CCT3 | T-complex protein 1 subunit gamma | P49368 | 32.5 | 32.5 | 18 | 18 | 60.533 | 1534800000 | 104 | 0.0351 |
| CCT4 | T-complex protein 1 subunit delta | P50991 | 28.9 | 28.9 | 15 | 15 | 57.924 | 1122500000 | 83 | 0.0295 |
| CCT5 | T-complex protein 1 subunit epsilon | P48643 | 34.8 | 34.8 | 18 | 18 | 59.67 | 1077700000 | 76 | 0.0466 |
| CCT6A | T-complex protein 1 subunit zeta | P40227 | 25.4 | 25.4 | 14 | 14 | 58.024 | 1355200000 | 87 | 0.0131 |
| CCT7 | T-complex protein 1 subunit eta | Q99832 | 29.1 | 29.1 | 14 | 14 | 59.366 | 808450000 | 52 | 0.0442 |
| CCT8 | T-complex protein 1 subunit theta | P50990 | 37.8 | 37.8 | 20 | 20 | 59.62 | 1580200000 | 103 | 0.0172 |
| CD36 | Platelet glycoprotein 4 | E7EU05 | 21 | 21 | 10 | 10 | 52.062 | 1649000000 | 54 | 0.0720 |
| CD47 | Leukocyte surface antigen CD47 | Q08722 | 8.7 | 8.7 | 3 | 3 | 35.213 | 163220000 | 9 | 0.0365 |
| CD5L | CD5 antigen-like | O43866 | 18.2 | 18.2 | 6 | 6 | 38.087 | 189400000 | 18 | 0.0479 |
| CD63 | CD63 antigen | F8VNT9 | 13.5 | 13.5 | 2 | 2 | 14.265 | 238650000 | 23 | 0.0326 |
| CD84 | SLAM family member 5 | Q9UIB8 | 5.8 | 5.8 | 2 | 2 | 38.782 | 613890000 | 3 | 0.0309 |
| CD9 | CD9 antigen | A6NNI4 | 10.7 | 10.7 | 2 | 2 | 17.764 | 3370100000 | 32 | 0.0907 |
| CDC37 | Hsp90 co-chaperone Cdc37 | K7EKQ2 | 9.6 | 9.6 | 4 | 4 | 41.659 | 233020000 | 19 | 0.0467 |
| CDC42 | Cell division control protein 42 homolog | P60953 | 39.3 | 33.5 | 7 | 6 | 21.258 | 1554800000 | 54 | 0.0548 |
| CDSN | Corneodesmosin | Q2L6G8 | 11.3 | 11.3 | 4 | 4 | 51.539 | 235140000 | 14 | 0.0491 |
| CDV3 | Protein CDV3 homolog | Q9UKY7 | 6.2 | 6.2 | 2 | 2 | 27.335 | 113540000 | 7 | 0.0429 |
| CFB | Complement factor B | B4E1Z4 | 22.4 | 22.4 | 25 | 25 | 140.94 | 3194400000 | 159 | 0.0228 |
| CFH | Complement factor H | P08603 | 34.1 | 32.5 | 33 | 31 | 139.09 | 2988000000 | 164 | 0.0613 |
| CFHR1 | Complement factor H-related protein 1 | B1AKG0 | 15.1 | 7.7 | 4 | 2 | 30.858 | 92392000 | 7 | 0.0443 |
| CFL1 | Cofilin-1 | P23528 | 69.3 | 65.1 | 15 | 14 | 18.502 | 37330000000 | 356 | 0.0082 |
| CKB | Creatine kinase B-type | P12277 | 20.7 | 20.7 | 6 | 6 | 42.644 | 2351700000 | 65 | 0.0503 |
| CKMT1A | Creatine kinase U-type, mitochondrial | P12532 | 11.3 | 11.3 | 4 | 4 | 47.036 | 545820000 | 30 | 0.0712 |
| CLC | Eosinophil lysophospholipase | Q05315 | 16.2 | 16.2 | 2 | 2 | 16.453 | 687540000 | 16 | 0.0879 |
| CLIC1 | Chloride intracellular channel protein 1 | O00299 | 69.7 | 69.7 | 12 | 12 | 26.922 | 10879000000 | 174 | 0.0080 |
| CLIC4 | Chloride intracellular channel protein 4 | Q9Y696 | 23.3 | 23.3 | 5 | 5 | 28.772 | 264260000 | 25 | 0.0416 |
| CLINT1 | Clathrin interactor 1 | Q14677 | 10.4 | 10.4 | 6 | 6 | 68.259 | 247160000 | 15 | 0.0439 |
| CLIP1 | CAP-Gly domain-containing linker protein 1 | P30622 | 1.1 | 1.1 | 2 | 2 | 162.24 | 26979000 | 2 | 0.0356 |
| CLTA | Clathrin light chain A | P09496 | 12.9 | 12.9 | 4 | 4 | 27.076 | 801640000 | 35 | 0.0522 |
| CLTC | Clathrin heavy chain 1 | Q00610 | 34.5 | 34.5 | 51 | 51 | 191.61 | 11659000000 | 458 | 0.0334 |
| CLU | Clusterin | P10909 | 29.8 | 29.8 | 14 | 14 | 52.494 | 13027000000 | 230 | 0.0106 |
| CNDP2 | Cytosolic non-specific dipeptidase | Q96KP4 | 23.2 | 23.2 | 10 | 10 | 52.878 | 722270000 | 46 | 0.0528 |
| CNN2 | Calponin-2 | B4DDF4 | 37.9 | 37.9 | 10 | 10 | 32.616 | 3457200000 | 99 | 0.0186 |
| CNP | 2,3-cyclic-nucleotide 3-phosphodiesterase | P09543 | 8.8 | 8.8 | 4 | 4 | 47.578 | 630510000 | 23 | 0.0793 |
| COL14A1 | Collagen alpha-1(XIV) chain | Q4G0W3 | 6.7 | 6.7 | 4 | 4 | 59.163 | 2012000000 | 43 | 0.0820 |
| COPA | Coatomer subunit alpha | P53621 | 15.4 | 15.4 | 18 | 18 | 138.34 | 399640000 | 39 | 0.0455 |
| COPB2 | Coatomer subunit beta | P35606 | 5.5 | 5.5 | 5 | 5 | 102.49 | 209240000 | 22 | 0.0360 |
| CORO1A | Coronin-1A | P31146 | 31.5 | 31.5 | 16 | 16 | 51.026 | 6812500000 | 182 | 0.0098 |
| CORO1B | Coronin-1B | Q9BR76 | 15.1 | 15.1 | 8 | 8 | 54.234 | 487650000 | 35 | 0.0485 |
| CORO1C | Coronin-1C | Q9ULV4 | 32.3 | 32.3 | 17 | 17 | 53.248 | 6660800000 | 207 | 0.0162 |
| COTL1 | Coactosin-like protein | Q14019 | 63.4 | 63.4 | 9 | 9 | 15.945 | 3514700000 | 103 | 0.0277 |
| COX5A | Cytochrome c oxidase subunit 5A, mitochondrial | H3BRM5 | 21.7 | 21.7 | 2 | 2 | 7.771 | 327390000 | 10 | 0.0489 |
| CP | Ceruloplasmin | P00450 | 25.6 | 25.6 | 23 | 23 | 122.2 | 5638900000 | 215 | 0.0365 |
| CPS1 | Carbamoyl-phosphate synthase [ammonia], mitochondrial | P31327 | 22.9 | 22.9 | 33 | 33 | 164.94 | 8981700000 | 277 | 0.0825 |
| CRIP1 | Cysteine-rich protein 1 | K4DIB9 | 60.9 | 60.9 | 2 | 2 | 4.7533 | 95214000 | 14 | 0.0521 |
| CS | Citrate synthase | B4DJV2 | 10.2 | 8.2 | 5 | 4 | 50.431 | 492840000 | 30 | 0.0665 |
| CSK | Tyrosine-protein kinase CSK | P41240 | 17.3 | 17.3 | 7 | 7 | 50.704 | 405480000 | 21 | 0.0527 |
| CSRP1 | Cysteine and glycine-rich protein 1 | P21291 | 64.2 | 64.2 | 11 | 11 | 20.567 | 4835600000 | 136 | 0.0131 |
| CST4 | Cystatin-S | P01036 | 66.7 | 35.5 | 7 | 3 | 16.214 | 637150000 | 29 | 0.1072 |
| CSTA | Cystatin-A | P01040 | 76.5 | 76.5 | 7 | 7 | 11.006 | 1104600000 | 40 | 0.0923 |
| CSTB | Cystatin-B | P04080 | 45.9 | 45.9 | 3 | 3 | 11.139 | 329230000 | 16 | 0.0565 |
| CTSA | Lysosomal protective protein | P10619 | 11.5 | 11.5 | 5 | 5 | 54.465 | 578630000 | 28 | 0.0565 |
| CTSD | Cathepsin D | A0A1B0GW44 | 29.9 | 29.9 | 10 | 10 | 43.688 | 1143400000 | 54 | 0.0762 |
| CTTN | Src substrate cortactin | Q14247 | 32.2 | 32.2 | 17 | 17 | 61.585 | 2526100000 | 97 | 0.0657 |
| CYC1 | Cytochrome c1, heme protein, mitochondrial | P08574 | 3.7 | 3.7 | 1 | 1 | 35.422 | 61658000 | 6 | 0.0442 |
| CYFIP1 | Cytoplasmic FMR1-interacting protein 1 | Q7L576 | 11.4 | 11.4 | 14 | 14 | 145.18 | 598400000 | 39 | 0.0516 |
| DAB2 | Disabled homolog 2 | P98082 | 1.6 | 1.6 | 1 | 1 | 82.447 | 5187600 | 1 | 0.0326 |
| DARS | Aspartate--tRNA ligase, cytoplasmic | P14868 | 30.9 | 30.9 | 15 | 15 | 57.136 | 451730000 | 44 | 0.0333 |
| DBN1 | Drebrin | Q16643 | 10.2 | 10.2 | 5 | 5 | 71.428 | 521390000 | 32 | 0.0405 |
| DBNL | Drebrin-like protein | Q9UJU6 | 30.7 | 4.9 | 11 | 2 | 48.207 | 1019100000 | 72 | 0.0441 |
| DCD | Dermcidin | P81605 | 29.1 | 29.1 | 4 | 4 | 11.284 | 6058200000 | 43 | 0.0397 |
| DDX39A | ATP-dependent RNA helicase DDX39A | O00148 | 18.3 | 2.1 | 8 | 1 | 49.129 | 346990000 | 35 | 0.0577 |
| DDX5 | Probable ATP-dependent RNA helicase DDX5 | P17844 | 11.4 | 5.7 | 7 | 4 | 69.147 | 209030000 | 20 | 0.0436 |
| DEFA3 | Neutrophil defensin 3 | P59666 | 19.1 | 19.1 | 2 | 2 | 10.245 | 1317200000 | 29 | 0.0752 |
| DIAPH1 | Protein diaphanous homolog 1 | A0A140T8Z0 | 19.2 | 19.2 | 24 | 24 | 140.3 | 1295600000 | 92 | 0.0594 |
| DLST | Dihydrolipoyllysine-residue succinyltransferase component of 2-oxoglutarate dehydrogenase complex, mitochondrial | P36957 | 5.7 | 5.7 | 3 | 3 | 48.755 | 148260000 | 14 | 0.0487 |
| DMTN | Dematin | Q08495 | 21.5 | 21.5 | 8 | 8 | 45.514 | 573340000 | 30 | 0.0620 |
| DNM1 | Dynamin-1 | A0A0D9SFB1 | 12.9 | 6.9 | 10 | 5 | 94.016 | 863610000 | 49 | 0.0516 |
| DNM1L | Dynamin-1-like protein | G8JLD5 | 26.7 | 26.7 | 18 | 18 | 79.621 | 1903500000 | 106 | 0.0400 |
| DPYSL2 | Dihydropyrimidinase-related protein 2 | Q16555 | 47.4 | 40.9 | 18 | 15 | 62.293 | 5487800000 | 197 | 0.0555 |
| DSC1 | Desmocollin-1 | Q08554 | 20 | 20 | 14 | 14 | 99.986 | 1967800000 | 62 | 0.0903 |
| DSG1 | Desmoglein-1 | Q02413 | 27.7 | 27.7 | 22 | 22 | 113.75 | 4330800000 | 176 | 0.0627 |
| DSP | Desmoplakin | P15924 | 45 | 45 | 131 | 131 | 331.77 | 14418000000 | 542 | 0.0887 |
| DSTN | Destrin | P60981 | 53.3 | 49.1 | 10 | 9 | 18.506 | 1414700000 | 49 | 0.0716 |
| DUSP3 | Dual specificity protein phosphatase 3 | P51452 | 38.4 | 38.4 | 6 | 6 | 20.478 | 710670000 | 45 | 0.0504 |
| DYNLL2 | Dynein light chain 2, cytoplasmic | Q96FJ2 | 12.4 | 12.4 | 1 | 1 | 10.35 | 432740000 | 19 | 0.0808 |
| ECM1 | Extracellular matrix protein 1 | Q16610 | 7.8 | 7.8 | 4 | 4 | 60.673 | 137160000 | 15 | 0.0467 |
| EEF1A1P5 | Putative elongation factor 1-alpha-like 3 | Q5VTE0 | 33.5 | 17.7 | 14 | 7 | 50.184 | 19165000000 | 225 | 0.0115 |
| EEF1B2 | Elongation factor 1-beta | P24534 | 28.4 | 23.6 | 5 | 4 | 24.763 | 328240000 | 14 | 0.0496 |
| EEF1D | Elongation factor 1-delta | P29692 | 43.4 | 39.5 | 9 | 8 | 31.121 | 1373000000 | 67 | 0.0424 |
| EEF1G | Elongation factor 1-gamma | P26641 | 30.2 | 30.2 | 14 | 14 | 50.118 | 1608900000 | 78 | 0.0252 |
| EEF2 | Elongation factor 2 | P13639 | 28.6 | 28.6 | 24 | 24 | 95.337 | 4006200000 | 209 | 0.0239 |
| EFHD2 | EF-hand domain-containing protein D2 | Q96C19 | 23.3 | 23.3 | 5 | 5 | 26.697 | 540260000 | 41 | 0.0464 |
| EHD1 | EH domain-containing protein 1 | Q9H4M9 | 29.4 | 16.5 | 14 | 7 | 60.626 | 580010000 | 30 | 0.0580 |
| EHD3 | EH domain-containing protein 3 | Q9NZN3 | 40.4 | 27.5 | 18 | 11 | 60.886 | 4379800000 | 161 | 0.0363 |
| EIF2S2 | Eukaryotic translation initiation factor 2 subunit 2 | P20042 | 6.3 | 6.3 | 2 | 2 | 38.388 | 63960000 | 6 | 0.0454 |
| EIF4A1 | Eukaryotic initiation factor 4A-I | P60842 | 35 | 2.7 | 14 | 1 | 46.153 | 2606000000 | 114 | 0.0142 |
| EIF5A | Eukaryotic translation initiation factor 5A-1 | I3L397 | 34.2 | 34.2 | 6 | 6 | 16.019 | 1478900000 | 81 | 0.0383 |
| EMILIN1 | EMILIN-1 | Q9Y6C2 | 25.1 | 25.1 | 21 | 21 | 106.69 | 3766000000 | 160 | 0.0555 |
| ENO1 | Alpha-enolase | P06733 | 59.9 | 52.3 | 22 | 20 | 47.168 | 39593000000 | 425 | 0.0106 |
| ENO2 | Gamma-enolase | P09104 | 22.6 | 13.1 | 8 | 5 | 47.268 | 1554600000 | 59 | 0.0665 |
| ERH | Enhancer of rudimentary homolog | P84090 | 27.9 | 27.9 | 3 | 3 | 12.259 | 182530000 | 14 | 0.0465 |
| ERP44 | Endoplasmic reticulum resident protein 44 | Q9BS26 | 20 | 20 | 8 | 8 | 46.971 | 1047200000 | 66 | 0.0326 |
| ESD | S-formylglutathione hydrolase | P10768 | 20.6 | 20.6 | 5 | 5 | 31.462 | 355940000 | 27 | 0.0466 |
| ETFA | Electron transfer flavoprotein subunit alpha, mitochondrial | P13804 | 23.1 | 23.1 | 7 | 7 | 35.079 | 234300000 | 26 | 0.0718 |
| ETFB | Electron transfer flavoprotein subunit beta | P38117 | 16.1 | 16.1 | 5 | 5 | 27.843 | 305380000 | 19 | 0.0491 |
| F11R | Junctional adhesion molecule A | Q9Y624 | 12 | 12 | 4 | 4 | 32.583 | 272570000 | 17 | 0.0409 |
| F13A1 | Coagulation factor XIII A chain | P00488 | 44.8 | 44.8 | 29 | 29 | 83.266 | 50581000000 | 556 | 0.0213 |
| F2 | Prothrombin | P00734 | 34.6 | 34.6 | 15 | 15 | 70.036 | 2382200000 | 99 | 0.0417 |
| F5 | Coagulation factor V | P12259 | 26.7 | 22.3 | 53 | 43 | 251.7 | 16773000000 | 522 | 0.0173 |
| FABP5 | Fatty acid-binding protein, epidermal | Q01469 | 71.9 | 71.9 | 10 | 10 | 15.164 | 4424600000 | 104 | 0.0476 |
| FCN1 | Ficolin-1 | O00602 | 21.2 | 21.2 | 6 | 6 | 35.078 | 2307600000 | 80 | 0.0355 |
| FERMT3 | Fermitin family homolog 3 | Q86UX7 | 51.6 | 51.6 | 32 | 32 | 75.952 | 40547000000 | 486 | 0.0193 |
| FGA | Fibrinogen alpha chain | P02671 | 49.4 | 48.4 | 38 | 36 | 94.972 | 5.2569E+11 | 1345 | 0.0125 |
| FGB | Fibrinogen beta chain | P02675 | 74.7 | 66.2 | 37 | 31 | 55.928 | 4.1413E+11 | 1320 | 0.0107 |
| FGG | Fibrinogen gamma chain | P02679 | 70 | 48.1 | 31 | 20 | 51.511 | 4.3328E+11 | 812 | 0.0117 |
| FHL1 | Four and a half LIM domains protein 1 | Q5JXI8 | 41.2 | 41.2 | 10 | 10 | 29.16 | 3132800000 | 101 | 0.0191 |
| FLNA | Filamin-A | P21333 | 65.5 | 62.1 | 130 | 120 | 280.74 | 1.9447E+11 | 2445 | 0.0085 |
| FN1 | Fibronectin | P02751 | 33.9 | 33.9 | 66 | 66 | 262.62 | 17581000000 | 651 | 0.0213 |
| FYB | FYN-binding protein | O15117 | 10.9 | 10.9 | 7 | 7 | 85.386 | 475720000 | 40 | 0.0368 |
| G6PD | Glucose-6-phosphate 1-dehydrogenase | P11413 | 41.2 | 41.2 | 21 | 21 | 59.256 | 1146600000 | 73 | 0.0510 |
| GANAB | Neutral alpha-glucosidase AB | Q14697 | 12.3 | 12.3 | 11 | 11 | 106.87 | 812070000 | 51 | 0.0484 |
| GAPDH | Glyceraldehyde-3-phosphate dehydrogenase | P04406 | 73.4 | 73.4 | 18 | 18 | 36.053 | 78419000000 | 463 | 0.0072 |
| GC | Vitamin D-binding protein | D6RF35 | 48.3 | 37 | 20 | 16 | 53.02 | 6526400000 | 198 | 0.0483 |
| GDI1 | Rab GDP dissociation inhibitor alpha | P31150 | 35.3 | 23 | 13 | 8 | 50.582 | 642800000 | 46 | 0.0593 |
| GDI2 | Rab GDP dissociation inhibitor beta | P50395 | 40.7 | 28.3 | 16 | 11 | 50.663 | 2255500000 | 95 | 0.0600 |
| GFAP | Glial fibrillary acidic protein | P14136 | 18.8 | 15.3 | 9 | 7 | 49.88 | 390550000 | 37 | 0.0641 |
| GGCT | Gamma-glutamylcyclotransferase | O75223 | 43.1 | 43.1 | 7 | 7 | 21.007 | 351370000 | 29 | 0.0696 |
| GLG1 | Golgi apparatus protein 1 | Q92896 | 1.8 | 1.8 | 2 | 2 | 134.55 | 24045000 | 6 | 0.0364 |
| GLO1 | Lactoylglutathione lyase | Q04760 | 22.8 | 22.8 | 4 | 4 | 20.777 | 149650000 | 12 | 0.0640 |
| GLUD1 | Glutamate dehydrogenase 1, mitochondrial | P00367 | 19.7 | 19.7 | 10 | 10 | 61.397 | 1668800000 | 67 | 0.0595 |
| GLUL | Glutamine synthetase | P15104 | 18.8 | 18.8 | 7 | 7 | 42.064 | 1345800000 | 57 | 0.0581 |
| GM2A | Ganglioside GM2 activator | P17900 | 13 | 13 | 3 | 3 | 20.838 | 145880000 | 10 | 0.0648 |
| GNAI2 | Guanine nucleotide-binding protein G(i) subunit alpha-2 | P04899 | 43.4 | 28.5 | 12 | 7 | 40.45 | 3325000000 | 103 | 0.0691 |
| GNAO1 | Guanine nucleotide-binding protein G(o) subunit alpha | P09471 | 22 | 15.5 | 7 | 5 | 40.05 | 724570000 | 33 | 0.0672 |
| GNB1 | Guanine nucleotide-binding protein G(I)/G(S)/G(T) subunit beta-1 | P62873 | 28.2 | 17.1 | 10 | 6 | 37.377 | 2843900000 | 98 | 0.0408 |
| GNB2L1 | Guanine nucleotide-binding protein subunit beta-2-like 1 | P63244 | 36 | 36 | 10 | 10 | 35.076 | 405020000 | 32 | 0.0436 |
| GOT1 | Aspartate aminotransferase, cytoplasmic | P17174 | 18.2 | 18.2 | 7 | 7 | 46.247 | 611780000 | 32 | 0.0548 |
| GOT2 | Aspartate aminotransferase, mitochondrial | P00505 | 26 | 26 | 10 | 10 | 47.517 | 1517500000 | 85 | 0.0498 |
| GP1BA | Platelet glycoprotein Ib alpha chain | A0A0C4DGZ8 | 19.8 | 19.8 | 12 | 12 | 68.954 | 9891100000 | 148 | 0.0226 |
| GP1BB | Platelet glycoprotein Ib beta chain | P13224 | 18.4 | 18.4 | 4 | 4 | 21.717 | 1140700000 | 29 | 0.0607 |
| GP5 | Platelet glycoprotein V | P40197 | 30.7 | 30.7 | 14 | 14 | 60.958 | 17898000000 | 254 | 0.0137 |
| GP9 | Platelet glycoprotein IX | P14770 | 32.2 | 32.2 | 5 | 5 | 19.046 | 1422000000 | 35 | 0.0697 |
| GPI | Glucose-6-phosphate isomerase | P06744 | 27.4 | 18.1 | 13 | 9 | 63.146 | 1834700000 | 91 | 0.0393 |
| GPX1 | Glutathione peroxidase 1 | A0A087WUQ6 | 49 | 49 | 8 | 8 | 21.938 | 1654200000 | 77 | 0.0235 |
| GRB2 | Growth factor receptor-bound protein 2 | P62993 | 33.6 | 33.6 | 7 | 7 | 25.206 | 1281500000 | 50 | 0.0645 |
| GSN | Gelsolin | P06396 | 48.5 | 20.7 | 33 | 10 | 85.696 | 37412000000 | 452 | 0.0116 |
| GSN | Gelsolin | Q5T0I0 | 34.6 | 3.5 | 9 | 1 | 28.953 | 2520500000 | 25 | 0.0946 |
| GSPT1 | Eukaryotic peptide chain release factor GTP-binding subunit ERF3A | H3BR35 | 7.8 | 7.8 | 4 | 4 | 52.832 | 129720000 | 11 | 0.0420 |
| GSR | Glutathione reductase, mitochondrial | P00390 | 10.3 | 10.3 | 5 | 5 | 56.256 | 431250000 | 28 | 0.0557 |
| GSTO1 | Glutathione S-transferase omega-1 | P78417 | 44 | 44 | 11 | 11 | 27.566 | 5210900000 | 137 | 0.0141 |
| GSTP1 | Glutathione S-transferase P | P09211 | 69 | 69 | 11 | 11 | 23.356 | 3761600000 | 100 | 0.0549 |
| H1F0 | Histone H1.0 | P07305 | 27.3 | 27.3 | 5 | 5 | 20.863 | 927100000 | 32 | 0.0970 |
| H2AFV | Histone H2A.V | Q71UI9 | 53.9 | 46.9 | 6 | 4 | 13.509 | 648050000 | 26 | 0.0712 |
| H2AFY | Core histone macro-H2A.1 | O75367 | 15.1 | 15.1 | 5 | 5 | 39.617 | 208050000 | 13 | 0.0575 |
| HBA1 | Hemoglobin subunit alpha | P69905 | 71.8 | 18.3 | 10 | 3 | 15.257 | 68904000000 | 267 | 0.0131 |
| HBB | Hemoglobin subunit beta | P68871 | 93.9 | 55.8 | 12 | 6 | 15.998 | 1.1111E+11 | 396 | 0.0130 |
| HBD | Hemoglobin subunit delta | P02042 | 68 | 29.9 | 9 | 3 | 16.055 | 549410000 | 21 | 0.0617 |
| HCLS1 | Hematopoietic lineage cell-specific protein | P14317 | 16.9 | 16.9 | 8 | 8 | 54.013 | 445540000 | 22 | 0.0546 |
| HINT1 | Histidine triad nucleotide-binding protein 1 | P49773 | 19.8 | 19.8 | 3 | 3 | 13.802 | 610220000 | 27 | 0.0517 |
| HIST1H1B | Histone H1.5 | P16401 | 31 | 22.1 | 9 | 6 | 22.58 | 4804600000 | 71 | 0.0149 |
| HIST1H1E | Histone H1.4 | P10412 | 37.9 | 11.4 | 13 | 3 | 21.865 | 33064000000 | 167 | 0.0108 |
| HIST1H2AC | Histone H2A type 1-C | Q93077 | 35.4 | 8.5 | 6 | 1 | 14.105 | 342350000 | 12 | 0.0611 |
| HIST1H2BC | Histone H2B type 1-C/E/F/G/I | P62807 | 67.5 | 0 | 10 | 0 | 13.906 | 52317000000 | 190 | 0.0240 |
| HIST1H4A | Histone H4 | P62805 | 59.2 | 59.2 | 13 | 13 | 11.367 | 77095000000 | 263 | 0.0342 |
| HIST2H2AC | Histone H2A type 2-C | Q16777 | 58.1 | 31 | 7 | 2 | 13.988 | 36886000000 | 117 | 0.0615 |
| HIST2H2BE | Histone H2B type 2-E | Q16778 | 67.5 | 0 | 10 | 0 | 13.92 | 2935700000 | 24 | 0.1094 |
| HIST2H3A | Histone H3.2 | Q71DI3 | 26.5 | 8.1 | 5 | 2 | 15.388 | 36440000000 | 84 | 0.0343 |
| HIST2H3PS2 | Histone H3 | Q5TEC6 | 26.5 | 8.1 | 4 | 1 | 15.43 | 2030400000 | 22 | 0.1025 |
| HK1 | Hexokinase-1 | P19367 | 13.1 | 11.8 | 12 | 11 | 102.48 | 927370000 | 67 | 0.0502 |
| HLA-A | HLA class I histocompatibility antigen, A-34 alpha chain | P30453 | 34.5 | 0 | 11 | 0 | 41.054 | 1820300000 | 81 | 0.0700 |
| HLA-B | HLA class I histocompatibility antigen, B-27 alpha chain | P03989 | 35.1 | 10.8 | 10 | 2 | 40.428 | 819370000 | 20 | 0.0814 |
| HMGB1 | High mobility group protein B1 | Q5T7C4 | 34.8 | 34.8 | 6 | 6 | 18.311 | 738490000 | 28 | 0.0633 |
| HMGB2 | High mobility group protein B2 | P26583 | 11.5 | 11.5 | 2 | 2 | 24.033 | 511520000 | 26 | 0.0515 |
| HMGN2 | Non-histone chromosomal protein HMG-17 | P05204 | 27.8 | 27.8 | 3 | 3 | 9.3926 | 484530000 | 21 | 0.0540 |
| HMHA1 | Minor histocompatibility protein HA-1 | Q92619 | 9 | 9 | 10 | 10 | 124.61 | 217330000 | 17 | 0.0400 |
| HNRNPA1 | Heterogeneous nuclear ribonucleoprotein A1 | F8W6I7 | 48.5 | 43.3 | 12 | 10 | 33.155 | 1751200000 | 87 | 0.0471 |
| HNRNPA2B1 | Heterogeneous nuclear ribonucleoproteins A2/B1 | P22626 | 50.1 | 47.6 | 15 | 14 | 37.429 | 3722500000 | 152 | 0.0222 |
| HNRNPA3 | Heterogeneous nuclear ribonucleoprotein A3 | P51991 | 34.7 | 32.8 | 10 | 9 | 39.594 | 778220000 | 42 | 0.0563 |
| HNRNPAB | Heterogeneous nuclear ribonucleoprotein A/B | D6R9P3 | 12.5 | 12.5 | 3 | 3 | 30.302 | 253800000 | 24 | 0.0350 |
| HNRNPC | Heterogeneous nuclear ribonucleoproteins C1/C2 | G3V4W0 | 34.7 | 34.7 | 12 | 12 | 28.916 | 1171100000 | 76 | 0.0377 |
| HNRNPD | Heterogeneous nuclear ribonucleoprotein D0 | Q14103 | 21.7 | 19.4 | 8 | 7 | 38.434 | 1437200000 | 69 | 0.0510 |
| HNRNPH1 | Heterogeneous nuclear ribonucleoprotein H | E9PCY7 | 18.2 | 15.9 | 6 | 5 | 47.087 | 1070600000 | 52 | 0.0555 |
| HNRNPK | Heterogeneous nuclear ribonucleoprotein K | P61978 | 43.8 | 43.8 | 17 | 17 | 50.976 | 2685400000 | 139 | 0.0218 |
| HNRNPU | Heterogeneous nuclear ribonucleoprotein U | A0A1W2PP35 | 15.7 | 15.7 | 11 | 11 | 80.648 | 1135900000 | 77 | 0.0476 |
| HP | Haptoglobin | P00738 | 48.3 | 24.6 | 23 | 9 | 45.205 | 35626000000 | 375 | 0.0261 |
| HPSE | Heparanase | Q9Y251 | 11 | 11 | 6 | 6 | 61.148 | 554840000 | 32 | 0.0529 |
| HPX | Hemopexin | P02790 | 45.2 | 45.2 | 15 | 15 | 51.676 | 9351800000 | 158 | 0.0221 |
| HRG | Histidine-rich glycoprotein | P04196 | 32.4 | 32.4 | 15 | 15 | 59.578 | 2061400000 | 85 | 0.0744 |
| HSP90AA1 | Heat shock protein HSP 90-alpha | P07900 | 40.4 | 22.3 | 30 | 16 | 84.659 | 7445200000 | 194 | 0.0128 |
| HSP90AB1 | Heat shock protein HSP 90-beta | P08238 | 45.7 | 23.3 | 33 | 15 | 83.263 | 20789000000 | 410 | 0.0139 |
| HSP90B1 | Endoplasmin | P14625 | 38.2 | 36.5 | 29 | 27 | 92.468 | 6170600000 | 221 | 0.0152 |
| HSPA1B | Heat shock 70 kDa protein 1B | P0DMV9 | 35.7 | 14 | 21 | 7 | 70.051 | 3592200000 | 136 | 0.0130 |
| HSPA4 | Heat shock 70 kDa protein 4 | P34932 | 12.1 | 12.1 | 9 | 9 | 94.33 | 273630000 | 23 | 0.0555 |
| HSPA5 | 78 kDa glucose-regulated protein | P11021 | 42.5 | 41.4 | 27 | 25 | 72.332 | 7008700000 | 255 | 0.0143 |
| HSPA8 | Heat shock cognate 71 kDa protein | P11142 | 48.6 | 25.9 | 31 | 16 | 70.897 | 26545000000 | 521 | 0.0135 |
| HSPA9 | Stress-70 protein, mitochondrial | P38646 | 19.9 | 18.9 | 12 | 11 | 73.68 | 571510000 | 56 | 0.0515 |
| HSPB1 | Heat shock protein beta-1 | P04792 | 56.1 | 56.1 | 10 | 10 | 22.782 | 3986500000 | 116 | 0.0241 |
| HSPD1 | 60 kDa heat shock protein, mitochondrial | P10809 | 38.9 | 38.9 | 20 | 20 | 61.054 | 3628100000 | 148 | 0.0653 |
| HSPE1 | 10 kDa heat shock protein, mitochondrial | P61604 | 67.6 | 67.6 | 7 | 7 | 10.932 | 1137000000 | 48 | 0.0700 |
| IDH1 | Isocitrate dehydrogenase [NADP] cytoplasmic | O75874 | 27.1 | 24.9 | 11 | 10 | 46.659 | 1298600000 | 70 | 0.0526 |
| IDH3A | Isocitrate dehydrogenase [NAD] subunit alpha, mitochondrial | P50213 | 17.8 | 17.8 | 6 | 6 | 39.591 | 313590000 | 25 | 0.0604 |
| IGHA1 | Ig alpha-1 chain C region | P01876 | 45.3 | 25.2 | 12 | 5 | 37.654 | 31949000000 | 215 | 0.0249 |
| IGHG1 | Ig gamma-1 chain C region | P01857 | 61.8 | 31.5 | 15 | 7 | 36.105 | 1.4983E+11 | 438 | 0.0153 |
| IGHG2 | Ig gamma-2 chain C region | P01859 | 55.8 | 30.7 | 12 | 5 | 35.9 | 23693000000 | 128 | 0.0168 |
| IGHG3 | Ig gamma-3 chain C region | P01860 | 52.3 | 30.2 | 14 | 6 | 41.287 | 8935800000 | 124 | 0.0235 |
| IGHG4 | Ig gamma-4 chain C region | P01861 | 43.7 | 19 | 10 | 4 | 35.94 | 2196300000 | 49 | 0.0968 |
| IGHM | Ig mu chain C region | P01871 | 36.6 | 36.6 | 15 | 15 | 49.439 | 13624000000 | 265 | 0.0144 |
| IGHV1-2 | Ig heavy chain V-I region V35 | P23083 | 9.4 | 9.4 | 1 | 1 | 13.085 | 118980000 | 10 | 0.0284 |
| IGHV3-7 | Ig heavy chain V-III region JON | P01780 | 32.5 | 13.7 | 4 | 2 | 12.943 | 1365600000 | 37 | 0.0767 |
| IGHV3OR16-12 | Immunoglobulin heavy variable 3/OR16-12 | A0A075B7B8 | 18.8 | 9.4 | 2 | 1 | 12.874 | 307090000 | 13 | 0.0439 |
| IGHV3OR16-9 | Immunoglobulin heavy variable 3/OR16-9 | S4R460 | 31.2 | 19.8 | 2 | 1 | 10.429 | 1807400000 | 49 | 0.0270 |
| IGJ | Immunoglobulin J chain | D6RD17 | 33.1 | 33.1 | 5 | 5 | 18.02 | 1532800000 | 51 | 0.0606 |
| IGKC | Ig kappa chain C region | P01834 | 82.2 | 82.2 | 8 | 8 | 11.765 | 61346000000 | 279 | 0.0147 |
| IGKV2-24 | Immunoglobulin kappa variable 2-24 | A0A0C4DH68 | 16.7 | 10.8 | 2 | 1 | 13.079 | 478520000 | 13 | 0.0645 |
| IGKV2-4 | Ig kappa chain V-II region FR | A0A087X0Q4 | 19.2 | 6.7 | 2 | 1 | 11.434 | 1955800000 | 30 | 0.0795 |
| IGKV3-15 | Ig kappa chain V-III region POM | P01624 | 13.9 | 7.8 | 2 | 1 | 12.496 | 2001700000 | 37 | 0.0678 |
| IGKV3-20 | Ig kappa chain V-III region B6 | P01619 | 27.6 | 7.8 | 3 | 1 | 12.557 | 2547300000 | 40 | 0.0744 |
| IGLC6 | Ig lambda-6 chain C region | P0DOY3 | 69.8 | 34 | 6 | 3 | 11.265 | 6838800000 | 127 | 0.0519 |
| IGLL5 | Immunoglobulin lambda-like polypeptide 5 | B9A064 | 35 | 17.3 | 6 | 3 | 23.063 | 30021000000 | 146 | 0.0156 |
| IGLV3-21 | Ig lambda chain V-III region LOI | P80748 | 29.9 | 29.9 | 3 | 3 | 12.446 | 547080000 | 28 | 0.0555 |
| IGLV4-69 | Immunoglobulin lambda variable 4-69 | A0A075B6H9 | 8.4 | 8.4 | 1 | 1 | 12.773 | 13393000 | 1 | 0.0423 |
| ILK | Integrin-linked protein kinase | Q13418 | 53.1 | 53.1 | 22 | 22 | 51.419 | 14943000000 | 360 | 0.0163 |
| INA | Alpha-internexin | Q16352 | 10.6 | 5.4 | 6 | 3 | 55.39 | 165160000 | 13 | 0.0512 |
| IQGAP1 | Ras GTPase-activating-like protein IQGAP1 | P46940 | 10.1 | 9.2 | 16 | 14 | 189.25 | 376660000 | 41 | 0.0434 |
| ITGA2B | Integrin alpha-IIb | P08514 | 33.8 | 33.8 | 28 | 28 | 113.38 | 35374000000 | 382 | 0.0328 |
| ITGA6 | Integrin alpha-6 | P23229 | 15.3 | 15.3 | 16 | 16 | 126.6 | 1162100000 | 61 | 0.0649 |
| ITGAM | Integrin alpha-M | P11215 | 3.2 | 3.2 | 4 | 4 | 127.18 | 98771000 | 12 | 0.0406 |
| ITGB1 | Integrin beta-1 | P05556 | 13.7 | 13.7 | 10 | 10 | 88.414 | 716980000 | 43 | 0.0565 |
| ITGB3 | Integrin beta-3 | P05106 | 38.3 | 38.3 | 26 | 26 | 87.057 | 20005000000 | 349 | 0.0818 |
| ITGB6 | Integrin beta | A0A087WXP3 | 1.3 | 1.3 | 1 | 1 | 75.849 | 247100000 | 9 | 0.0432 |
| ITIH1 | Inter-alpha-trypsin inhibitor heavy chain H1 | P19827 | 20.9 | 20.9 | 14 | 14 | 101.39 | 1833300000 | 98 | 0.0443 |
| ITIH2 | Inter-alpha-trypsin inhibitor heavy chain H2 | Q5T985 | 25.3 | 19 | 22 | 15 | 105.21 | 4512400000 | 164 | 0.0155 |
| ITIH3 | Inter-alpha-trypsin inhibitor heavy chain H3 | A0A087WW43 | 4.8 | 4.8 | 3 | 3 | 75.077 | 43497000 | 4 | 0.0371 |
| ITIH4 | Inter-alpha-trypsin inhibitor heavy chain H4 | Q14624 | 31.3 | 10.5 | 26 | 7 | 103.36 | 6633900000 | 225 | 0.0259 |
| IVL | Involucrin | P07476 | 15.6 | 15.6 | 8 | 8 | 68.478 | 113630000 | 13 | 0.0564 |
| JUP | Junction plakoglobin | P14923 | 53 | 53 | 32 | 32 | 81.744 | 6008700000 | 195 | 0.0759 |
| KIF2A | Kinesin-like protein KIF2A | O00139 | 11.9 | 11.9 | 9 | 9 | 79.954 | 260240000 | 25 | 0.0362 |
| KNG1 | Kininogen-1 | P01042 | 24.4 | 24.4 | 15 | 15 | 71.957 | 4760500000 | 152 | 0.0138 |
| KPNB1 | Importin subunit beta-1 | Q14974 | 6.8 | 6.8 | 5 | 5 | 97.169 | 366800000 | 31 | 0.0385 |
| KPRP | Keratinocyte proline-rich protein | Q5T749 | 49.6 | 49.6 | 21 | 21 | 64.135 | 4430700000 | 189 | 0.0627 |
| KRT1 | Keratin, type II cytoskeletal 1 | P04264 | 71.3 | 7.6 | 60 | 5 | 66.038 | 9.272E+11 | 1610 | 0.0271 |
| KRT18 | Keratin, type I cytoskeletal 18 | P05783 | 29.3 | 14.7 | 14 | 6 | 48.057 | 795480000 | 70 | 0.0360 |
| KRT2 | Keratin, type II cytoskeletal 2 epidermal | P35908 | 82.3 | 4.7 | 50 | 1 | 65.432 | 1.2215E+11 | 799 | 0.0309 |
| KRT6B | Keratin, type II cytoskeletal 6B | P04259 | 56.7 | 1.6 | 44 | 1 | 60.066 | 1200400000 | 28 | 0.0798 |
| LACRT | Extracellular glycoprotein lacritin | Q9GZZ8 | 33.3 | 33.3 | 7 | 7 | 14.246 | 1486400000 | 22 | 0.0959 |
| LAP3 | Cytosol aminopeptidase | P28838 | 10.8 | 10.8 | 5 | 5 | 56.166 | 196350000 | 20 | 0.0486 |
| LASP1 | LIM and SH3 domain protein 1 | Q14847 | 51 | 51 | 13 | 13 | 29.717 | 3274700000 | 151 | 0.0278 |
| LCN1 | Lipocalin-1 | P31025 | 21 | 21 | 4 | 4 | 19.25 | 1234200000 | 27 | 0.0989 |
| LCP1 | Plastin-2 | P13796 | 51.2 | 41 | 28 | 21 | 70.288 | 6137700000 | 241 | 0.0127 |
| LDHA | L-lactate dehydrogenase A chain | P00338 | 36.4 | 32.8 | 13 | 12 | 36.688 | 11155000000 | 181 | 0.0136 |
| LDHB | L-lactate dehydrogenase B chain | P07195 | 37.4 | 33.8 | 12 | 11 | 36.638 | 14324000000 | 245 | 0.0167 |
| LGALS1 | Galectin-1 | P09382 | 70.4 | 70.4 | 8 | 8 | 14.716 | 6093600000 | 129 | 0.0155 |
| LGALS7 | Galectin-7 | P47929 | 77.9 | 77.9 | 9 | 9 | 15.075 | 867770000 | 23 | 0.0898 |
| LGALSL | Galectin-related protein | Q3ZCW2 | 41.3 | 41.3 | 6 | 6 | 18.986 | 2343000000 | 59 | 0.0255 |
| LIMS1 | LIM and senescent cell antigen-like-containing domain protein 1 | P48059 | 48.6 | 13.5 | 14 | 5 | 37.251 | 11613000000 | 209 | 0.0123 |
| LMNA | Prelamin-A/C | P02545 | 38.3 | 37 | 24 | 23 | 74.139 | 1819000000 | 109 | 0.0674 |
| LMNB1 | Lamin-B1 | P20700 | 17.1 | 15.7 | 10 | 9 | 66.408 | 546660000 | 44 | 0.0450 |
| LTBP1 | Latent-transforming growth factor beta-binding protein 1 | Q14766 | 38.4 | 6.9 | 50 | 12 | 186.79 | 46645000000 | 725 | 0.0175 |
| LTF | Lactotransferrin | E7EQB2 | 46.1 | 43.1 | 27 | 25 | 76.625 | 2454800000 | 101 | 0.0915 |
| LYZ | Lysozyme C | P61626 | 61.5 | 61.5 | 8 | 8 | 16.537 | 17375000000 | 134 | 0.0204 |
| M6PR | Cation-dependent mannose-6-phosphate receptor | P20645 | 15.2 | 15.2 | 4 | 4 | 30.993 | 349480000 | 21 | 0.0505 |
| MAP1B | Microtubule-associated protein 1B | P46821 | 0.8 | 0.8 | 2 | 2 | 270.63 | 36068000 | 3 | 0.0383 |
| MAP2 | Microtubule-associated protein 2 | P11137 | 1.1 | 1.1 | 2 | 2 | 199.52 | 7597400 | 1 | 0.0430 |
| MAPRE1 | Microtubule-associated protein RP/EB family member 1 | Q15691 | 33.2 | 33.2 | 8 | 8 | 29.999 | 984240000 | 44 | 0.0707 |
| MAPRE2 | Microtubule-associated protein RP/EB family member 2 | K7EL66 | 17.9 | 17.9 | 3 | 3 | 15.035 | 503380000 | 23 | 0.0557 |
| MAT1A | S-adenosylmethionine synthase isoform type-1 | Q00266 | 7.6 | 7.6 | 2 | 2 | 43.647 | 353620000 | 18 | 0.0433 |
| MBP | Myelin basic protein | J3QL64 | 28 | 5.1 | 5 | 1 | 17.119 | 25240000000 | 129 | 0.0868 |
| MDH1 | Malate dehydrogenase, cytoplasmic | P40925 | 23.4 | 23.4 | 7 | 7 | 36.426 | 3215300000 | 87 | 0.0389 |
| MDH2 | Malate dehydrogenase, mitochondrial | P40926 | 40.5 | 40.5 | 12 | 12 | 35.503 | 3287300000 | 103 | 0.0620 |
| MIF | Macrophage migration inhibitory factor | P14174 | 23.5 | 23.5 | 3 | 3 | 12.476 | 1171300000 | 25 | 0.0755 |
| MMRN1 | Multimerin-1 | Q13201 | 37.6 | 37.6 | 45 | 45 | 138.11 | 57467000000 | 802 | 0.0167 |
| MNDA | Myeloid cell nuclear differentiation antigen | P41218 | 18.9 | 18.9 | 8 | 8 | 45.836 | 622060000 | 40 | 0.0404 |
| MPIG6B | Megakaryocyte and platelet inhibitory receptor G6b | A0A140T9L8 | 26.6 | 5.9 | 5 | 1 | 24.903 | 420700000 | 25 | 0.0562 |
| MPO | Myeloperoxidase | P05164 | 34 | 34 | 24 | 24 | 83.868 | 19994000000 | 218 | 0.0503 |
| MPZ | Myelin protein P0 | P25189 | 19.4 | 19.4 | 5 | 5 | 27.554 | 2831500000 | 43 | 0.0944 |
| MSN | Moesin | P26038 | 37.8 | 25.6 | 27 | 18 | 67.819 | 10406000000 | 338 | 0.0117 |
| MTPN | Myotrophin | P58546 | 37.3 | 37.3 | 4 | 4 | 12.895 | 1007000000 | 52 | 0.0418 |
| MYH14 | Myosin-14 | Q7Z406 | 4.7 | 1 | 11 | 2 | 227.87 | 2561500000 | 40 | 0.0698 |
| MYH9 | Myosin-9 | P35579 | 56.2 | 52.4 | 113 | 104 | 226.53 | 1.0956E+11 | 1852 | 0.0131 |
| MYL1 | Myosin light chain 1/3, skeletal muscle isoform | P05976 | 22.7 | 22.7 | 5 | 5 | 21.145 | 7364000000 | 84 | 0.0633 |
| MYL12A | Myosin regulatory light chain 12A | P19105 | 62.6 | 24 | 9 | 3 | 19.794 | 5317900000 | 43 | 0.0857 |
| MYL6 | Myosin light polypeptide 6 | F8W1R7 | 62.8 | 62.8 | 9 | 9 | 16.29 | 22728000000 | 159 | 0.0141 |
| MYL9 | Myosin regulatory light polypeptide 9 | P24844 | 62.2 | 23.8 | 9 | 3 | 19.827 | 10137000000 | 148 | 0.0152 |
| MYLK | Myosin light chain kinase, smooth muscle | Q15746 | 5.9 | 5.9 | 11 | 11 | 210.71 | 901530000 | 55 | 0.0511 |
| MYLPF | Myosin regulatory light chain 2, skeletal muscle isoform | H3BML9 | 47.5 | 47.5 | 7 | 7 | 13.152 | 1085300000 | 38 | 0.0809 |
| NAP1L1 | Nucleosome assembly protein 1-like 1 | F8W118 | 36.4 | 31.6 | 6 | 5 | 24.694 | 1588000000 | 46 | 0.0663 |
| NAPA | Alpha-soluble NSF attachment protein | P54920 | 35.6 | 35.6 | 9 | 9 | 33.232 | 520470000 | 36 | 0.0432 |
| NARS | Asparagine--tRNA ligase, cytoplasmic | O43776 | 5.5 | 5.5 | 3 | 3 | 62.942 | 9971900 | 2 | 0.0401 |
| NCL | Nucleolin | P19338 | 22.4 | 22.4 | 17 | 17 | 76.613 | 2008900000 | 124 | 0.0526 |
| NEFH | Neurofilament heavy polypeptide | P12036 | 5.9 | 4.2 | 7 | 4 | 112.48 | 64239000 | 10 | 0.0387 |
| NEFL | Neurofilament light polypeptide | P07196 | 22.8 | 19.7 | 13 | 11 | 61.516 | 1373000000 | 68 | 0.0582 |
| NEFM | Neurofilament medium polypeptide | E7EMV2 | 20.2 | 17.8 | 14 | 11 | 78.881 | 657660000 | 52 | 0.0779 |
| NEXN | Nexilin | Q0ZGT2 | 8.4 | 8.4 | 6 | 6 | 80.657 | 149660000 | 13 | 0.0463 |
| NID1 | Nidogen-1 | P14543 | 25.7 | 24.9 | 28 | 27 | 136.38 | 6180800000 | 247 | 0.0305 |
| NME1-NME2 | Nucleoside diphosphate kinase | Q32Q12 | 55.5 | 2.4 | 13 | 1 | 32.642 | 5498700000 | 154 | 0.0184 |
| NPM1 | Nucleophosmin | P06748 | 24.8 | 24.8 | 7 | 7 | 32.575 | 2355000000 | 67 | 0.0495 |
| NRGN | Neurogranin | Q92686 | 19.2 | 19.2 | 2 | 2 | 7.6184 | 226910000 | 15 | 0.0424 |
| NSF | Vesicle-fusing ATPase | I3L0N3 | 24 | 24 | 16 | 16 | 82.091 | 913150000 | 65 | 0.0432 |
| OLFML2A | Olfactomedin-like protein 2A | Q68BL7 | 3.7 | 3.7 | 3 | 3 | 73.054 | 23049000 | 3 | 0.0384 |
| ORM1 | Alpha-1-acid glycoprotein 1 | P02763 | 40.3 | 32.3 | 7 | 5 | 23.511 | 5407100000 | 102 | 0.0372 |
| ORM2 | Alpha-1-acid glycoprotein 2 | P19652 | 25.9 | 17.9 | 6 | 4 | 23.602 | 391400000 | 16 | 0.0445 |
| P4HB | Protein disulfide-isomerase | P07237 | 40.9 | 40.9 | 22 | 22 | 57.116 | 3767200000 | 187 | 0.0424 |
| PA2G4 | Proliferation-associated protein 2G4 | Q9UQ80 | 23.4 | 23.4 | 9 | 9 | 43.786 | 627330000 | 57 | 0.0395 |
| PABPC1 | Polyadenylate-binding protein 1 | A0A087WTT1 | 20.5 | 20.5 | 10 | 10 | 58.535 | 298990000 | 29 | 0.0496 |
| PAK2 | Serine/threonine-protein kinase PAK 2 | Q13177 | 5.5 | 5.5 | 3 | 3 | 58.042 | 416900000 | 18 | 0.0360 |
| PARK7 | Protein DJ-1 | Q99497 | 53.4 | 53.4 | 9 | 9 | 19.891 | 2514200000 | 95 | 0.0234 |
| PARVB | Beta-parvin | Q9HBI1 | 27.2 | 27.2 | 9 | 9 | 41.714 | 12361000000 | 172 | 0.0180 |
| PCBP1 | Poly(rC)-binding protein 1 | Q15365 | 45.5 | 30.1 | 12 | 7 | 37.497 | 2345900000 | 85 | 0.0103 |
| PCBP2 | Poly(rC)-binding protein 2 | H3BRU6 | 30.9 | 12.6 | 8 | 3 | 31.6 | 483250000 | 32 | 0.0475 |
| PCSK6 | Proprotein convertase subtilisin/kexin type 6 | H0Y3Q0 | 6.8 | 6.8 | 5 | 5 | 88.555 | 412410000 | 28 | 0.0485 |
| PDCD10 | Programmed cell death protein 10 | Q9BUL8 | 21.2 | 21.2 | 4 | 4 | 24.701 | 237190000 | 12 | 0.0413 |
| PDE5A | cGMP-specific 3,5-cyclic phosphodiesterase | G5E9C5 | 14.2 | 14.2 | 11 | 11 | 93.629 | 445080000 | 30 | 0.0365 |
| PDIA3 | Protein disulfide-isomerase A3 | P30101 | 40.8 | 33.9 | 19 | 16 | 56.782 | 4816000000 | 171 | 0.0155 |
| PDIA3 | Thioredoxin | H7BZJ3 | 39.8 | 11.4 | 4 | 1 | 13.519 | 69683000 | 2 | 0.0400 |
| PDIA4 | Protein disulfide-isomerase A4 | P13667 | 11.2 | 11.2 | 7 | 7 | 72.932 | 165470000 | 17 | 0.0356 |
| PDIA6 | Protein disulfide-isomerase A6 | Q15084 | 26.4 | 26.4 | 10 | 10 | 48.121 | 2007100000 | 105 | 0.0149 |
| PDLIM1 | PDZ and LIM domain protein 1 | O00151 | 72.9 | 72.9 | 20 | 20 | 36.071 | 22672000000 | 338 | 0.0186 |
| PDLIM5 | PDZ and LIM domain protein 5 | Q96HC4 | 9.6 | 9.6 | 5 | 5 | 63.944 | 333510000 | 21 | 0.0487 |
| PDLIM7 | PDZ and LIM domain protein 7 | Q9NR12 | 22.5 | 22.5 | 10 | 10 | 49.844 | 822890000 | 35 | 0.0757 |
| PEBP1 | Phosphatidylethanolamine-binding protein 1 | P30086 | 39.6 | 39.6 | 6 | 6 | 21.057 | 586560000 | 27 | 0.0787 |
| PECAM1 | Platelet endothelial cell adhesion molecule | P16284 | 15.6 | 15.6 | 11 | 11 | 82.535 | 573070000 | 40 | 0.0524 |
| PF4 | Platelet factor 4 | P02776 | 44.6 | 12.9 | 6 | 3 | 10.845 | 3.9709E+11 | 523 | 0.0132 |
| PF4V1 | Platelet factor 4 variant | P10720 | 56.7 | 26 | 6 | 3 | 11.553 | 3021500000 | 49 | 0.0954 |
| PFKL | 6-phosphofructokinase, liver type | P17858 | 17.2 | 11.3 | 12 | 8 | 85.018 | 764590000 | 36 | 0.0695 |
| PFKP | 6-phosphofructokinase type C | Q01813 | 31.8 | 27 | 20 | 17 | 85.595 | 1948600000 | 113 | 0.0326 |
| PFN1 | Profilin-1 | P07737 | 69.3 | 48.6 | 8 | 5 | 15.054 | 47696000000 | 290 | 0.0107 |
| PGAM1 | Phosphoglycerate mutase 1 | P18669 | 56.3 | 56.3 | 11 | 11 | 28.804 | 2555400000 | 108 | 0.0342 |
| PGD | 6-phosphogluconate dehydrogenase, decarboxylating | P52209 | 30.2 | 30.2 | 13 | 13 | 53.139 | 2161700000 | 104 | 0.0383 |
| PGK1 | Phosphoglycerate kinase 1 | P00558 | 58.8 | 58.8 | 22 | 22 | 44.614 | 15546000000 | 316 | 0.0128 |
| PGM1 | Phosphoglucomutase-1 | P36871 | 40.9 | 40.9 | 21 | 21 | 61.448 | 1457800000 | 101 | 0.0627 |
| PHGDH | D-3-phosphoglycerate dehydrogenase | A0A286YF22 | 17.1 | 17.1 | 7 | 7 | 55.938 | 333590000 | 23 | 0.0487 |
| PI3 | Elafin | P19957 | 15.4 | 15.4 | 2 | 2 | 12.269 | 42220000 | 4 | 0.0508 |
| PIP | Prolactin-inducible protein | P12273 | 55.5 | 55.5 | 6 | 6 | 16.572 | 2003300000 | 56 | 0.0902 |
| PIP4K2A | Phosphatidylinositol 5-phosphate 4-kinase type-2 alpha | P48426 | 19.2 | 10.8 | 7 | 4 | 46.224 | 794840000 | 42 | 0.0588 |
| PKM | Pyruvate kinase PKM | P14618 | 68 | 9.2 | 36 | 3 | 57.936 | 53065000000 | 620 | 0.0074 |
| PKP1 | Plakophilin-1 | Q13835 | 19.4 | 19.4 | 13 | 13 | 82.86 | 585690000 | 27 | 0.0775 |
| PLEK | Pleckstrin | P08567 | 42.3 | 42.3 | 10 | 10 | 40.124 | 10127000000 | 151 | 0.0115 |
| PLG | Plasminogen | P00747 | 41 | 41 | 29 | 29 | 90.568 | 6046100000 | 241 | 0.0153 |
| PLP1 | Myelin proteolipid protein | P60201 | 26.7 | 26.7 | 7 | 7 | 30.077 | 23842000000 | 81 | 0.0861 |
| PNP | Purine nucleoside phosphorylase | P00491 | 56.1 | 56.1 | 13 | 13 | 32.118 | 1605000000 | 92 | 0.0395 |
| PON1 | Serum paraoxonase/arylesterase 1 | P27169 | 29.3 | 29.3 | 9 | 9 | 39.731 | 392190000 | 31 | 0.0738 |
| PPBP | Platelet basic protein | P02775 | 51.6 | 51.6 | 8 | 8 | 13.894 | 3.078E+11 | 481 | 0.0279 |
| PPIA | Peptidyl-prolyl cis-trans isomerase A | P62937 | 60 | 60 | 11 | 11 | 18.012 | 20692000000 | 232 | 0.0120 |
| PPIB | Peptidyl-prolyl cis-trans isomerase B | P23284 | 47.7 | 47.7 | 10 | 10 | 23.742 | 3548700000 | 100 | 0.0174 |
| PPM1A | Protein phosphatase 1A | P35813 | 11.8 | 11.8 | 4 | 4 | 42.447 | 73953000 | 5 | 0.0405 |
| PPP1CA | Serine/threonine-protein phosphatase PP1-alpha catalytic subunit | P62136 | 35.2 | 15.8 | 11 | 5 | 37.512 | 590000000 | 38 | 0.0499 |
| PPP1R12A | Protein phosphatase 1 regulatory subunit 12A | O14974 | 5.1 | 2.9 | 5 | 3 | 115.28 | 98007000 | 16 | 0.0384 |
| PPP2R1A | Serine/threonine-protein phosphatase 2A 65 kDa regulatory subunit A alpha isoform | P30153 | 23.4 | 23.4 | 13 | 13 | 65.308 | 482220000 | 45 | 0.0415 |
| PPP3CA | Serine/threonine-protein phosphatase 2B catalytic subunit alpha isoform | Q08209 | 13.4 | 13.4 | 7 | 7 | 58.687 | 340460000 | 31 | 0.0623 |
| PRDX1 | Peroxiredoxin-1 | Q06830 | 69.3 | 54.3 | 13 | 9 | 22.11 | 8960200000 | 167 | 0.0139 |
| PRDX2 | Peroxiredoxin-2 | P32119 | 35.9 | 30.3 | 7 | 6 | 21.892 | 1043900000 | 36 | 0.0940 |
| PRDX3 | Thioredoxin-dependent peroxide reductase, mitochondrial | P30048 | 7 | 7 | 2 | 2 | 27.692 | 279760000 | 20 | 0.0387 |
| PRDX5 | Peroxiredoxin-5, mitochondrial | P30044 | 36.4 | 36.4 | 6 | 6 | 22.086 | 3674800000 | 75 | 0.0148 |
| PRDX6 | Peroxiredoxin-6 | P30041 | 63.8 | 63.8 | 13 | 13 | 25.035 | 16775000000 | 187 | 0.0106 |
| PRH1 | Salivary acidic proline-rich phosphoprotein 1/2 | A0A087WYF5 | 30.7 | 30.7 | 3 | 3 | 14.001 | 195600000 | 17 | 0.0456 |
| PRKACB | cAMP-dependent protein kinase catalytic subunit beta | A0A087WVC4 | 13.6 | 13.6 | 5 | 5 | 39.236 | 419630000 | 22 | 0.0358 |
| PRKAR1A | cAMP-dependent protein kinase type I-alpha regulatory subunit | P10644 | 14.2 | 14.2 | 5 | 5 | 42.981 | 340200000 | 17 | 0.0559 |
| PRKCSH | Glucosidase 2 subunit beta | P14314 | 16.1 | 16.1 | 8 | 8 | 59.425 | 283360000 | 25 | 0.0509 |
| PROS1 | Vitamin K-dependent protein S | P07225 | 29.4 | 29.4 | 19 | 19 | 75.122 | 6454600000 | 165 | 0.0217 |
| PRPS1 | Ribose-phosphate pyrophosphokinase 1 | P60891 | 14.8 | 0 | 5 | 0 | 34.834 | 286110000 | 21 | 0.0565 |
| PSAP | Proactivator polypeptide | P07602 | 9.7 | 9.7 | 6 | 6 | 58.112 | 1483900000 | 40 | 0.0660 |
| PSMA3 | Proteasome subunit alpha type-3 | P25788 | 26.3 | 26.3 | 7 | 7 | 28.433 | 292330000 | 28 | 0.0554 |
| PSMA4 | Proteasome subunit alpha type-4 | H0YN18 | 21.3 | 21.3 | 6 | 6 | 26.282 | 373700000 | 26 | 0.0464 |
| PSMA5 | Proteasome subunit alpha type-5 | P28066 | 29.5 | 29.5 | 7 | 7 | 26.411 | 382140000 | 28 | 0.0529 |
| PSMB6 | Proteasome subunit beta type-6 | P28072 | 8.8 | 8.8 | 2 | 2 | 25.357 | 69744000 | 6 | 0.0560 |
| PSMC2 | 26S protease regulatory subunit 7 | P35998 | 13.9 | 13.9 | 6 | 6 | 48.633 | 82664000 | 13 | 0.0386 |
| PSMC5 | 26S protease regulatory subunit 8 | J3QQM1 | 11.4 | 11.4 | 3 | 3 | 29.347 | 81643000 | 15 | 0.0528 |
| PSMD2 | 26S proteasome non-ATPase regulatory subunit 2 | Q13200 | 3.2 | 3.2 | 3 | 3 | 100.2 | 83156000 | 13 | 0.0421 |
| PSME1 | Proteasome activator complex subunit 1 | Q06323 | 49 | 49 | 12 | 12 | 28.723 | 2860900000 | 113 | 0.0451 |
| PSTPIP2 | Proline-serine-threonine phosphatase-interacting protein 2 | Q9H939 | 30.5 | 30.5 | 10 | 10 | 38.858 | 716980000 | 53 | 0.0350 |
| PTBP1 | Polypyrimidine tract-binding protein 1 | A6NLN1 | 19.4 | 19.4 | 9 | 9 | 56.51 | 567820000 | 43 | 0.0535 |
| PTMA | Prothymosin alpha | B8ZZQ6 | 36.4 | 36.4 | 6 | 6 | 11.758 | 753810000 | 57 | 0.0361 |
| PTPN6 | Tyrosine-protein phosphatase non-receptor type 6 | P29350 | 41 | 41 | 22 | 22 | 67.56 | 1100100000 | 82 | 0.0478 |
| PYGB | Glycogen phosphorylase, brain form | P11216 | 29.2 | 20.3 | 24 | 16 | 96.695 | 2431400000 | 116 | 0.0215 |
| RAB10 | Ras-related protein Rab-10 | P61026 | 25.5 | 16.5 | 6 | 4 | 22.541 | 390640000 | 28 | 0.0549 |
| RAB11B | Ras-related protein Rab-11B | Q15907 | 55 | 55 | 13 | 13 | 24.488 | 2263600000 | 89 | 0.0109 |
| RAB14 | Ras-related protein Rab-14 | P61106 | 34.4 | 34.4 | 6 | 6 | 23.897 | 414260000 | 28 | 0.0482 |
| RAB1A | Ras-related protein Rab-1A | E7END7 | 31.2 | 11 | 6 | 2 | 19.018 | 1078600000 | 51 | 0.0576 |
| RAB1B | Ras-related protein Rab-1B | Q9H0U4 | 29.9 | 12.4 | 6 | 2 | 22.171 | 454800000 | 27 | 0.0423 |
| RAB27B | Ras-related protein Rab-27B | O00194 | 43.6 | 43.6 | 9 | 9 | 24.608 | 657400000 | 34 | 0.0601 |
| RAB6A | Ras-related protein Rab-6A | P20340 | 22.1 | 22.1 | 5 | 5 | 23.593 | 1522800000 | 43 | 0.0581 |
| RAB7A | Ras-related protein Rab-7a | P51149 | 51.7 | 51.7 | 9 | 9 | 23.489 | 1173100000 | 56 | 0.0686 |
| RAB8A | Ras-related protein Rab-8A | P61006 | 32.9 | 23.7 | 7 | 5 | 23.668 | 2005800000 | 44 | 0.0703 |
| RAC1 | Ras-related C3 botulinum toxin substrate 1 | P63000 | 47.4 | 19.3 | 8 | 3 | 21.45 | 4589600000 | 91 | 0.0112 |
| RAC2 | Ras-related C3 botulinum toxin substrate 2 | P15153 | 40.1 | 12 | 7 | 2 | 21.429 | 682120000 | 28 | 0.0626 |
| RAN | GTP-binding nuclear protein Ran | P62826 | 29.6 | 29.6 | 6 | 6 | 24.423 | 2947500000 | 89 | 0.0093 |
| RAP1B | Ras-related protein Rap-1b | P61224 | 50 | 15.2 | 10 | 3 | 20.825 | 19691000000 | 156 | 0.0177 |
| RARRES2 | Retinoic acid receptor responder protein 2 | Q99969 | 21.5 | 21.5 | 3 | 3 | 18.617 | 251000000 | 18 | 0.0658 |
| RBMX | RNA-binding motif protein, X chromosome | P38159 | 22.5 | 22.5 | 8 | 8 | 42.331 | 469840000 | 49 | 0.0347 |
| RBP4 | Retinol-binding protein 4 | Q5VY30 | 24.6 | 24.6 | 5 | 5 | 22.974 | 508070000 | 24 | 0.0619 |
| RGS10 | Regulator of G-protein signaling 10 | O43665 | 19.7 | 19.7 | 3 | 3 | 20.236 | 255930000 | 22 | 0.0380 |
| RHOA | Transforming protein RhoA | P61586 | 28.5 | 22.8 | 5 | 4 | 21.768 | 3338600000 | 74 | 0.0145 |
| RNH1 | Ribonuclease inhibitor | P13489 | 33.2 | 33.2 | 12 | 12 | 49.973 | 1607200000 | 91 | 0.0415 |
| RPL10 | 60S ribosomal protein L10 | H7C123 | 40.7 | 40.7 | 4 | 4 | 10.028 | 130100000 | 19 | 0.0458 |
| RPL10A | 60S ribosomal protein L10a | P62906 | 42.4 | 42.4 | 8 | 8 | 24.831 | 417780000 | 23 | 0.0664 |
| RPL12 | 60S ribosomal protein L12 | P30050 | 35.8 | 35.8 | 4 | 4 | 17.818 | 256640000 | 18 | 0.0460 |
| RPL13 | 60S ribosomal protein L13 | P26373 | 32.7 | 32.7 | 7 | 7 | 24.261 | 768180000 | 55 | 0.0505 |
| RPL15 | 60S ribosomal protein L15 | P61313 | 24 | 24 | 5 | 5 | 24.146 | 330080000 | 17 | 0.0360 |
| RPL18 | 60S ribosomal protein L18 | G3V203 | 27.4 | 27.4 | 4 | 4 | 18.756 | 692490000 | 38 | 0.0676 |
| RPL18A | 60S ribosomal protein L18a | M0R3D6 | 37.6 | 37.6 | 5 | 5 | 16.714 | 369020000 | 31 | 0.0479 |
| RPL22 | 60S ribosomal protein L22 | K7EJT5 | 51.1 | 51.1 | 2 | 2 | 5.0827 | 365580000 | 18 | 0.0633 |
| RPL23 | 60S ribosomal protein L23 | P62829 | 37.9 | 37.9 | 4 | 4 | 14.865 | 205210000 | 17 | 0.0539 |
| RPL23A | 60S ribosomal protein L23a | P62750 | 25.6 | 25.6 | 4 | 4 | 17.695 | 439900000 | 24 | 0.0565 |
| RPL24 | 60S ribosomal protein L24 | C9JXB8 | 32.2 | 32.2 | 4 | 4 | 14.369 | 462790000 | 38 | 0.0439 |
| RPL27 | 60S ribosomal protein L27 | P61353 | 36 | 36 | 4 | 4 | 15.798 | 479760000 | 29 | 0.0521 |
| RPL27A | 60S ribosomal protein L27a | E9PLL6 | 42.6 | 42.6 | 4 | 4 | 12.201 | 322210000 | 20 | 0.0504 |
| RPL3 | 60S ribosomal protein L3 | P39023 | 24.1 | 24.1 | 10 | 10 | 46.108 | 426390000 | 38 | 0.0494 |
| RPL34 | 60S ribosomal protein L34 | P49207 | 35.9 | 35.9 | 5 | 5 | 13.293 | 312380000 | 29 | 0.0384 |
| RPL36 | 60S ribosomal protein L36 | Q9Y3U8 | 20 | 20 | 2 | 2 | 12.254 | 224600000 | 14 | 0.0460 |
| RPL4 | 60S ribosomal protein L4 | P36578 | 12.9 | 12.9 | 5 | 5 | 47.697 | 354860000 | 28 | 0.0491 |
| RPL5 | 60S ribosomal protein L5 | P46777 | 17.2 | 17.2 | 5 | 5 | 34.362 | 225830000 | 20 | 0.0520 |
| RPL6 | 60S ribosomal protein L6 | Q02878 | 25.7 | 25.7 | 8 | 8 | 32.728 | 423710000 | 34 | 0.0434 |
| RPL7 | 60S ribosomal protein L7 | P18124 | 19.4 | 19.4 | 4 | 4 | 29.225 | 380340000 | 22 | 0.0606 |
| RPL7A | 60S ribosomal protein L7a | Q5T8U3 | 26.2 | 26.2 | 6 | 6 | 21.545 | 344830000 | 18 | 0.0611 |
| RPL8 | 60S ribosomal protein L8 | P62917 | 22.2 | 22.2 | 6 | 6 | 28.024 | 1020300000 | 43 | 0.0486 |
| RPL9 | 60S ribosomal protein L9 | D6RAN4 | 9.4 | 9.4 | 2 | 2 | 20.775 | 275060000 | 21 | 0.0445 |
| RPLP0 | 60S acidic ribosomal protein P0 | P05388 | 35.6 | 8.2 | 9 | 2 | 34.273 | 991520000 | 55 | 0.0377 |
| RPLP2 | 60S acidic ribosomal protein P2 | P05387 | 76.5 | 76.5 | 6 | 6 | 11.665 | 1701900000 | 59 | 0.0427 |
| RPS12 | 40S ribosomal protein S12 | P25398 | 39.4 | 39.4 | 5 | 5 | 14.515 | 901230000 | 36 | 0.0711 |
| RPS13 | 40S ribosomal protein S13 | P62277 | 25.2 | 25.2 | 4 | 4 | 17.222 | 387840000 | 28 | 0.0575 |
| RPS14 | 40S ribosomal protein S14 | P62263 | 22.5 | 22.5 | 3 | 3 | 16.273 | 439010000 | 29 | 0.0461 |
| RPS15A | 40S ribosomal protein S15a | I3L3P7 | 17 | 17 | 2 | 2 | 11.477 | 423100000 | 19 | 0.0612 |
| RPS16 | 40S ribosomal protein S16 | M0R210 | 41.1 | 41.1 | 6 | 6 | 14.419 | 412920000 | 25 | 0.0488 |
| RPS18 | 40S ribosomal protein S18 | P62269 | 38.2 | 38.2 | 7 | 7 | 17.718 | 618450000 | 49 | 0.0533 |
| RPS19 | 40S ribosomal protein S19 | P39019 | 40 | 40 | 6 | 6 | 16.06 | 1085700000 | 60 | 0.0396 |
| RPS2 | 40S ribosomal protein S2 | H0YEN5 | 33.8 | 33.8 | 6 | 6 | 21.154 | 704440000 | 40 | 0.0491 |
| RPS21 | 40S ribosomal protein S21 | Q8WVC2 | 42 | 42 | 3 | 3 | 8.85 | 354110000 | 17 | 0.0547 |
| RPS25 | 40S ribosomal protein S25 | P62851 | 28 | 28 | 4 | 4 | 13.742 | 573660000 | 21 | 0.0619 |
| RPS28 | 40S ribosomal protein S28 | P62857 | 46.4 | 46.4 | 3 | 3 | 7.8409 | 291290000 | 23 | 0.0428 |
| RPS3 | 40S ribosomal protein S3 | P23396 | 56 | 56 | 14 | 14 | 26.688 | 1505100000 | 96 | 0.0579 |
| RPS3A | 40S ribosomal protein S3a | P61247 | 37.9 | 37.9 | 10 | 10 | 29.945 | 1057700000 | 55 | 0.0594 |
| RPS4X | 40S ribosomal protein S4, X isoform | P62701 | 36.9 | 36.9 | 10 | 10 | 29.597 | 467440000 | 40 | 0.0549 |
| RPS6 | 40S ribosomal protein S6 | P62753 | 13.7 | 13.7 | 4 | 4 | 28.68 | 162230000 | 21 | 0.0460 |
| RPS8 | 40S ribosomal protein S8 | Q5JR95 | 35.6 | 35.6 | 6 | 6 | 21.879 | 440840000 | 30 | 0.0656 |
| RPS9 | 40S ribosomal protein S9 | P46781 | 46.4 | 46.4 | 10 | 10 | 22.591 | 1214400000 | 46 | 0.0689 |
| RPSA | 40S ribosomal protein SA | C9J9K3 | 31.9 | 31.9 | 7 | 7 | 29.404 | 1353000000 | 70 | 0.0209 |
| RSU1 | Ras suppressor protein 1 | Q15404 | 41.9 | 41.9 | 12 | 12 | 31.54 | 8841700000 | 182 | 0.0153 |
| RTN1 | Reticulon-1 | A8MT72 | 8.3 | 8.3 | 2 | 2 | 21.862 | 124510000 | 16 | 0.0471 |
| RTN4 | Reticulon-4 | F8W914 | 14.2 | 14.2 | 4 | 4 | 37.144 | 829250000 | 24 | 0.0738 |
| S100A11 | Protein S100-A11 | P31949 | 34.3 | 34.3 | 4 | 4 | 11.74 | 1316100000 | 38 | 0.0734 |
| S100A4 | Protein S100-A4 | P26447 | 36.6 | 36.6 | 5 | 5 | 11.728 | 7308900000 | 103 | 0.0360 |
| S100A6 | Protein S100-A6 | R4GN98 | 25.9 | 25.9 | 3 | 3 | 9.681 | 4157800000 | 35 | 0.1057 |
| S100A7 | Protein S100-A7 | P31151 | 65.3 | 11.9 | 8 | 2 | 11.471 | 4003200000 | 66 | 0.0846 |
| S100A8 | Protein S100-A8 | P05109 | 48.4 | 48.4 | 6 | 6 | 10.834 | 30550000000 | 128 | 0.0297 |
| S100A9 | Protein S100-A9 | P06702 | 63.2 | 63.2 | 7 | 7 | 13.242 | 24724000000 | 190 | 0.0339 |
| SAA4 | Serum amyloid A-4 protein | P35542 | 27.7 | 27.7 | 4 | 4 | 14.746 | 836890000 | 48 | 0.0308 |
| SAMHD1 | Deoxynucleoside triphosphate triphosphohydrolase SAMHD1 | Q9Y3Z3 | 18.7 | 18.7 | 11 | 11 | 72.2 | 525830000 | 41 | 0.0523 |
| SAR1A | GTP-binding protein SAR1a | Q9NR31 | 19.7 | 19.7 | 4 | 4 | 22.367 | 413820000 | 23 | 0.0560 |
| SCP2 | Non-specific lipid-transfer protein | P22307 | 7.5 | 7.5 | 4 | 4 | 58.993 | 630040000 | 24 | 0.0621 |
| SDPR | Serum deprivation-response protein | O95810 | 43.5 | 43.5 | 16 | 16 | 47.173 | 18992000000 | 267 | 0.0156 |
| SEC22B | Vesicle-trafficking protein SEC22b | O75396 | 11.2 | 11.2 | 2 | 2 | 24.593 | 108040000 | 9 | 0.0421 |
| SELP | P-selectin | Q5R345 | 10.4 | 10.4 | 7 | 7 | 84.33 | 652560000 | 33 | 0.0559 |
| SEPT2 | Septin-2 | C9J2Q4 | 33.7 | 28.8 | 6 | 5 | 21.087 | 509530000 | 28 | 0.0508 |
| SEPT5 | Septin-5 | Q99719 | 18.7 | 18.7 | 7 | 7 | 42.777 | 557090000 | 41 | 0.0383 |
| SEPT6 | Septin-6 | B1AMS2 | 29.2 | 29.2 | 10 | 10 | 49.303 | 943710000 | 59 | 0.0502 |
| SEPT7 | Septin-7 | E7ES33 | 25.2 | 15.6 | 11 | 7 | 48.715 | 2686400000 | 118 | 0.0227 |
| SERBP1 | Plasminogen activator inhibitor 1 RNA-binding protein | Q8NC51 | 15.4 | 15.4 | 5 | 5 | 44.965 | 191570000 | 29 | 0.0232 |
| SERPINA1 | Alpha-1-antitrypsin | P01009 | 47.8 | 47.8 | 20 | 20 | 46.736 | 40161000000 | 394 | 0.0213 |
| SERPINA3 | Alpha-1-antichymotrypsin | P01011 | 25.3 | 25.3 | 10 | 10 | 47.65 | 3066500000 | 101 | 0.0573 |
| SERPINB1 | Leukocyte elastase inhibitor | P30740 | 44.3 | 41.7 | 17 | 16 | 42.741 | 3692800000 | 156 | 0.0212 |
| SERPINB12 | Serpin B12 | Q96P63 | 18.3 | 18.3 | 7 | 7 | 46.276 | 322940000 | 17 | 0.0680 |
| SERPINB3 | Serpin B3 | P29508 | 36.2 | 17.7 | 14 | 6 | 44.564 | 790840000 | 61 | 0.0829 |
| SERPINB6 | Serpin B6 | P35237 | 21 | 18.9 | 6 | 5 | 42.621 | 344740000 | 27 | 0.0399 |
| SERPINC1 | Antithrombin-III | P01008 | 29.5 | 20.7 | 13 | 8 | 52.602 | 2548200000 | 111 | 0.0445 |
| SERPIND1 | Heparin cofactor 2 | P05546 | 22.4 | 21 | 12 | 11 | 57.07 | 1061100000 | 62 | 0.0516 |
| SERPINE1 | Plasminogen activator inhibitor 1 | P05121 | 24.6 | 24.6 | 9 | 9 | 45.059 | 377120000 | 26 | 0.0489 |
| SERPINE2 | Glia-derived nexin | P07093 | 25.6 | 25.6 | 10 | 10 | 44.002 | 723310000 | 41 | 0.0549 |
| SERPINF2 | Alpha-2-antiplasmin | P08697 | 23.2 | 23.2 | 11 | 11 | 54.565 | 772090000 | 66 | 0.0514 |
| SERPING1 | Plasma protease C1 inhibitor | P05155 | 20.8 | 20.8 | 9 | 9 | 55.154 | 1055800000 | 53 | 0.0680 |
| SET | Protein SET | A0A0C4DFV9 | 24.8 | 24.8 | 5 | 5 | 31.124 | 962830000 | 42 | 0.0535 |
| SFN | 14-3-3 protein sigma | P31947 | 42.7 | 32.7 | 10 | 7 | 27.774 | 3602100000 | 41 | 0.1041 |
| SFPQ | Splicing factor, proline- and glutamine-rich | P23246 | 13.4 | 13.4 | 9 | 9 | 76.149 | 393380000 | 36 | 0.0349 |
| SH3BGRL | SH3 domain-binding glutamic acid-rich-like protein | O75368 | 55.3 | 55.3 | 5 | 5 | 12.774 | 455710000 | 22 | 0.0549 |
| SH3BGRL3 | SH3 domain-binding glutamic acid-rich-like protein 3 | Q5T123 | 50 | 50 | 5 | 5 | 9.3804 | 2955100000 | 62 | 0.0591 |
| SH3GLB1 | Endophilin-B1 | Q9Y371 | 3 | 3 | 1 | 1 | 40.796 | 14264000 | 1 | 0.0304 |
| SKAP2 | Src kinase-associated phosphoprotein 2 | O75563 | 13.9 | 13.9 | 5 | 5 | 41.216 | 396170000 | 23 | 0.0596 |
| SLC1A2 | Excitatory amino acid transporter 2 | P43004 | 11 | 11 | 4 | 4 | 62.104 | 896340000 | 30 | 0.0736 |
| SLC25A3 | Phosphate carrier protein, mitochondrial | F8VVM2 | 14.8 | 14.8 | 5 | 5 | 36.161 | 855890000 | 29 | 0.0517 |
| SLC25A5 | ADP/ATP translocase 2 | P05141 | 32.2 | 14.4 | 11 | 4 | 32.852 | 3930600000 | 86 | 0.0706 |
| SLC2A3 | Solute carrier family 2, facilitated glucose transporter member 3 | P11169 | 8.9 | 8.9 | 4 | 4 | 53.924 | 733280000 | 33 | 0.0572 |
| SLC4A1 | Band 3 anion transport protein | P02730 | 12.4 | 12.4 | 9 | 9 | 101.79 | 575700000 | 32 | 0.0667 |
| SLC9A3R1 | Na(+)/H(+) exchange regulatory cofactor NHE-RF1 | J3QRP6 | 27.4 | 27.4 | 5 | 5 | 22.871 | 298240000 | 22 | 0.0410 |
| SLPI | Antileukoproteinase | P03973 | 31.8 | 31.8 | 4 | 4 | 14.326 | 257030000 | 21 | 0.0752 |
| SNAP23 | Synaptosomal-associated protein 23 | O00161 | 47.4 | 47.4 | 8 | 8 | 23.354 | 787300000 | 32 | 0.0619 |
| SNAP25 | Synaptosomal-associated protein 25 | P60880 | 28.6 | 28.6 | 7 | 7 | 23.315 | 406090000 | 32 | 0.0552 |
| SNCA | Alpha-synuclein | E7EPV7 | 66.1 | 50.4 | 6 | 4 | 11.777 | 1558200000 | 67 | 0.0503 |
| SND1 | Staphylococcal nuclease domain-containing protein 1 | Q7KZF4 | 19.8 | 19.8 | 15 | 15 | 102 | 558610000 | 42 | 0.0527 |
| SNRNP70 | U1 small nuclear ribonucleoprotein 70 kDa | M0QYR1 | 19.1 | 19.1 | 2 | 2 | 11.476 | 35093000 | 9 | 0.0432 |
| SOD1 | Superoxide dismutase [Cu-Zn] | P00441 | 39 | 39 | 4 | 4 | 15.936 | 902790000 | 37 | 0.0744 |
| SPARC | SPARC | P09486 | 40.3 | 40.3 | 11 | 11 | 34.632 | 13760000000 | 163 | 0.0347 |
| SPRR1A | Cornifin-A | P35321 | 42.7 | 42.7 | 3 | 3 | 9.8774 | 442670000 | 24 | 0.0788 |
| SPRR2D | Small proline-rich protein 2D | P22532 | 90.3 | 30.6 | 6 | 2 | 7.9053 | 580030000 | 32 | 0.0900 |
| SPTAN1 | Spectrin alpha chain, non-erythrocytic 1 | A0A0D9SF54 | 13.5 | 13.5 | 28 | 28 | 282.83 | 874820000 | 87 | 0.0413 |
| SPTBN1 | Spectrin beta chain, non-erythrocytic 1 | Q01082 | 7.6 | 7.2 | 18 | 17 | 274.61 | 496920000 | 44 | 0.0407 |
| SRC | Proto-oncogene tyrosine-protein kinase Src | P12931 | 25.4 | 18.5 | 14 | 10 | 59.834 | 1587800000 | 71 | 0.0693 |
| SRGN | Serglycin | P10124 | 22.2 | 22.2 | 3 | 3 | 17.652 | 14741000000 | 90 | 0.0390 |
| SRSF1 | Serine/arginine-rich splicing factor 1 | Q07955 | 23.4 | 23.4 | 6 | 6 | 27.744 | 339840000 | 24 | 0.0591 |
| SRSF3 | Serine/arginine-rich splicing factor 3 | P84103 | 32.3 | 23.8 | 6 | 5 | 19.329 | 877430000 | 46 | 0.0436 |
| ST13 | Hsc70-interacting protein | P50502 | 22.8 | 22.8 | 8 | 8 | 41.331 | 534390000 | 28 | 0.0527 |
| STIP1 | Stress-induced-phosphoprotein 1 | P31948 | 31.5 | 31.5 | 19 | 19 | 62.639 | 1132200000 | 81 | 0.0159 |
| STK24 | Serine/threonine-protein kinase 24 | B4DR80 | 18.2 | 18.2 | 7 | 7 | 45.835 | 271260000 | 23 | 0.0508 |
| STMN1 | Stathmin | P16949 | 25.5 | 19.5 | 4 | 3 | 17.302 | 378690000 | 22 | 0.0562 |
| STOM | Erythrocyte band 7 integral membrane protein | P27105 | 46.9 | 46.9 | 11 | 11 | 31.73 | 2842100000 | 85 | 0.0927 |
| STX1B | Syntaxin-1B | P61266 | 8.3 | 8.3 | 2 | 2 | 33.244 | 153220000 | 11 | 0.0455 |
| STXBP1 | Syntaxin-binding protein 1 | A0A1B0GWF2 | 31 | 31 | 16 | 16 | 65.215 | 1125400000 | 85 | 0.0565 |
| STXBP2 | Syntaxin-binding protein 2 | Q15833 | 26 | 26 | 14 | 14 | 66.452 | 889560000 | 55 | 0.0520 |
| SUB1 | Activated RNA polymerase II transcriptional coactivator p15 | P53999 | 22.8 | 22.8 | 3 | 3 | 14.395 | 511570000 | 28 | 0.0484 |
| SULT1A1 | Sulfotransferase 1A1 | P50225 | 27.8 | 16.3 | 8 | 4 | 34.165 | 1189200000 | 43 | 0.0672 |
| SUMO2 | Small ubiquitin-related modifier 2 | P61956 | 23.2 | 23.2 | 2 | 2 | 10.871 | 377230000 | 20 | 0.0570 |
| SYN1 | Synapsin-1 | P17600 | 12.9 | 10.6 | 7 | 6 | 74.111 | 391620000 | 37 | 0.0587 |
| SYNCRIP | Heterogeneous nuclear ribonucleoprotein Q | O60506 | 14.9 | 6.9 | 9 | 4 | 69.602 | 196080000 | 25 | 0.0489 |
| SYNE1 | Nesprin-1 | E7ENN3 | 0.3 | 0.3 | 2 | 2 | 964.83 | 1403900000 | 7 | 0.0423 |
| SYT1 | Synaptotagmin-1 | J3KQA0 | 15.8 | 15.8 | 7 | 7 | 47.26 | 366390000 | 28 | 0.0555 |
| TAGLN2 | Transgelin-2 | P37802 | 67.8 | 60.8 | 12 | 11 | 22.391 | 45222000000 | 336 | 0.0136 |
| TAGLN3 | Transgelin-3 | C9J5W6 | 31.3 | 19.1 | 3 | 2 | 12.886 | 12271000 | 3 | 0.0391 |
| TALDO1 | Transaldolase | P37837 | 38.9 | 38.9 | 13 | 13 | 37.54 | 3218400000 | 93 | 0.0162 |
| TCEB2 | Transcription elongation factor B polypeptide 2 | B8ZZU8 | 17.7 | 17.7 | 2 | 2 | 12.527 | 193800000 | 16 | 0.0369 |
| TCP1 | T-complex protein 1 subunit alpha | P17987 | 31.8 | 31.8 | 16 | 16 | 60.343 | 1298700000 | 76 | 0.0529 |
| TF | Serotransferrin | P02787 | 59 | 56.9 | 42 | 40 | 77.063 | 78306000000 | 689 | 0.0270 |
| TFPI | Tissue factor pathway inhibitor | P10646 | 4.6 | 4.6 | 1 | 1 | 35.015 | 16950000 | 2 | 0.0397 |
| TGFB1 | Transforming growth factor beta-1 | P01137 | 37.2 | 37.2 | 14 | 14 | 44.341 | 6750500000 | 169 | 0.0185 |
| TGM3 | Protein-glutamine gamma-glutamyltransferase E | Q08188 | 34.3 | 34.3 | 20 | 20 | 76.631 | 773870000 | 48 | 0.0873 |
| THBS1 | Thrombospondin-1 | P07996 | 60.8 | 20.3 | 64 | 17 | 129.38 | 6.6691E+11 | 2097 | 0.0132 |
| TIMP1 | Metalloproteinase inhibitor 1 | Q5H9A7 | 51.7 | 51.7 | 6 | 6 | 16.057 | 1384200000 | 29 | 0.0774 |
| TIMP3 | Metalloproteinase inhibitor 3 | P35625 | 32.2 | 32.2 | 7 | 7 | 24.145 | 784110000 | 33 | 0.0646 |
| TKT | Transketolase | P29401 | 30.8 | 30.8 | 16 | 16 | 67.877 | 4075300000 | 176 | 0.0177 |
| TLN1 | Talin-1 | Q9Y490 | 61.2 | 55.3 | 136 | 119 | 269.76 | 2.5215E+11 | 2647 | 0.0166 |
| TMOD3 | Tropomodulin-3 | Q9NYL9 | 20.5 | 20.5 | 7 | 7 | 39.594 | 365950000 | 22 | 0.0390 |
| TMSB4X | Thymosin beta-4 | P62328 | 86.4 | 86.4 | 5 | 4 | 5.0526 | 19010000000 | 74 | 0.0811 |
| TNF | Tumor necrosis factor | A0A140T922 | 46.2 | 46.2 | 6 | 6 | 18.862 | 4213500000 | 48 | 0.1174 |
| TPI1 | Triosephosphate isomerase | P60174 | 59.1 | 59.1 | 14 | 14 | 30.791 | 10193000000 | 224 | 0.0100 |
| TPM1 | Tropomyosin alpha-1 chain | P09493 | 51.4 | 4.6 | 22 | 2 | 32.708 | 1364600000 | 62 | 0.0492 |
| TPM1 | Tropomyosin 1 (Alpha), isoform CRA_m | H7BYY1 | 61.3 | 0 | 20 | 0 | 28.747 | 3443100000 | 72 | 0.0722 |
| TPM1 | Tropomyosin 1 (Alpha), isoform CRA_f | Q6ZN40 | 47.9 | 4.9 | 22 | 2 | 37.452 | 888720000 | 19 | 0.0688 |
| TPM3 | Tropomyosin alpha-3 chain | A0A087WWU8 | 70 | 0 | 24 | 0 | 26.42 | 4194700000 | 45 | 0.0440 |
| TPM3 | Tropomyosin alpha-3 chain | J3KN67 | 58.2 | 0 | 25 | 0 | 33.222 | 33797000000 | 337 | 0.0221 |
| TPM4 | Tropomyosin alpha-4 chain | P67936 | 66.1 | 31.5 | 24 | 8 | 28.521 | 26218000000 | 218 | 0.0219 |
| TREML1 | Trem-like transcript 1 protein | Q86YW5 | 12.5 | 12.5 | 3 | 3 | 32.678 | 578820000 | 26 | 0.0664 |
| TSPAN14 | Tetraspanin-14 | H7BXY6 | 13.6 | 13.6 | 3 | 3 | 23.918 | 1634900000 | 16 | 0.0670 |
| TTR | Transthyretin | A0A087WV45 | 51.8 | 51.8 | 5 | 5 | 15.12 | 1600100000 | 47 | 0.0850 |
| TUBA1A | Tubulin alpha-1A chain | Q71U36 | 56.3 | 0 | 21 | 0 | 50.135 | 1137500000 | 20 | 0.0826 |
| TUBA1B | Tubulin alpha-1B chain | P68363 | 56.3 | 0 | 21 | 0 | 50.151 | 98464000000 | 494 | 0.0106 |
| TUBA4A | Tubulin alpha-4A chain | P68366 | 56.7 | 12.1 | 21 | 4 | 49.924 | 11131000000 | 71 | 0.0776 |
| TUBA8 | Tubulin alpha-8 chain | Q9NY65 | 48.6 | 18.3 | 18 | 7 | 50.093 | 1681900000 | 52 | 0.0484 |
| TUBB | Tubulin beta chain | Q5JP53 | 60.1 | 12.7 | 19 | 3 | 47.766 | 10583000000 | 99 | 0.0536 |
| TUBB2A | Tubulin beta-2A chain | Q13885 | 55.3 | 2.7 | 19 | 1 | 49.906 | 1447900000 | 48 | 0.0724 |
| TUBB3 | Tubulin beta-3 chain | Q13509 | 44 | 12 | 16 | 3 | 50.432 | 995550000 | 37 | 0.0375 |
| TUBB4A | Tubulin beta-4A chain | P04350 | 56.5 | 6.3 | 18 | 2 | 49.585 | 569010000 | 32 | 0.0482 |
| TUBB4B | Tubulin beta-4B chain | P68371 | 65.2 | 2.7 | 21 | 1 | 49.83 | 46240000000 | 575 | 0.0108 |
| TWF2 | Twinfilin-2 | Q6IBS0 | 36.7 | 36.7 | 11 | 11 | 39.548 | 1217200000 | 54 | 0.0439 |
| TXN | Thioredoxin | P10599 | 48.6 | 48.6 | 6 | 6 | 11.737 | 2882000000 | 80 | 0.0200 |
| TXNL1 | Thioredoxin-like protein 1 | K7ER96 | 5.7 | 5.7 | 2 | 2 | 31.355 | 162350000 | 12 | 0.0465 |
| TYMP | Thymidine phosphorylase | P19971 | 24.9 | 24.9 | 10 | 10 | 49.955 | 358830000 | 38 | 0.0555 |
| UBA1 | Ubiquitin-like modifier-activating enzyme 1 | P22314 | 24.7 | 24.7 | 22 | 22 | 117.85 | 2088700000 | 126 | 0.0290 |
| UBB | Ubiquitin-60S ribosomal protein L40 | J3QS39 | 50.5 | 50.5 | 5 | 5 | 10.469 | 7662900000 | 90 | 0.0245 |
| UBE2N | Ubiquitin-conjugating enzyme E2 N | P61088 | 38.8 | 14.5 | 6 | 2 | 17.138 | 731180000 | 40 | 0.0569 |
| UBE2V1 | Ubiquitin-conjugating enzyme E2 variant 1 | Q13404 | 29.3 | 29.3 | 5 | 5 | 16.495 | 540360000 | 27 | 0.0646 |
| UTS2 | Urotensin-2 | O95399 | 6.5 | 6.5 | 1 | 1 | 14.295 | 449120000 | 13 | 0.0525 |
| VAMP2 | Vesicle-associated membrane protein 2 | J3QRU4 | 35.4 | 35.4 | 3 | 3 | 12.251 | 538140000 | 29 | 0.0633 |
| VASP | Vasodilator-stimulated phosphoprotein | P50552 | 47.6 | 47.6 | 15 | 15 | 39.829 | 4888800000 | 164 | 0.0179 |
| VCL | Vinculin | P18206 | 61.7 | 61.7 | 69 | 69 | 123.8 | 76533000000 | 1042 | 0.0123 |
| VCP | Transitional endoplasmic reticulum ATPase | P55072 | 27.7 | 27.7 | 21 | 21 | 89.321 | 3168500000 | 167 | 0.0066 |
| VDAC1 | Voltage-dependent anion-selective channel protein 1 | P21796 | 44.2 | 44.2 | 10 | 10 | 30.772 | 811570000 | 38 | 0.0743 |
| VDAC2 | Voltage-dependent anion-selective channel protein 2 | A0A0A0MR02 | 35.1 | 35.1 | 8 | 8 | 30.348 | 668510000 | 48 | 0.0350 |
| VDAC3 | Voltage-dependent anion-selective channel protein 3 | Q9Y277 | 15.2 | 15.2 | 4 | 4 | 30.658 | 479490000 | 20 | 0.0588 |
| VIM | Vimentin | P08670 | 69.7 | 62.7 | 39 | 34 | 53.651 | 23671000000 | 513 | 0.0126 |
| VPS35 | Vacuolar protein sorting-associated protein 35 | Q96QK1 | 9.9 | 9.9 | 7 | 7 | 91.706 | 248600000 | 20 | 0.0473 |
| VSNL1 | Visinin-like protein 1 | P62760 | 22.5 | 22.5 | 4 | 4 | 22.142 | 176380000 | 21 | 0.0556 |
| VTN | Vitronectin | P04004 | 29.9 | 25.1 | 15 | 12 | 54.305 | 5588800000 | 148 | 0.0160 |
| VWF | von Willebrand factor | P04275 | 42.3 | 42.3 | 100 | 100 | 309.26 | 36302000000 | 990 | 0.0228 |
| WDR1 | WD repeat-containing protein 1 | O75083 | 62.9 | 62.9 | 29 | 29 | 66.193 | 14656000000 | 321 | 0.0109 |
| WDR44 | WD repeat-containing protein 44 | Q5JSH3 | 11.1 | 11.1 | 10 | 10 | 101.37 | 305370000 | 33 | 0.0293 |
| XRCC6 | X-ray repair cross-complementing protein 6 | P12956 | 18.7 | 18.7 | 9 | 9 | 69.842 | 247790000 | 25 | 0.0360 |
| YARS | Tyrosine--tRNA ligase, cytoplasmic | A0A0C4DGZ5 | 16.5 | 16.5 | 7 | 7 | 43.904 | 378030000 | 26 | 0.0440 |
| YBX1 | Nuclease-sensitive element-binding protein 1 | P67809 | 24.7 | 24.7 | 5 | 5 | 35.924 | 275860000 | 35 | 0.0347 |
| YES1 | Tyrosine-protein kinase Yes | P07947 | 10.3 | 3.5 | 6 | 2 | 60.801 | 19671000 | 2 | 0.0424 |
| YWHAB | 14-3-3 protein beta/alpha | P31946 | 40.2 | 22.8 | 9 | 4 | 28.082 | 2670800000 | 62 | 0.0267 |
| YWHAE | 14-3-3 protein epsilon | P62258 | 58.8 | 49 | 16 | 13 | 29.174 | 15654000000 | 238 | 0.0120 |
| YWHAG | 14-3-3 protein gamma | P61981 | 43.3 | 29.6 | 12 | 8 | 28.302 | 4343800000 | 160 | 0.0179 |
| YWHAH | 14-3-3 protein eta | Q04917 | 43.5 | 33.3 | 12 | 9 | 28.218 | 2337000000 | 100 | 0.0130 |
| YWHAQ | 14-3-3 protein theta | P27348 | 36.3 | 22.4 | 10 | 6 | 27.764 | 1571500000 | 77 | 0.0252 |
| YWHAZ | 14-3-3 protein zeta/delta | P63104 | 55.5 | 41.6 | 14 | 10 | 27.745 | 22581000000 | 310 | 0.0092 |
| ZG16B | Zymogen granule protein 16 homolog B | A0A0C4DGN4 | 30.3 | 30.3 | 4 | 4 | 19.6 | 2458400000 | 44 | 0.1157 |
| ZYX | Zyxin | Q15942 | 46.5 | 46.5 | 18 | 18 | 61.277 | 12679000000 | 257 | 0.0159 |

**Supplementary Table S4. Regulated proteins identified in monocyte derived EVs in the presence or absence of incubation with Iloprost: comparison with published data on EVs from human platelets and a human monocytic cell line.** Targeted analysis highlighting differences between TNF-α and TNF-α+PGI_2_ EVs identified 33 proteins that were significantly altered. Unique proteins identified in either subset are also indicated.

| **COMMON MODULATED PROTEINS** | | | | | | |
| --- | --- | --- | --- | --- | --- | --- |
| **Protein names** | **Gene names** | **Protein IDs** | **THP-1 EVs**  **J Thrombosis Haemostasis 2009 (1076 pts)** | **PLATELET EVs**  **JEV, 2014**  **(267 pts)** | **PLATELET EVs**  **Transfusion, 2015**  **(1458 pts)** | |
| Core histone macro-H2A.1 | H2AFY | O75367 |  |  | Histone H2A | |
| Caspase-14 | CASP14 | P31944 |  |  |  | |
| Tissue factor pathway inhibitor | TFPI | P10646 |  |  |  | |
| Vesicle-associated membrane protein 2 | VAMP2 | J3QRU4 | Vesicles-associated membrane protein 8 | Vesicles-associated membrane protein 7 |  | |
| 40S ribosomal protein S12 | RPS12 | P25398 | 40S ribosomal protein s11  and s14 |  |  | |
| Synaptotagmin-1 | SYT1 | J3KQA0 |  | Synaptotagmin-4 |  | |
| Cytochrome c1, heme protein, mitochondrial | CYC1 | P08574 | ● |  | ● | |
| Vacuolar protein sorting-associated protein 35 | VPS35 | Q96QK1 | ● |  | ● | |
| 14-3-3 protein sigma | SFN | P31947 | Other isoforms | Other isoforms | Other isoforms | |
| PDZ and LIM domain protein 7 | PDLIM7 | Q9NR12 |  | ● | ● | |
| X-ray repair cross-complementing protein 6 | XRCC6 | P12956 |  |  |  | |
| Ras-related protein Rab-7a | RAB7A | P51149 | ● | ● | ● | |
| ADP-ribosylation factor 1 | ARF1 | P84077 | ● | Other isoforms | ● | |
| Rho GTPase-activating protein 6 | ARHGAP6 | O43182 |  |  | ● | |
| Tropomodulin-3 | TMOD3 | Q9NYL9 |  |  |  | |
| Immunoglobulin heavy variable 3/OR16-12 | IGHV3OR16-12 | A0A075B7B8 |  |  |  | |
| Aspartate--tRNA ligase, cytoplasmic | DARS | P14868 |  |  | ● | |
| Pyruvate kinase PKM | PKM | P14618 |  |  | ● | |
| Serine/threonine-protein kinase PAK 2 | PAK2 | Q13177 | Serine/threonine-protein kinase 10 |  | Serine/threonine-protein kinase 3 | |
| Extracellular matrix protein 1 | ECM1 | Q16610 |  |  |  | |
| Cytochrome c oxidase subunit 5A, mitochondrial | COX5A | H3BRM5 | ● |  | ● | |
| Leukocyte surface antigen CD47 | CD47 | Q08722 |  | ● | ● | |
| Microtubule-associated protein 2 | MAP2 | P11137 |  |  |  | |
| Pleckstrin | PLEK | P08567 |  | ● | ● | |
| Protein disulfide-isomerase A6 | PDIA6 | Q15084 | ● |  | ● | |
| Ig heavy chain V-I region V35 | IGHV1-2 | P23083 |  |  |  | |
| Complement C5 | C5 | P01031 |  |  |  | |
| Rho GTPase-activating protein 1 | ARHGAP1 | Q07960 |  | ● | ● | |
| Twinfilin-2 | TWF2 | Q6IBS0 |  |  | ● | |
| Small ubiquitin-related modifier 2 | SUMO2 | P61956 |  |  |  | |
| Gelsolin | GSN | P06396 | ● | ● |  | |
| Salivary acidic proline-rich phosphoprotein 1/2 | PRH1 | A0A087WYF5 |  |  |  | |
| T-complex protein 1 subunit epsilon | CCT5 | P48643 | ● |  | ● | |
| **UNIQUE PROTEINS** | | | | | | |
| **Protein names** | **Gene names** | **Protein IDs** | **THP-1 EVs**  **J Thrombosis Haemostasis 2009 (1076 pts)** | **PLATELET EVs**  **JEV, 2014**  **(267 pts)** | | **PLATELET EVs**  **Transfusion, 2015**  **(1458 pts)** |
| **TNF-α EVs** | | | | | | |
| U1 small nuclear ribonucleoprotein 70 kDa | SNRNP70 | M0QYR1 |  |  | |  |
| Core histone macro-H2A.1 | H2AFY | O75367 |  |  | |  |
| Involucrin | IVL | P07476 |  |  | |  |
| Ganglioside GM2 activator | GM2A | P17900 |  |  | |  |
| Elafin | PI3 | P19957 |  |  | |  |
| Elongation factor 1-beta | EEF1B2 | P24534 | ● |  | |  |
| Proteasome subunit beta type-6 | PSMB6 | P28072 |  | ● | |  |
| 26S protease regulatory subunit 7 | PSMC2 | P35998 | ● |  | | ● |
| Enhancer of rudimentary homolog | ERH | P84090 |  |  | |  |
| AP-2 complex subunit mu | AP2M1 | A0A087WY71 |  |  | | ● |
| **TNF-α+PGI_2_ EVs** | | | | | | |
| Immunoglobulin K Variable 2-24 | IGKV2-24 |  |  |  | |  |
| Alpha-internexin | INA | Q16352 |  |  | | ● |

● – Indicates common hit between the different studies. When a member of the family is identified, the full name is given.

**Supplementary Table S5. Demographic characteristic and treatments of coronary artery patients.** Plasma sample were used to isolate and characterise EVs from monocyte and platelets.

| **Characteristic** | **No PCI* (N=12)** | **PCI (N=12)** |
| --- | --- | --- |
| Age mean (SD), years | 62.17 (6.92) | 67.08 (10.33) |
| Male n (%) | 6 (50%) | 6 (50%) |
| SBP mean (SD), mmHg | 142.6 (17.14) | 157 (25.70) |
| DBP mean (SD),mmHg | 79.42 (9.45) | 84.83 (12.43) |
| BMI mean (SD),kg/m2 | 29.49 (5.15) | 28.36 (3.53) |
| Cholesterol lowering therapy (%) | 8 (66.7%) | 8 (66.7%) |
| Anticoagulant therapy (%) | 6(50.0%) | 6 (50.0%) |

PCI: percutaneous coronary intervention,.
